# Downregulation of PPARα mediates FABP1 expression, contributing to IgA nephropathy by stimulating ferroptosis in human mesangial cells

**DOI:** 10.7150/ijbs.74675

**Published:** 2022-08-29

**Authors:** Jingkui Wu, Xinghua Shao, Jianxiao Shen, Qisheng Lin, Xuying Zhu, Shu Li, Jialin Li, Wenyan Zhou, Chaojun Qi, Zhaohui Ni

**Affiliations:** Department of Nephrology, Renji Hospital, School of Medicine, Shanghai Jiao Tong University, Shanghai, China

**Keywords:** Immunoglobulin A nephropathy, Peroxisome proliferator-activated receptor α, Fatty acid binding protein 1, Ferroptosis, Human mesangial cells, Weighted gene correlation network analysis

## Abstract

Immunoglobulin A nephropathy (IgAN) is the commonest primary glomerulonephritis, and a major cause of end-stage renal disease; however, its pathogenesis requires elucidation. Here, a hub gene, *FABP1*, and signaling pathway, PPARα, were selected as key in IgAN pathogenesis by combined weighted gene correlation network analysis of clinical traits and identification of differentially expressed genes from three datasets. FABP1 and PPARα levels were lower in IgAN than control kidney, and linearly positively correlated with one another, while FABP1 levels were negatively correlated with urinary albumin-to-creatinine ratio, and GPX4 levels were significantly decreased in IgAN. In human mesangial cells (HMCs), PPARα and FABP1 levels were significantly decreased after Gd-IgA1 stimulation and mitochondria appeared structurally damaged, while reactive oxygen species (ROS) and malondialdehyde (MDA) were significantly increased, and glutathione and GPX4 decreased, relative to controls. GPX4 levels were decreased, and those of ACSL4 increased on siPPARα and siFABP1 siRNA treatment. In PPARα lentivirus-transfected HMCs stimulated by Gd-IgA1, ROS, MDA, and ACSL4 were decreased; glutathione and GPX4, and immunofluorescence colocalization of PPARα and GPX4, increased; and damaged mitochondria reduced. Hence, PPARα pathway downregulation can reduce FABP1 expression, affecting GPX4 and ACSL4 levels, causing HMC ferroptosis, and contributing to IgAN pathogenesis.

## Introduction

Immunoglobulin A nephropathy (IgAN) is the most common type of primary glomerulonephritis globally, which is a major cause of end-stage renal disease 1, 2 . Moreover, approximately 30%-40% of kidney biopsies confirm IgAN, with frequencies in Asia ranging from 22% to 39%, which is higher than those observed in Europe 3, 4. IgA-dominant immunoglobulin deposition in the glomerular mesangial area of the kidney is considered the main cause of IgAN; however, the specific pathogenesis underlying IgAN remains to be elucidated 5, 6.

It is established that accumulation of immune complexes, such as IgA1 and/or IgA1-IgG, in the glomerular mesangial area leads to IgAN kidney injury 1. Generally, changes (upregulation or downregulation) in gene expression, can be reflected or observed at the histological level; hence, causes of kidney injury can be investigated by identifying differentially expressed genes (DEGs) using bioinformatic analyses 7. Therefore, we identified DEGs from IgAN datasets, then used weighted gene correlation network analysis (WGCNA) to analyze a dataset comprising clinical features of patients with IgAN, to screen for key factors related to urinary albumin-to-creatinine ratio (ACR). Further, we used renal tissue samples from patients with IgAN and healthy controls to experimentally verify hub genes identified as involved in ACR by bioinformatics approaches. Moreover, we assessed the effects of the key genes and signaling pathways in human mesangial cells (HMCs) in vitro.

The aim of this research was to identify key genes and signaling pathways related to IgAN clinical traits via comprehensive bioinformatics analysis. Finally, we identified the gene, *FABP1*, and the peroxisome proliferator-activated receptor (PPAR) signaling pathway, comprising PPARα, PPARβ, and PPARγ, as important in this context. PPARα levels are decreased in a unilateral ureteral obstruction (UUO) rat model 8, and PPARα signaling is disrupted in aging kidney during accelerated renal fibrosis 9. Therefore, we conducted further research on the PPARα signaling pathway. PPARα is a member of the PPAR family that functions as a nuclear transcription factor in fatty acid metabolism 10, and *FABP1* is a downstream target gene in PPARα signaling that participates in fatty acid transport 11, 12. Fatty acid binding protein 1 (FABP1, or FABPL, L-FABP) is a 14.4 kDa protein expressed in human proximal tubules that regulates the cellular uptake, transport, and metabolism of fatty acids 13. Importantly, FABP1 has a protective role in acute kidney injury (AKI) and chronic kidney disease (CKD) 14, and can decrease glomerular injury at the early stage of IgAN in animal experiments 15.

Ferroptosis is an important, newly-discovered form of cell death caused by glutathione peroxidase 4 (GPX4) deficiency and associated with oxidation and iron dependence. A key feature of ferroptosis is the accumulation of lipid peroxides on cellular membranes, which distinguishes this type of cell death from the classical processes of necrosis, autophagy, and apoptosis 16, 17. Ferroptosis participates in the pathogenesis of various kidney diseases, including AKI 18, renal fibrosis 19, polycystic kidney disease 20, and diabetic nephropathy 21. Ferroptosis occurrence is related to GPX4 consumption and increased production of ROS and other oxides. By contrast, increased cellular FABP1 is associated with decreased ROS production 12 and increased PPARα activity can suppress ferroptosis, while decreased PPARα activity increases sensitivity to ferroptosis; notably overexpression of PPARα can suppress ferroptotic cell death 22. Nevertheless, the link between ferroptosis and IgAN remains unclear. Therefore, we used galactose deficient IgA1 (Gd-IgA1), extracted from patients with IgAN, to stimulate HMCs and establish an IgAN model in vitro, then conducted various experiments to explore the effect of PPARα signaling and ferroptosis on HMCs.

## Materials and methods

### Study design

**Figure 1:**
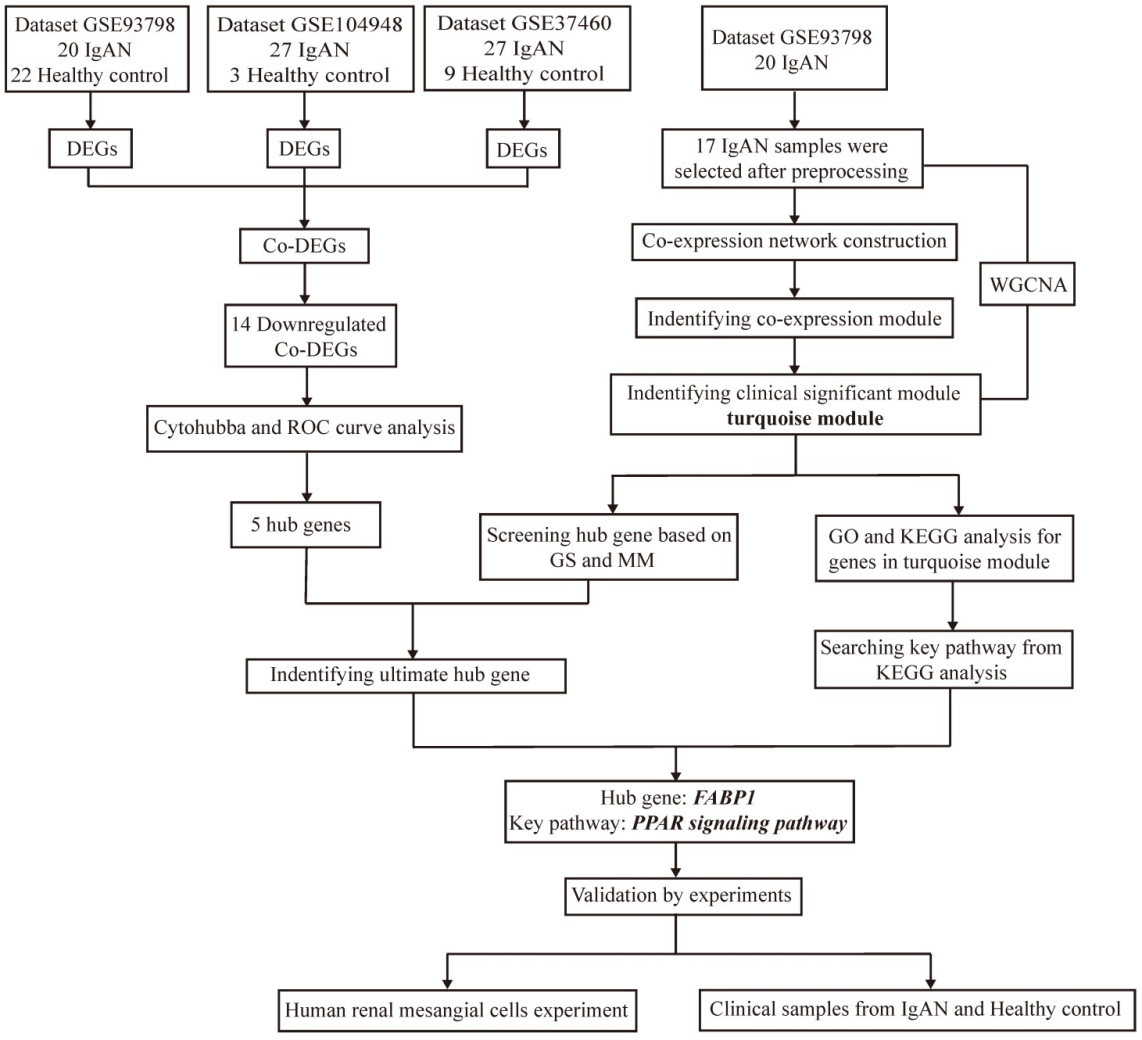
Flow diagram of the study design and workflow.

Target datasets in the Gene Expression Omnibus (GEO; http://www.ncbi.nlm.nih.gov/geo/), were filtered using “IgA nephropathy” as the keyword, “Homo sapiens” as the organism, “expression profiling by array” as the data type, and “June 30, 2020” as the latest publication date, resulting in identification of 23 datasets (Supplementary Table S1) derived from different specimens (glomerular, tubulointerstitial, peripheral blood). As the major lesion in IgAN is in the glomerular mesangium, datasets related to glomeruli were selected for inclusion in this study, resulting in three included datasets, following exclusion of those from tubulointerstitium or peripheral blood, and those lacking controls. The design and workflow of this study are illustrated in Figure 1. First, the GEO datasets, GSE93798 23, GSE37460^24^, and GSE104948^25^, comprising data from renal tissue from patients with IgAN and healthy controls, were screened for DEGs, then DEGs common among the datasets identified. Among the three datasets, GSE93798 contained 22 healthy controls and 20 patients with IgAN, GSE37460 had 9 healthy controls and 27 patients with IgAN, and there were 3 healthy controls and 27 patients with IgAN in GSE104948 (Supplementary Table S2). Hub genes were identified among downregulated DEGs using cytoHubba and receiver operating characteristic (ROC) curve analysis. WGCNA of the GSE93798 dataset was used to identify key factors related to ACR, then hub genes were selected using the threshold values: gene significance (GS) > 0.2 and module membership (MM) > 0.7. Finally, co-hub genes were identified based on the results of both analyses described above. Genes identified as significant were analyzed for Gene Ontology (GO) and Kyoto Encyclopedia of Genes and Genomes (KEGG) enrichment, to identify significantly enriched signaling pathways. Then, in vitro experiments were conducted using clinical specimens from patient with IgAN and controls, as well as HMCs, to verify the hub genes and signaling pathways identified by bioinformatics and explore the role of ferroptosis in IgAN.

### Screening for Co-DEGs

The GSE93798, GSE37460, and GSE104948 datasets were screened for DEGs using the “limma” package in R v4.0.0 software (cutoff values: adjusted P < 0.05 and |log_2_(fold-change)|) ≥ 1). Expression levels of all genes in the three datasets are presented as volcano plots and the top 50 DEGs are illustrated using a hierarchical cluster heatmap. Downregulated DEGs were selected for further analysis; genes screened from all three datasets were considered co-DEGs.

### Construction of protein-protein interaction (PPI) networks and identification of hub genes

PPI networks of co-DEGs were constructed using Search Tool for the Retrieval of Interacting Genes/Proteins (STRING, http://string-db.org/)^26^ and analyzed using cytoHubba, with 12 algorithms applied to evaluate hub genes. Then four algorithms, for analyses of stress, betweenness 27, 28, degree, and radiality 29, were applied to identify hub genes. The top 5 genes among 14 downregulated co-DEGs after evaluation using the four algorithms were considered significant hub genes.

The “sva” package in R v4.0.0 software was used to remove batch effects on the initial expression levels of the five selected hub genes and violin plots were drawn to evaluate the expression levels of these genes, using “ggplot” and “ggpubr”. The sensitivity and specificity of these hub genes for diagnosing IgAN were evaluated by ROC curve analysis using the “pROC” package.

### WGCNA of IgAN samples from GSE93798

Among the three datasets analyzed in this study, only GSE93798 included detailed information on clinical traits of 20 patients with IgAN (Supplementary Table S3 23), and WGCNA was conducted using this dataset. After screening, only 17 IgAN samples were considered suitable for inclusion in WGCNA. The top 5000 genes with the largest variance in GSE93798 were analyzed by applying the “WGCNA” package. Hierarchical clustering analysis was conducted to identify outliers among the samples and scale-free networks constructed using an appropriate soft threshold power. Adjacency matrix and topologic overlap matrix (TOM) were then constructed, and the corresponding dissimilarity (1-TOM) computed. Minimum module size in dynamic tree cutting was set at 50, then a dendrogram of genes and modules generated. Dissimilarity was set to 0.25, highly similar modules merged by clustering of module eigengenes 30, and 1000 of the top 5000 genes randomly selected to construct a network heatmap plot. Correlations between module eigengenes and IgAN clinical traits were analyzed, and modules strongly associated with ACR chosen for further analysis. Further, genes with high GS and MM values for ACR were considered key genes in these modules.

### Analysis of biological processes and pathways enriched for significant co-expression modules

GO function and KEGG pathway enrichment analyses for genes in modules significantly associated with clinical traits were performed using the DAVID online database 31, with the threshold values, P < 0.01 and gene count ≥ 2.

### Antibodies and reagents

PPAR-α antibody (sc-398394) was from Santa Cruz Biotechnology (Santa Cruz, USA). Horseradish peroxidase-conjugated goat anti-mouse IgG H&L (ab6789) and goat anti-rabbit IgG H&L (ab205718), as well as GPX4 (ab125066), ACSL4 (ab155282), and FABP1 (ab170950) antibodies, were from Abcam (Cambridge, MA, USA). Erastin (HY-15763) and ferrostain-1 (HY-100579) were from MedChemExpress (Monmouth Junction, NJ). Glutathione (GSH; A006-2-1) and malondialdehyde (MDA) (A003-4-1) were from Jiancheng Bioengineering Institute (Nanjing, Jiangsu, China). GAPDH (AF5009), Tubulin (AF1216), and MnSOD (S0103) antibodies were from Beyotime Biotechnology (Shanghai, China). Cell Counting Kit 8 (CCK‐8, CK04) kits were from Dojindo (Shanghai, China). Lentiviral vectors (PGMLV- CMV-MCS-EF1-mScarlet-T2A-Puro, GM-18458), encoding PPARα or a null control with flag tag, and small interfering RNA (siRNA), were obtained from Genomeditech (Shanghai, China). Sequences of siRNAs were as follows: PPARα, 5'-UGAACUUCAUGGCAAAAUCAA-3' and FABP1, 5'-ACUUUCUCCCCUGUCAUUGUC-3'.

### Experiments on renal specimens

#### Collection of renal tissue specimens

To ensure recruitment of suitable patients with IgAN, exclusion criteria for this study were patients with diabetes, hepatitis, cirrhosis, systemic lupus erythematosus, or secondary IgAN. Basic clinical information from 108 patients diagnosed with IgAN after renal biopsy at the Nephrology Department of Renji hospital from July 2020 to April 2021 were collected, along with consent of patients to participate in this study. Ultimately, 63 patients with IgAN met our research requirements (Supplementary Table S4) and paraffin sections from these patients were collected after diagnostic renal biopsy. In addition, 12 inpatients with kidney cancer were recruited from the Department of Urology, Renji Hospital (Supplementary Table S5), and renal tissue samples collected from 5 cm adjacent to the cancer tissue of these patients, used as control specimens.

#### Immunohistochemistry and immunofluorescence detection of glomeruli in renal tissue

To evaluate PPARα and FABPI expression in the glomerular mesangial region, paraffin sections of renal tissues from patients with IgAN and control subjects were analyzed by immunohistochemistry (IHC). GPX4 expression in mesangial renal tissue specimens was detected and observed by immunofluorescence, to explore the occurrence of ferroptosis in IgAN. Two pathologists evaluated five random positively stained fields, and semi-quantitative analysis of proteins in the glomerular region was conducted using Image-Pro Plus v6.0. Pearson analysis was used to assess the correlation between PPARα and FABP1 expression in mesangial renal tissue.

#### Extraction of Gd-IgA1 from peripheral blood samples from patients with IgAN

Peripheral blood samples were collected from patients with IgAN, Gd-IgA1 extracted according to previously described methods 32, and used for subsequent HMC experiments.

### Experiments using HMCs

#### HMC culture

HMCs (FH0241) were obtained from FuHeng Biology (Shanghai, China) and cultured in Dulbecco's Modified Eagle's Medium containing 10% fetal bovine serum (FBS). HMCs were cultured for two passages before use in experiments.

#### Induction of ferroptosis

HMCs were treated with different concentrations of erastin (0, 5, 10, 15, 20 μg/mL) for 12 h to induce ferroptosis. HMC viability was assessed using CCK-8 kits, following the manufacturer's instructions, to determine the appropriate erastin concentration for HMC treatment. To evaluate the effect of ferrostatin-1 (Fer-1) on ferroptosis, 2 μM Fer-1 was added to cells 12 h prior to erastin treatment. To evaluate the effects of PPARα and FABP1 on ferroptosis, HMCs were pre-transfected with overexpression plasmids or siRNA targeting these factors for 48 h prior to erastin treatment.

#### Stimulation of HMCs with Gd-IgA1

HMCs were stimulated with extracted Gd-IgA1 to establish an in vitro IgAN model; the following Gd-IgA1 concentrations were used: 1 μg/mL, 10 μg/mL, 100 μg/mL, 1 mg/mL, 10 mg/mL, and 100 mg/mL. When HMCs reached 60% confluence, they were cultured in serum-free medium and stimulated with different concentrations of Gd-IgA1 for 24 h. HMC viability was assessed using CCK-8 kits, following the manufacturer's instructions, to identify an appropriate Gd-IgA1 concentration for use in further experiments. HMCs were treated with siPPARα or siFABP1 for 48 h prior to Gd-IgA1 stimulation.

#### PPARα overexpression by lentivirus transfection

Lentiviral vectors (PGMLV-CMV-EF1-mScarlet-T2A-Puro, GM-18458) encoding PPARα or null control with flag tags were obtained from Genomeditech (Shanghai, China), to generate PPARα (PGMLV-CMV-H_PPARα-EF1-mScarlet-T2A-Puro, ID 38189) and control (5E7TU) lentivirus. Two HMC experimental groups were used: the PPARα-OE group was transfected with PPARα lentivirus, and the NC-OE group was transfected with empty lentivirus (5E7TU). Initial experimental results showed that a multiplicity of infection of 50 was appropriate for both the PPARα-OE and NC-OE groups, with 5 μg/mL polybrene in the medium. HMCs were inoculated into 6-well plates (density, 1 × 10^6^ cells per well) for 24 h, then the indicated amounts of lentivirus and polybrene added to the medium; the medium containing lentivirus was replaced with fresh medium after 48 h. Subsequently, transfected HMCs in the two groups were stimulated with FBS and Gd-IgA1 for 24 h.

#### mRNA detection by real-time polymerase chain reaction

The mRNA levels of *PPARα*, *FABP1*, *GAPDH*, *GPX4*, and *ACSL4* in HMCs were analyzed by quantitative real-time polymerase chain reaction, using the following primers (Sangon Biotech, Shanghai, China): *GAPDH* forward, 5'-TGACATCAAGAAGGTGGTGAAGCAG-3' and reverse, 5'-GTGTCGCTGTTGAAGTCAGAGGAG-3'; *PPARα* forward, 5'-TGGCTGCTATCATTTGCTGTGGAG-3' and reverse, 5'-GAGAAAGATATCGTCCGGGTGGTTG-3'; *FABP1* forward, 5'-GTCCAAAGTGATCCAAAACGAA-3' and reverse, 5'-CGGTCACAGACTTGATGTTTTT-3'; *GPX4* forward, 5'-ATGGTTAACCTGGACAAGTACC-3' and reverse, 5'-GACGAGCTGAGTGTAGTTTACT-3'; *ACSL4* forward, 5'-ACCAGGGAAATCCTAAGTGAAG-3' and reverse, 5'-GGTGTTCTTTGGTTTTAGTCCC-3'.

#### Immunoblot analysis

PPARα, FABP1, GPX4, and ASCL4 protein levels were detected by western blotting. Protein samples were added to sodium dodecyl sulfate-polyacrylamide gels (8%-12%) separated by electrophoresis, then transferred to polyvinylidene fluoride membranes (0.45 μm), and membranes blocked in 5% skimmed milk. Then, membranes were incubated with the primary antibodies overnight at 4°C, followed by secondary antibodies for 1 h at room temperature. ImageJ software was used for optical density analysis, and densities of target proteins normalized to those of GAPDH.

#### Oxide detection

Levels of ROS, MDA, and GSH in HMCs were detected to assess oxide production. An MnSOD Assay Kit with WST-8 was used to evaluate mitochondrial ROS activity in HMCs, according to the manufacturer's instructions. To measure cellular GSH and MDA levels, HMCs were collected and assayed using GSH and MDA kits, according to the manufacturer's respective instructions.

#### Transmission electron microscopy

HMC specimens were preprocessed and analyzed for transmission electron microscopy (TEM) as previously described 33. Briefly, 1 mm^3^ of HMCs were collected, prefixed in 2% glutaraldehyde, and fixed in 1% osmium tetroxide. Next, samples were dehydrated in ethanol with 3% uranyl acetate, embedded in epoxy resin and propylene oxide overnight, and polymerized. After slicing into 70-nm-thick sections and staining with lead citrate, sections were detected using an H-7650 TEM (Hitachi H-7650). Two pathologists analyzed each section.

### Statistics

Data are presented as mean ± SD or mean ± SEM, and statistical analyses were conducted using Prism 8 software. Analysis of variance and the 2-tailed unpaired Student's t test were performed to assess differences between means, and P < 0.05 was considered statistically significant.

## Results

### Screening of DEGs from three datasets

**Figure 2:**
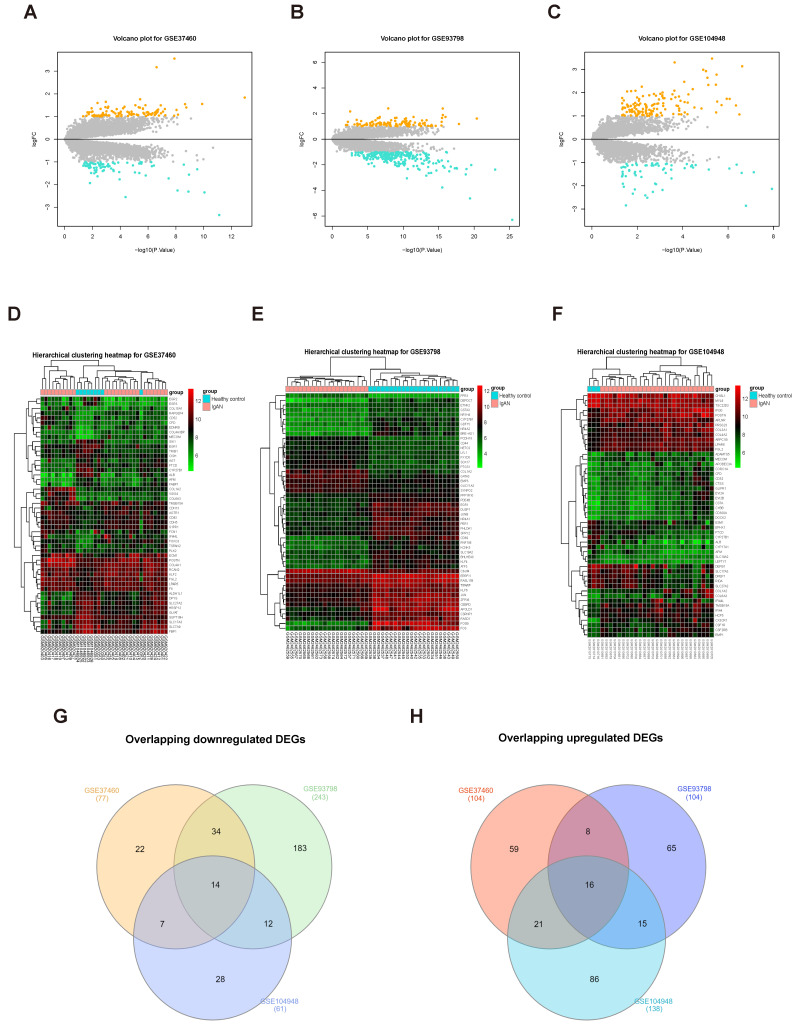
Screening DEGs from three datasets. Volcano plots of DEGs from GSE37460 (n = 181) (A), GSE93798 (n = 347) (B), and GSE104948 (n = 199) (C) are presented. Orange points represent upregulated genes, turquoise points represent downregulated genes, and grey points represent genes with no significant difference in expression (threshold, |fold-change| ≥ 1.0 and adjusted P < 0.05). (C, D, and F) Hierarchical cluster heatmaps of the top 50 DEGs from GSE37460 (C), GSE93798 (E), and GSE104948 (F) (threshold, |fold-change| ≥ 1.0 and adjusted P < 0.05). (G, H) Venn diagrams showing DEGs common to the three datasets; 14 downregulated (G) and 16 upregulated (H) DEGs were identified.

To screen for DEGs, we first normalized the original expression data from three datasets (Supplementary Fig. S1) and then compared gene expression levels in samples from patients with IgAN than those in healthy control groups. We detected 347 DEGs, including 243 upregulated and 104 downregulated genes, in the GSE93798 dataset (Supplementary Table S6); 181 DEGs were identified in the GSE37460 dataset, including 104 upregulated and 77 downregulated genes (Supplementary Table S7); and we identified 199 DEGs in GSE104948, including 138 upregulated and 61 downregulated genes (Supplementary Table S8). The expression levels of all DEGs in the three datasets are presented as volcano plots in Figure 2A-C, and the top 50 DEGs in the three datasets were subjected to hierarchical cluster analysis and the results illustrated as heatmaps (Fig. 2D-F). A search for DEGs common to the three datasets identified 16 upregulated and 14 downregulated DEGs (Fig. 2G-H and Table 1).

### Identification of hub genes using violin plot and ROC curve analysis

**Figure 3:**
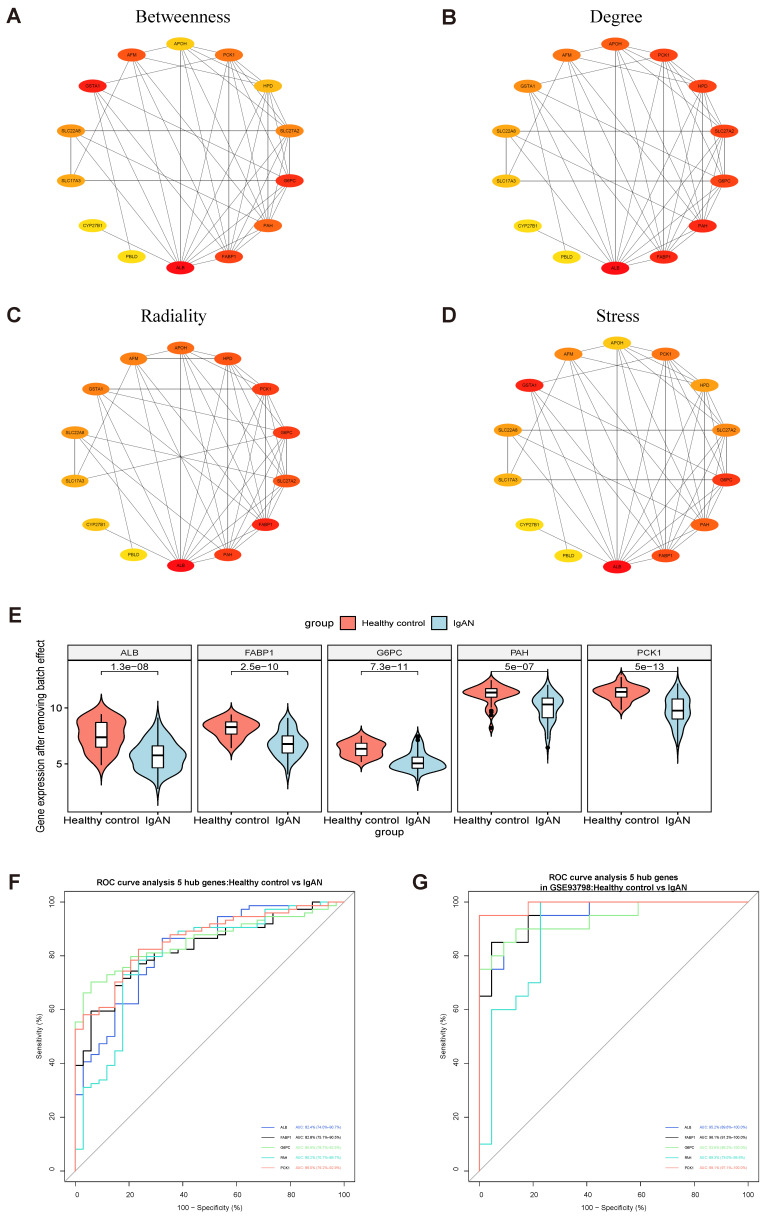
Identification of hub genes among overlapping downregulated DEGs. (A-D) 14 downregulated common DEGs were analyzed with the cytoHubba plugin of Cytoscape using four algorithms to assess betweenness (A), degree (B), radiality (C), and stress (D). Different colors represent different levels of connectivity with other genes in the PPI network. (E) Violin plot of initial expression after removal of batch effects for the top 5 key genes, showing that the 5 key genes were clearly decreased in patients with IgAN relative to healthy controls (P < 0.01). (F) ROC curve analysis of the top 5 key genes. AUC (95% CI), sensitivity, and specificity values for *ALB*, *FABP1*, *GP6C*, *PAH*, and *PCK1* were calculated by ROC curve analysis. (G) Verification of the 5 hub genes (*ALB*, *FABP1*, *GP6C*, *PAH*, and *PCK1*) by ROC curve analysis in the GSE93798 dataset.

PPI modules of the 14 downregulated DEGs were identified using the Stress, Betweenness, Radiality, and Degree algorithms in cytoHubba (Fig. 3A-D) (Supplementary Table S9). After removing batch effects of the original expression matrix on the five hub genes, a violin plot was generated illustrating overall differences in expression (Fig. 3E; Supplementary Fig. S2, Supplementary Table S10). To assess the sensitivity and specificity of the five hub genes for diagnosing IgAN, area under the ROC curve (AUC) values were calculated (Fig. 3F-G, Table 2). Further, we assessed the AUC values of the five hub genes in the GSE93798 dataset, with consistent results.

### Identification of key modules associated with clinical features by WGCNA

**Figure 4:**
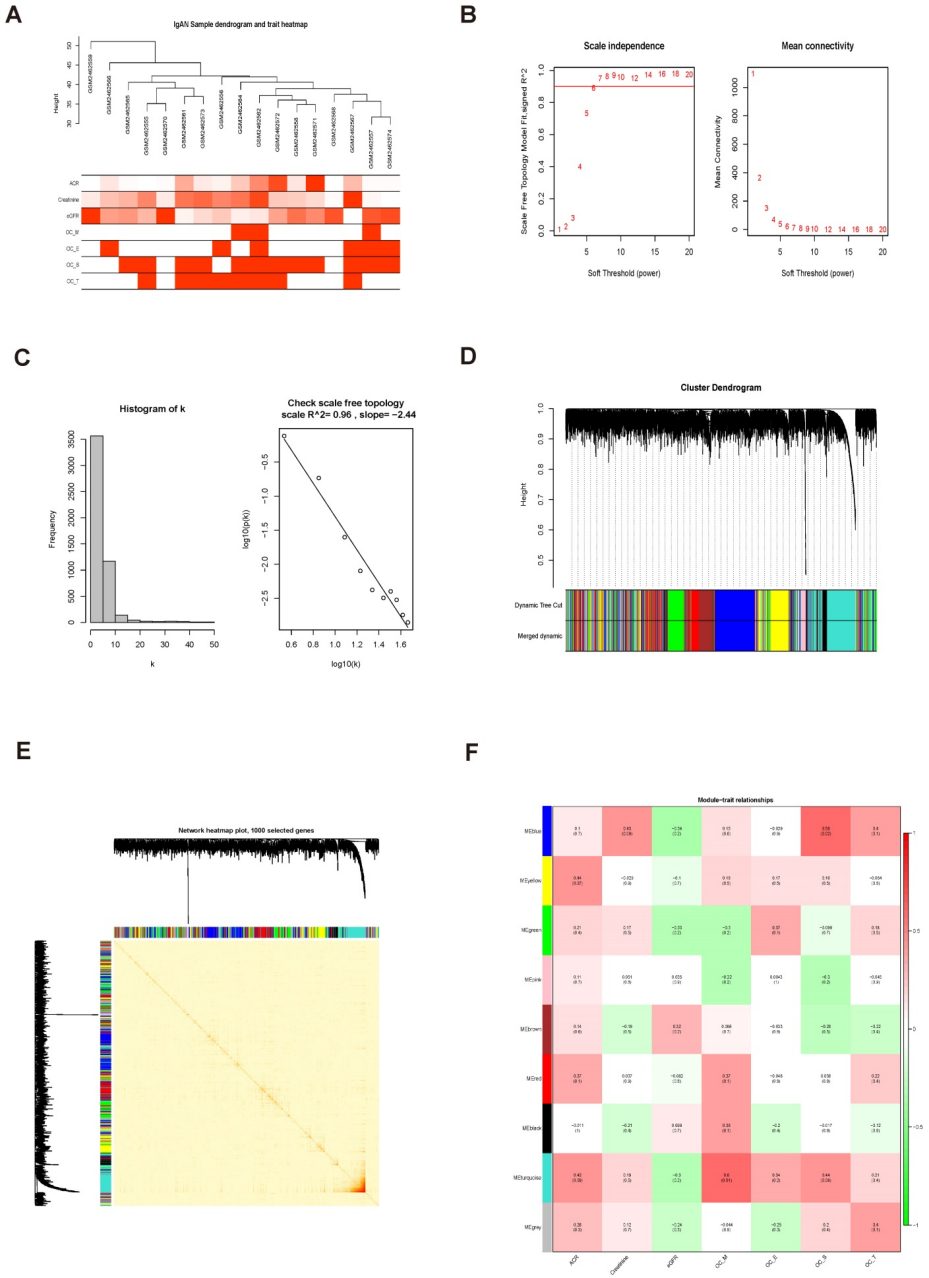
Identification of significant modules related to clinical traits by WGCNA. (A) Hierarchical clustering dendrogram of samples from the GSE93798 dataset. Clinical traits related to kidney function are displayed at the bottom of the plot. (B, C) Analysis of scale-free fit index and mean connectivity for various soft-threshold powers. The scale-free topology selected in our study was β = 9. (D) Hierarchical clustering dendrogram of different genes based on topological overlap. Modules were based on branches of the clustering tree. (E) Heatmap describing TOM among 1000 selected genes included in the WGCNA. Darker colors represent higher overlap and lighter colors correspond to lower overlap. Gene dendrogram and module assignment are shown on the left side and above. (F) Correlation between module eigengenes and clinical traits of IgAN. Each row corresponds to a module eigengene and columns represent clinical features. Each cell contains correlation and P values. The turquoise module was closely related to kidney function (ACR).

In this study, 17 samples and related clinical data were selected for WGCNA (Fig. 4A). After preliminary processing, 5000 genes associated with IgAN were screened for further analysis and a scale-free network generated using β = 9 (scale-free R^2^ = 0.96) as the soft-threshold (Fig. 4B, C). Based on the merged dynamic tree cut, we identified nine gene co-expression modules (Fig. 4D).

**Figure 5:**
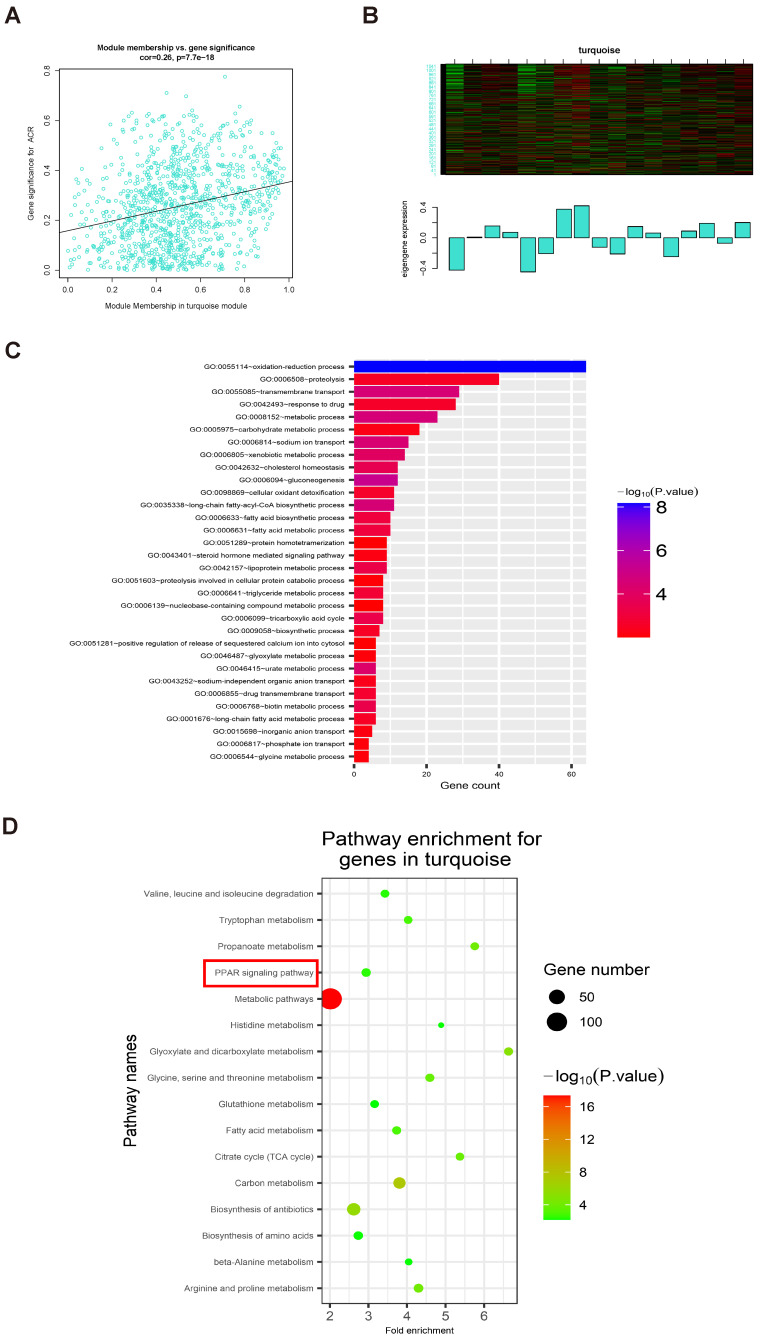
The relationship between clinical data and GO and KEGG enrichment analysis of genes in the turquoise module. (A) Scatterplots showing the relationships of GS and MM with ACR. The correlation coefficient between GS and ACR was 0.26 (P = 7.7e-18). *FABP1* had the highest GS and MM values for ACR (GS = 0.30 and MM = 0.76) relative to other genes in the turquoise module. (B) The expression distribution of 1060 genes in the turquoise module presented as a heatmap and bar graph. (C) GO enrichment analyses of genes in the turquoise module showing 32 significantly enriched Gene Ontology biological process terms (threshold, count ≥ 2 and P < 0.01). (D) KEGG pathways significantly enriched for the turquoise module, including PPAR signaling, are shown (threshold, count ≥ 2 and P < 0.01).

Analysis of 1000 selected genes demonstrated that each module verified others in the network TOM heatmap plot (Fig. 4E). Further, we found that a module colored turquoise showed the highest correlation with ACR, and contained FABP1 (R^2^ = 0.42, P = 0.09; Fig. 4F; Supplementary Fig. S3A); therefore, we considered this module significant. GS and MM values for genes in the turquoise module with clinical traits of patients with IgAN are presented as scatterplots; the correlation coefficient of GS value for ACR was 0.26 (P = 7.7e-18; Fig. 5A). Further, FABP1 had very high MM and GS values for ACR (MM = 0.76 and GS = 0.30) (Supplementary Table S11). More importantly, FABP1 has been identify patients at risk of developing kidney diseases, including AKI and CKD, and to protect the kidneys in the course of kidney disease, as detailed in our previously published review 14; hence, we considered FABP1 as a hub gene in the turquoise module. The expression of genes in the turquoise module is illustrated as a heatmap and bar graph (Fig. 5B).

### GO and KEGG enrichment analysis of genes in the turquoise module

GO enrichment analysis of genes in the turquoise module showed that they were mainly enriched for the following biological processes: oxidation-reduction process, proteolysis, transmembrane transport, response to drug, metabolic process, and carbohydrate metabolic process, among others (threshold, P < 0.01 and gene count ≥ 2) (Fig. 5C and Table 3). KEGG enrichment analyses of genes in the turquoise module revealed that they were primarily enriched in 16 KEGG pathways (Fig. 5D and Table 4) (threshold, P < 0.01 and count ≥ 2), including PPAR signaling, fatty acid metabolism, glutathione metabolism, and metabolic pathways, among others (Supplementary Fig. S4).

### Levels of PPARα, FABP1, and GPX4 were decreased in IgAN renal tissue

**Figure 6:**
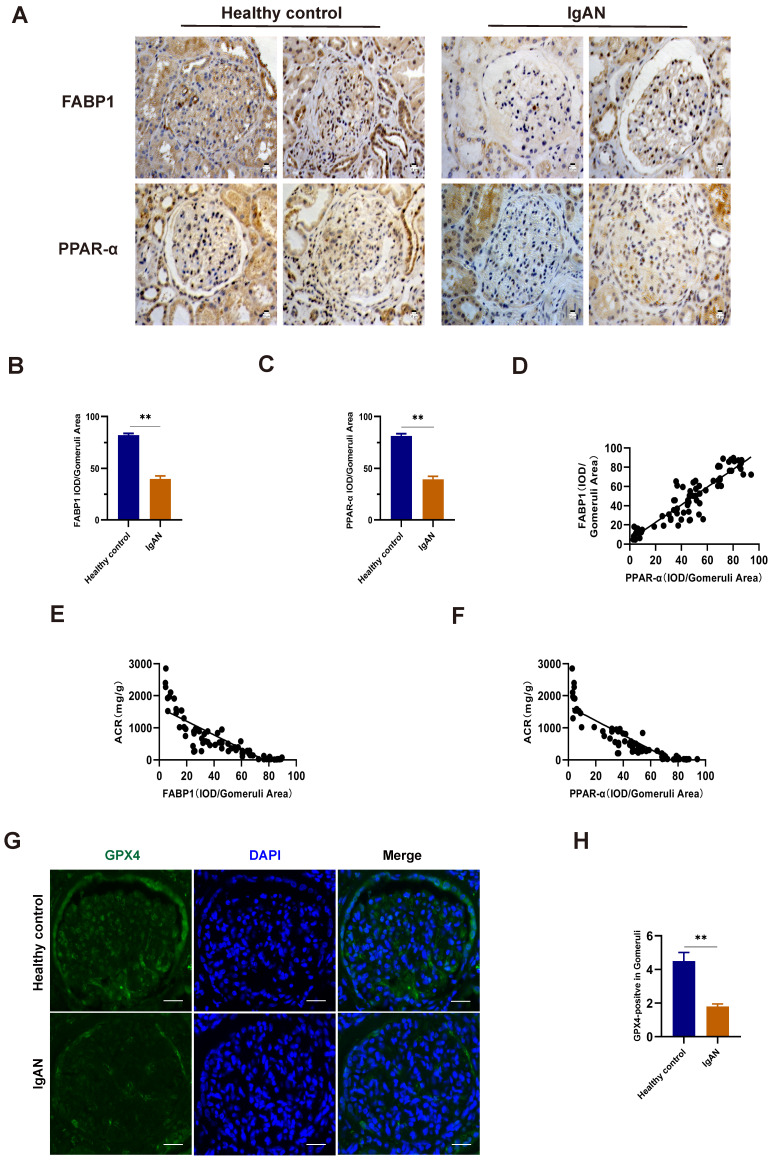
Levels of FABP1 and PPARα were decreased in IgAN renal tissues and closely related to ACR. (A-C) IHC and quantification of FABP1 and PPARα in human renal tissue. (D) Pearson correlation analysis of the relationship between FABP1 and PPARα expression. Decreased FABP1 expression levels were linearly positively correlated with those of PPARα (r = 0.9198, R^2^ = 0.8361, 95% CI: 0.8248-1.015). (G, H) Immunofluorescence detection of the ferroptosis marker, GPX4, in kidney tissue specimens; GPX4 protein levels were significantly decreased in IgAN tissues. Scale bar, 50 μm. Data are presented as mean ± SEM (patients with IgAN, n = 63 and healthy controls, n = 12). **P < 0.01.

To validate the findings of our bioinformatics analyses and explore their clinical relevance, we used renal tissue samples from patients with IgAN and healthy controls (Table 5). Compared with those in healthy controls, levels of FABP1 and PPARα were decreased in IgAN samples (Fig. 6A-C). Further, we found that the decrease in FABP1 expression level was linearly positively correlated with PPARα expression level (r = 0.9198, R^2^ = 0.8361, 95% CI: 0.8248-1.015), revealing that decreased PPARα protein levels lead to decreased FABP1 expression level (Fig. 6D). Moreover, FABP1 levels in IgAN renal tissue were negatively correlated with those of ACR (r = -0.8487, R^2^ = 0.7202, 95% CI: -0.9019 to -0.7700), and levels of PPARα in IgAN renal tissue were also negatively correlated with ACR levels (r = -0.8853, R^2^ = 0.7838, 95% CI: -0.9262 to -0.8239), indicating that kidney dysfunction in IgAN is associated with decreased levels of PPARα and FABP1 (Fig. 6E, F).

Immunofluorescence detection of the ferroptosis marker, GPX4, in kidney tissue specimens indicated that its protein levels were significantly lower in IgAN renal tissue than in healthy control samples (Fig. 6G, H), indicating that ferroptosis participates in the occurrence of IgAN.

### Gd-IgA1 stimulation of HMCs to induce ferroptosis

**Figure 7:**
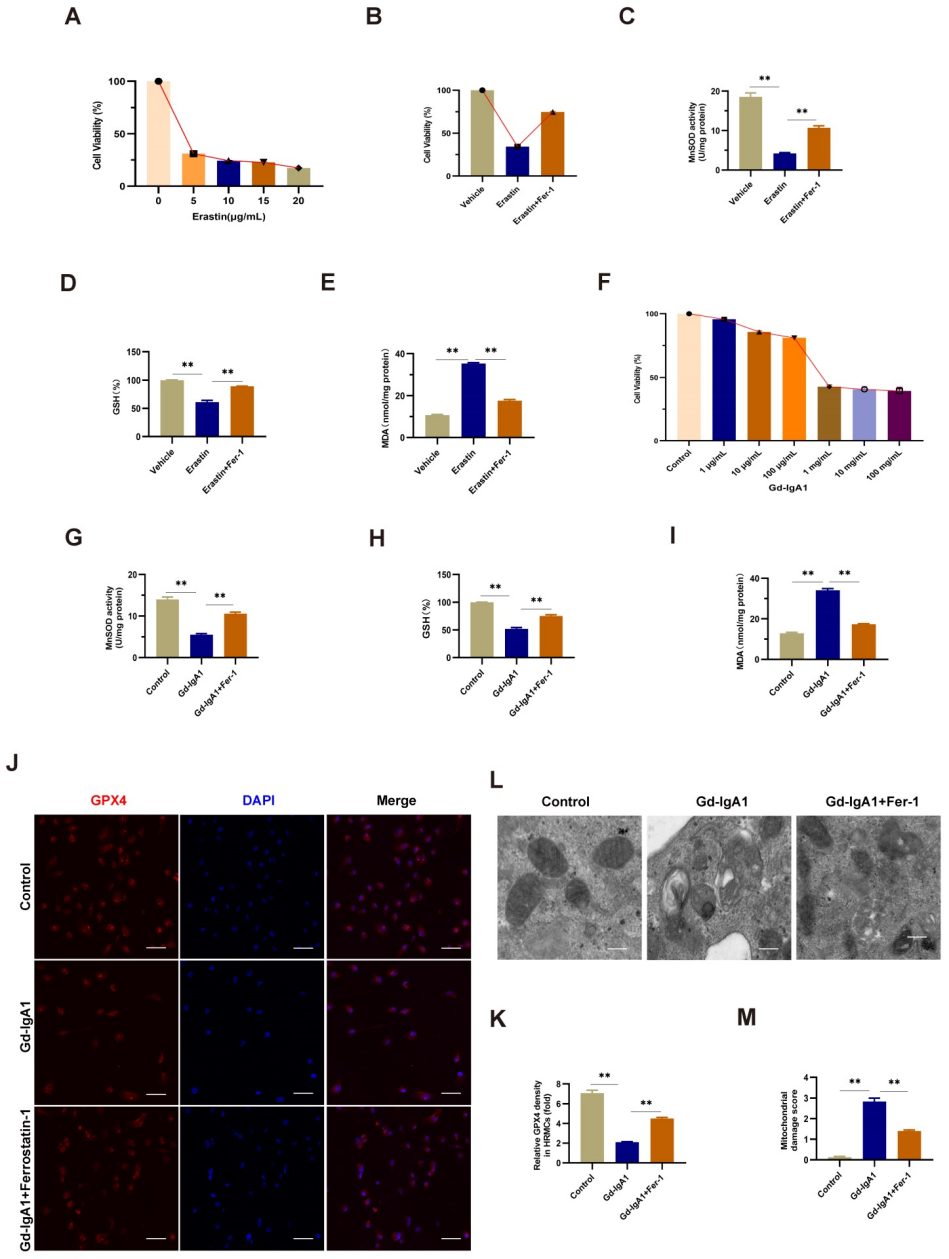
Gd-IgA1 leads to ferroptosis in HMCs. (A) Stimulation of HMCs with the ferroptosis inducer, erastin, significantly decreased cell viability at an erastin concentration of 5 μg/mL. HMC viability increased (B), levels of ROS and MDA decreased (C, E), and GSH levels increased (D) after intervention with the specific ferroptosis inhibitor, ferrostatin-1. (F) HMCs were exposed to different concentrations (1 μg/mL, 10 μg/mL, 100 μg/mL, 1 mg/mL, 10 mg/mL, and 100 mg/mL) of Gd-IgA1 for 24 h, and cell viability decreased significantly on treatment with 1 mg/ml Gd-IgA1. (G-I) After intervention with the ferroptosis specific inhibitor, ferrostatin-1, levels of ROS and MDA in HMCs were significantly decreased, and GSH levels were significantly increased, compared with the Gd-IgA1 group. (J, K) Expression levels of the ferroptosis marker, GPX4, were significantly decreased relative to the control group after HMCs were stimulated with Gd-IgA1. Scale bar, 50 μm. (L, M) TEM results showing that mitochondrial structure was significantly damaged in HMCs stimulated by Gd-IgA1. Mitochondrial damage score was significantly higher than that of the control group, while damage decreased following addition of ferrostatin-1. Scale bar, 1 μm. Data are presented as mean ± SEM (n = 3). **P < 0.01.

The ferroptosis inducer, erastin, was used to stimulate HMCs, and led to significantly decreased cell viability when applied at a concentration of 5 μg/mL (Fig. 7A). After intervention with the ferroptosis specific inhibitor, Fer-1, for 12 h prior to erastin treatment, HMC viability increased, levels of ROS and MDA decreased, and those of GSH increased (Fig. 7B-E), indicating the occurrence of ferroptosis in HMCs. Next, we used HMCs to establish an in vitro IgAN model by stimulating cells with different concentrations (1 μg/mL, 10 μg/mL, 100 μg/mL, 1 mg/mL, 10 mg/mL, and 100 mg/mL) of Gd-IgA1 for 24 h. We found that cell viability decreased significantly following treatment with 1 mg/mL Gd-IgA1 (Fig. 7F) and that addition of Fer-1 led to significantly decreased ROS and MDA levels (Fig. 7G, I), while those of GSH increased significantly (Fig. 7H), relative to the Gd-IgA1 group. Further, expression levels of the ferroptosis marker, GPX4, were significantly decreased relative to the control group after stimulation of HMCs with Gd-IgA1 (Fig. 7J, K).

TEM analysis showed that mitochondrial structure was significantly damaged in HMCs stimulated with Gd-IgA1, with mitochondrial damage scores significantly higher than those in control group cells, while damage was decreased after treatment with Fer-1 (Fig. 7L, M). These results suggest that Gd-IgA1 stimulation can cause ferroptosis in HMCs.

### Gd-IgA1 leads to ferroptosis in HMCs by inhibiting PPARα and FABP1 expression

**Figure 8:**
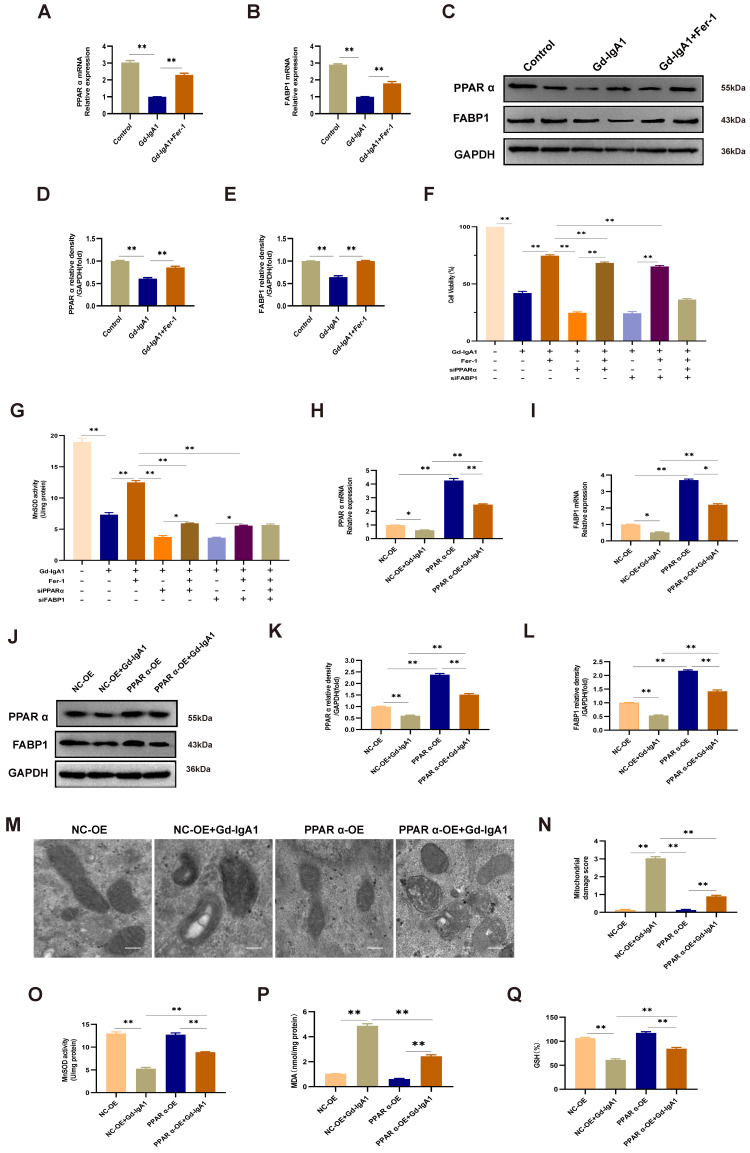
Gd-IgA1 leads to ferroptosis in HMCs by inhibiting PPARα and FABP1 expression. (A-E) Levels of PPARα and FABP1 were significantly decreased in HMCs stimulated by Gd-IgA1 (1 mg/mL, 24 h), and the expression of PPARα and FABP1 increased significantly after intervention with the ferroptosis-specific inhibitor, Fer-1. (F-G) After stimulation with Gd-IgA1 and then interference with siPPAR and siFABP1, HMC viability was significantly decreased and ROS significantly increased, while ferrostatin-1 treatment increased cell viability and reduced ROS levels. (H, I) After transfection of HMCs with PPARα lentivirus for 48 h, and stimulation with Gd-IgA1 for 24 h, *PPARα* and *FABP1* mRNA levels were significantly decreased. (J-L) PPARα and FABP1 protein levels were significantly decreased in HMCs transfected with PPARα lentivirus after Gd-IgA1 stimulation. (M, N) TEM results showing the relatively intact mitochondrial structure and reduced level of mitochondrial damage in HMCs after PPARα overexpression. (O-Q) Levels of ROS, GSH, and MDA oxide generation were determined to assess the occurrence of ferroptosis. After PPARα lentivirus overexpression in HMCs, ROS and MDA levels decreased and GSH levels increased. These results suggest that Gd-IgA1 leads to ferroptosis in HMCs by inhibiting PPARα and FABP1 expression. Scale bar, 1 μm. Data are presented as mean ± SEM (n = 3). **P < 0.01.

Levels of PPARα and FABP1 were significantly decreased in HMCs stimulated with Gd-IgA1 (1 mg/mL for 24 h) (Fig. 8A-E). After stimulation with Gd-IgA1 and treatment with siPPAR and siFABP1, HMC viability was significantly decreased and ROS was significantly increased, while Fer-1 increased HMC viability and reduced ROS levels (Fig. 8F, G). After transfection of HMCs with PPARα lentivirus for 48 h, and stimulation with Gd-IgA1 for 24 h, we observed that PPARα and FABP1 mRNA and protein levels were significantly decreased (Fig. 8H-L).

Further, TEM showed that mitochondrial structure was relatively intact in HMCs overexpressing PPARα, with reduced levels of mitochondrial damage (Fig. 8M, N). In addition, evaluation of ROS, GSH, and MDA oxide levels, which are related to the occurrence of ferroptosis, demonstrated that ROS and MDA levels were decreased (Fig. 8O, Q), while those of GSH were increased (Fig. 8P) after lentiviral overexpression of PPARα. These results suggest that Gd-IgA1 causes ferroptosis in HMCs by inhibiting PPARα and FABP1 expression.

### PPARα mediates FABP1 regulation of GPX4 and ACSL4, influencing the occurrence of ferroptosis in HMCs

**Figure 9:**
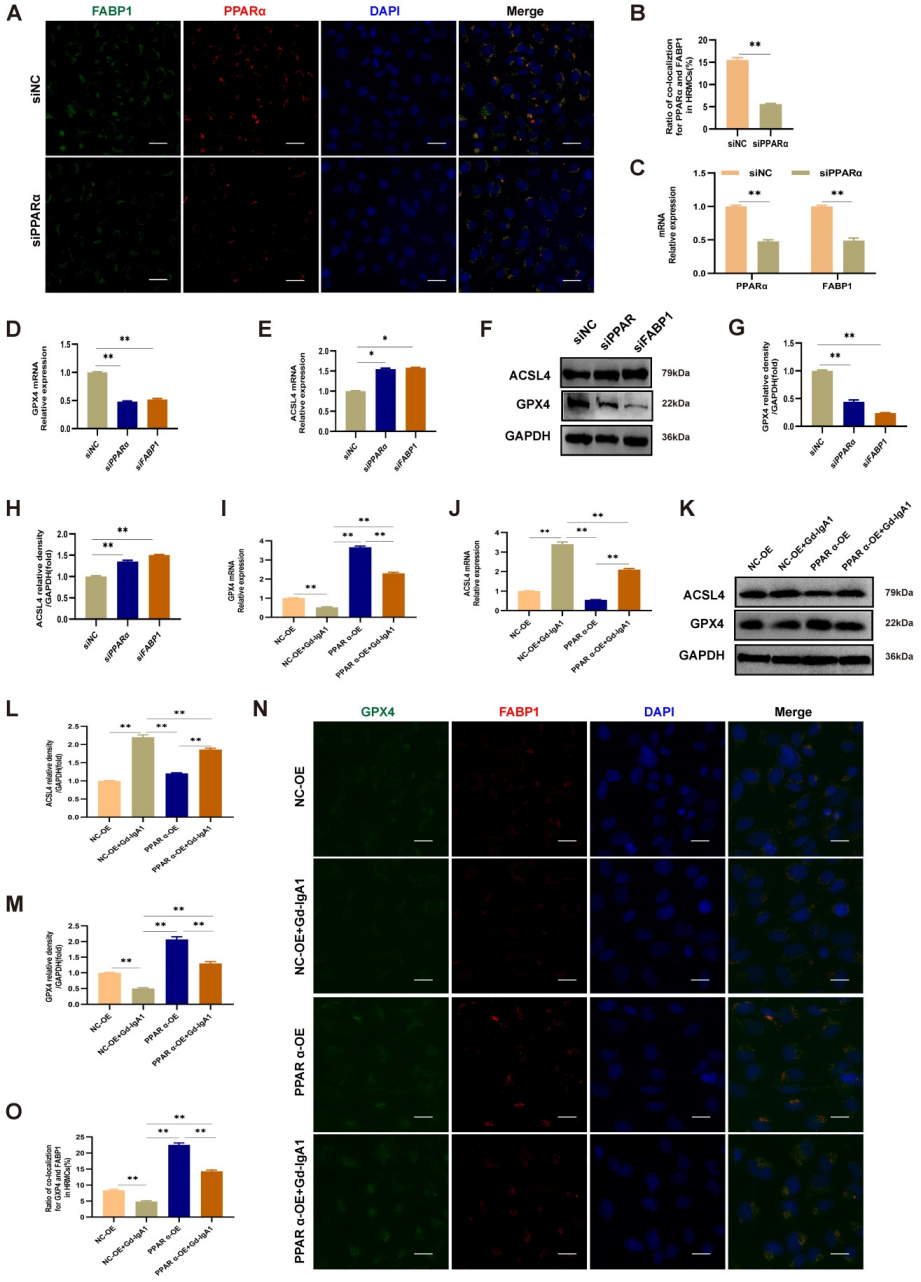
PPARα mediates FABP1 expression, regulating GPX4 and ACSL4, and influencing ferroptosis occurrence in HMCs. Immunofluorescence colocalization analysis of HMCs after intervention with siPPARα, showing that PPARα and FABP1 protein (A, B) and mRNA (C) levels were significantly decreased. These results indicate that downregulation PPARα mediated reduction of FABP1 levels. (D-H) mRNA and protein expression levels of the key ferroptosis molecule, GPX4, were decreased, and those of ACSL4 increased after treatment with siPPARα and siFABP1. After transfection of HMCs with PPARα lentivirus for 48 h, and stimulation with Gd-IgA1 for 24 h, *GPX4* mRNA level decreased (I) and *ACSL4* mRNA level was significantly increased (J). (K-M) GPX4 and ACSL4 protein levels were decreased and increased, respectively, in the PPARα-OE+Gd-IgA1 group. (N, O) Immunofluorescence colocalization analysis showing that FABP1 and GPX4 expression levels were increased after lentivirus transfection. GPX4 is a marker protein of ferroptosis inhibition, while ACSL4 promotes ferroptosis. These results indicate that PPARα and FABP1 regulate GPX4 and ACSL4, affecting ferroptosis occurrence in HMCs. Scale bar, 50 μm. Data are presented as mean ± SEM (n = 3). *P < 0.05, **P < 0.01.

After intervention with siPPARα, immunofluorescence colocalization analysis showed that PPARα and FABP1 expression levels were decreased (Fig. 9A, B), and *PPARα* and *FABP1* mRNA levels were significantly decreased (Fig. 9C). These results indicate that downregulation of PPARα mediated a decrease in FABP1 levels. To evaluate the effects of PPARα and FABP1 on ferroptosis, HMCs were pre-transfected with overexpression plasmids or siRNA for 48 h prior to Gd-IgA1 stimulation. The mRNA and protein expression levels of the key regulatory genes related to ferroptosis, GPX4 and ACSL4, were then detected, and co-expression of PPARα and GPX4 evaluated by immunofluorescence colocalization. The results showed that GPX4 expression levels were decreased, while those of ACSL4 increased, following treatment with siPPARα and siFABP1 (Fig. 9D-H). Further, on lentiviral overexpression of PPARα, GPX4 levels were increased and expression of ACSL4 decreased (Fig. 9I-M), while immunofluorescence colocalization analysis showed that PPARα and GPX4 expression levels were increased (Fig. 9N, O). GPX4 is a marker protein of ferroptosis inhibition, while ACSL4 promotes ferroptosis. Overall, our findings indicate that PPARα and FABP1 regulate GPX4 and ACSL4 to influence the occurrence of ferroptosis in HMCs.

## Discussion

In this study, we conducted comprehensive bioinformatics analysis of GEO datasets to identify genes and signaling pathways closely related to IgAN clinical traits. Although these datasets have been studied in previous research, we also conducted laboratory validation studies, using renal specimens from patients with IgAN, as well as in vitro experiments using HMCs. Preliminary screening of DEGs from the GSE93798, GSE104948, and GSE37460 datasets identified 14 common downregulated DEGs (Fig. 2G, Table 1). Four algorithms in the Cytoscape plugin, cytoHubba, were then applied to select five key genes for each algorithm, and we found that *FABP1* always ranked among the top 4 downregulated genes (Fig. 3A-D). Subsequent violin plot and ROC curve analyses confirmed identification of 5 key genes: *FABP1*, *PCK1*, *ALB*, *PAH*, and *G6PC* (Fig. 3F, G and Table 2). ALB is a major intravascular antioxidant that can protect kidneys 34. *PCK1* has been associated with type 2 diabetes mellitus, and its identification makes intuitive sense, as the enzyme it encodes, PEPCK-C, is a key gluconeogenic enzyme in liver and kidney 35. G6PC is a key enzyme involved in the maintenance of glucose homeostasis between meals, which catalyzes the hydrolysis of glucose-6- phosphate (G6P) to glucose and phosphate in the terminal step of gluconeogenesis and glycogenolysis 36, 37. PAH catalyzes the conversion of phenylalanine to tyrosine, and PAH deficiency causes hyperphenylalaninemia, which leads to severe mental retardation in the classical form of the disease, phenylketonuria (PKU)^38^, ^39^. A literature review regarding the ability of FABP1 to identify patients at risk of developing kidney diseases, including AKI and CKD, and to protect the kidneys in the course of kidney disease, has been published 14.

WGCNA of the GSE93798 dataset identified a turquoise module associated with ACR, with *FABP1* the gene in this module with the strongest correlation with ACR. We also found that the PPAR signaling pathway was associated with FABP1 regulation; therefore, we considered FABP1 as a hub gene and conducted further research into its function in this study. Fatty acid binding protein 1 (FABP1, or FABPL, L-FABP) is a 14.4 kDa protein expressed in human proximal tubules that regulates the cellular uptake, transport, and metabolism of fatty acids 13, 40. Importantly, FABP1 has a protective role against AKI and CKD during the course of kidney disease 14. PPARα is a transcription factor in the PPAR signaling pathway, and FABP1 is a downstream target gene in this pathway 11, 41. Peroxisome proliferator-activated receptor alpha (PPARα) is a member of the PPAR family, which functions as a nuclear transcription factor involved in fatty acid metabolism 10, 42. One study found PPARα levels are decreased in a UUO rat model 8, while another investigation showed damaged PPARα signaling in aging kidney during accelerated renal fibrosis 9. In our study, we analyzed renal tissue samples from patients with IgAN and healthy control subjects to explore the clinical relevance of our bioinformatics findings (Table 5). Levels of FABP1 and PPARα were lower in IgAN samples than those from healthy controls (Fig. 6A-C). Further, we found that decreased FABP1 expression levels were linearly positively correlated with those of PPARα, suggesting that decreased PPARα pathway protein expression levels lead to reduced FABP1 expression (Fig. 6D). Moreover, FABP1 and PPARα levels in renal tissues from patients with IgAN were negatively correlated with ACR level (Fig. 6E, F), indicating that reduction of PPARα and FABP1 in the kidney is involved in the dysfunction observed in IgAN.

Multiple molecular mechanisms are involved in the occurrence of IgAN, with the central mechanism underlying IgAN the accumulation of nephritogenic immune complexes in the glomerular mesangial area, leading to irreversible kidney damage in the form of segmental or global glomerular sclerosis and interstitial fibrosis 43. In this study, we used Gd-IgA1 extracted from the peripheral blood of patients with IgAN to stimulate HMCs and establish an in vitro IgAN model, and found that ferroptosis could be induced by treatment of HMCs with erastin. Further, our data show that Gd-IgA1 treatment clearly decreased HMC viability (Fig. 7F) and GSH levels, while significantly increasing ROS and MDA levels (Fig. 7G, I), and significantly decreasing the expression level of the ferroptosis marker, GPX4. When the specific inhibitor of ferroptosis, Fer-1, was added to HMCs before Gd-IgA1 stimulation, cell viability and GSH levels were increased, while ROS and MDA were decreased, relative to the Gd-IgA1-treated group. Further, TEM showed that mitochondrial structure was significantly damaged in HMCs stimulated by Gd-IgA1, with mitochondrial damage scores significantly higher than those of the control group (Fig. 7L-M), while Fer-1 reduced mitochondrial damage. Ferroptosis is involved in the occurrence of various kidney diseases, including AKI 18, renal fibrosis 19, polycystic kidney disease 20, and diabetic nephropathy 21; however, the effect of ferroptosis in IgAN was previously unclear. Here, we found that GPX4 protein levels were significantly reduced in renal tissue from patients with IgAN (Fig. 6G, H), indicating that ferroptosis may participate in the occurrence of IgAN. Our results indicate that Gd-IgA1 may cause ferroptosis of HMCs, contributing to IgAN.

In this study, we observed that FABP1 and PPARα levels were significantly decreased in IgAN renal tissue relative to those in healthy renal samples (Fig. 6A-C), and their levels were correlated with ACR (Fig. 6E, F). Increased PPARα activity can suppress ferroptosis, while decreased PPARα activity increased sensitivity to ferroptosis and, notably, overexpression of PPARα can suppress ferroptotic death 22. Further, there is evidence that, when FABP1 increases in cells, ROS production decreases 12. In our in vitro IgAN model, established by Gd-IgA1 stimulation of HMCs, we found that PPARα and FABP1 mRNA and protein expression levels were decreased, consistent with the results of our bioinformatics analyses (Fig. 8A-E). To explore the effect of PPARα and FABP1 expression levels on ferroptosis in HMCs, we intervened using small interfering RNA and lentivirus overexpression treatment, followed by stimulation with Gd-IgA1. Treatment with siPPARα and siFABP1 reduced cell viability and significantly increased ROS levels, while treatment with Fer-1 could correct the decreased cell viability and reduce ROS levels. These results indicate that decreased PPARα and FABP1 expression may be related to ferroptosis induced by Gd-IgA1 stimulation in HMCs. Further, on PPARα overexpression, ROS levels decreased (Fig. 8O), GSH levels increased (Fig. 8Q), and MDA level decreased (Fig. 8P), while TEM showed that mitochondrial structure of HMCs was relatively complete and clear (Fig. 8M), with mitochondrial damage levels reduced (Fig. 8N), suggesting that PPARα overexpression can decrease ferroptosis. Further, after intervention with siPPARα, immunofluorescence colocalization analysis showed that PPARα and FABP1 protein expression levels were decreased (Fig. 9A, B), while *PPARα* and *FABP1* mRNA levels were also significantly decreased (Fig. 9C). These results indicate that downregulation PPARα influences FABP1 levels. A previous study found that inhibition of FABP1 expression can improve NAFLD-related damage 44, indicating that FABP1 may play a protective role. In our study, we found that FBAP1 levels were decreased in IgAN tissues and HMCs after stimulation with Gd-IgA1, indicating that downregulation of FABP1 is related to the occurrence of IgAN. Based on the above results, we conclude that Gd-IgA1 leads to ferroptosis in HMCs by inhibiting PPARα-mediated FABP1 expression.

Ferroptosis can be triggered by a reduction or inhibition of GPX4 protein levels and GPX4 specifically scavenges lipid peroxides on membrane phospholipids, and is an essential negative regulator of ferroptosis 45, 46. Dixon 47 found that long-chain-fatty-acid-CoA ligase 4 (ACSL4) and lysophosphatidylcholine acyltransferase 3 have important roles in PUFA-containing phospholipid biosynthesis, which is key to the occurrence of ferroptosis. To explore the effects of PPARα and FABP1 on ferroptosis, HMCs were pre-transfected with overexpression plasmid or siRNA for 48 h prior to Gd-IgA1 stimulation, with siPPARα and siFABP1 treatment resulting in significantly decreased GPX4 levels and increased ACSL4 (Fig. 9D-H). Further, expression levels of the key ferroptosis gene, *GPX4*, were increased, while those of ACSL4 were decreased after PPARα overexpression in HMCs. Immunofluorescence colocalization analysis showed that FABP1 and GPX4 levels were increased after lentivirus transfection (Fig. 9N, O). In experiments involving folic acid-induced AKI, Martin-Sanchez 48 found that Fer-1 can specifically and significantly improve the renal function of mice, reduce intracellular lipid peroxidation, and reduce renal tubular epithelium cell damage and death. Further, levels of erastin-induced ferroptosis were significantly reduced after ACSL4 knockdown, while ACSL4 overexpression in ACSL4-negative cells promoted ferroptosis 49. Therefore, phospholipid peroxides produced through the ACSL4 pathway are considered executors of ferroptosis. Hence, we consider that downregulation of PPARα may decrease FABP1 and GPX4 expression levels, and increase those of ACSL4, leading ferroptosis.

## Conclusion

In summary, our comprehensive bioinformatics analysis screened out the hub gene, *FABP1*, and the PPAR signaling pathway as closely related to IgAN. Further, our experimental results indicate that downregulation of PPARα can mediate FABP1 regulation of GPX4 and ACSL4, contributing to IgAN, and leading to ferroptosis in an in vitro HMC model of IgAN. Thus, interfering with PPARα signaling to block ferroptosis is a potential target for preventing IgAN and delaying its progression.

## Supplementary Material

Supplementary figures and tables.

## Figures and Tables

**Figure 10:**
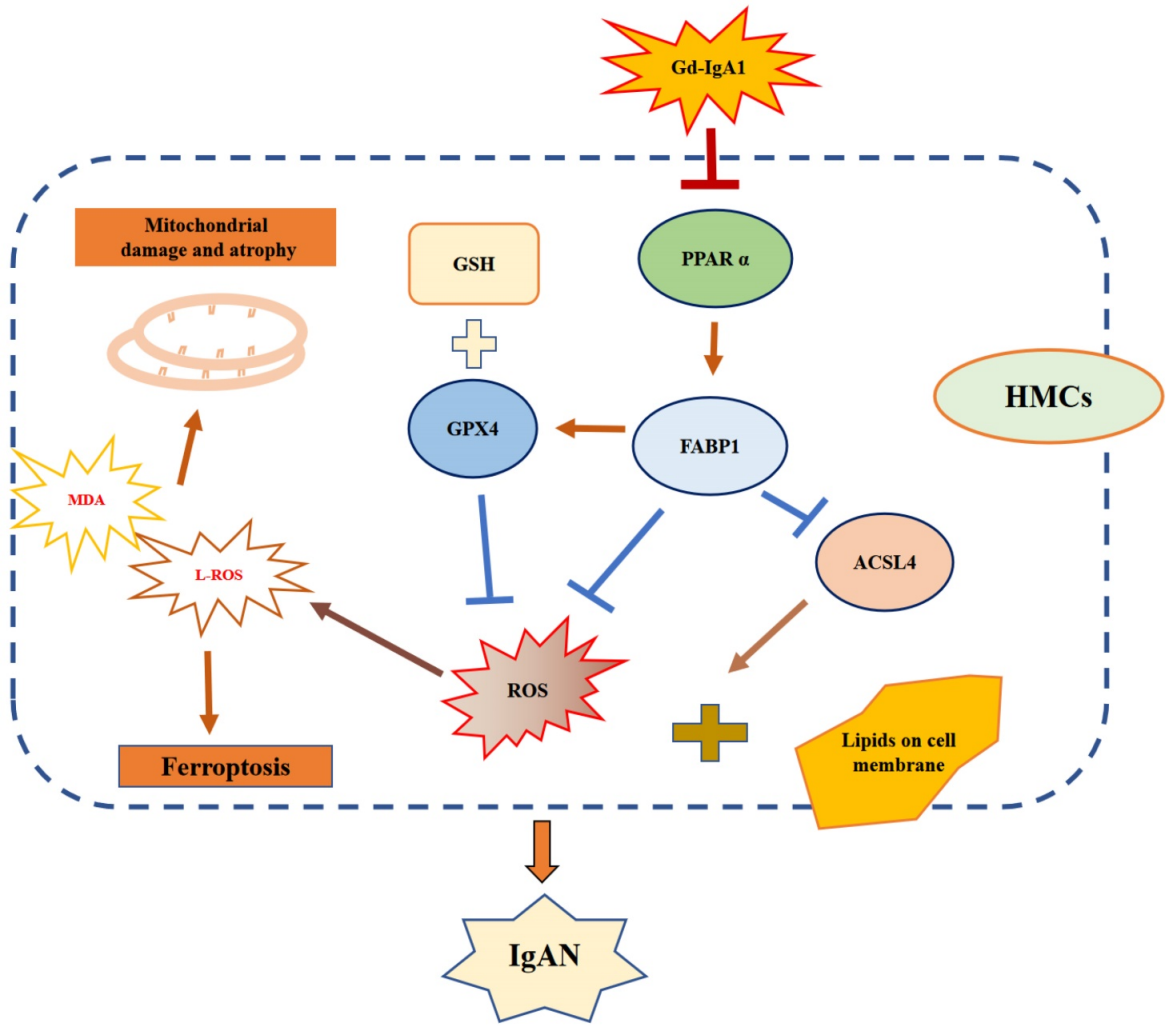
Schematic diagram showing regulation of ferroptosis by the PPARα signaling pathway in HMCs. Gd-IgA1 stimulates HMCs, leading to downregulated expression of PPARα, which in turn leads to decreased FABP1 expression. Levels of GPX4 and GSH, which participate in ferroptosis inhibition, decrease. ACSL4 promotion of ferroptosis and ROS levels increase, contributing to lipid peroxidation. Ultimately, ferroptosis of HMCs occurs, leading to IgAN.

**Table 1:** Overlapping DEGs in the GSE37460, GSE93798, and GSE104948 datasets

	Overlapping DEGs
**Upregulated (n = 16)**	*LPAR6*,* POSTN*,* COL1A2*,* COL6A3*,* MECOM*,* FN1*,* GATA3*,* HBB*,* HCLS1*,* HLX*,* IL10RA*,* C8orf4*,* TGFBI*,* C1QA*,* TYROBP*,* NETO2*
**Downregulated (n = 14)**	*AFM*,* ALB*,* APOH*,* CYP27B1*,* FABP1*,* G6PC*,* GSTA1*,* HPD*,* PAH*,* PBLD*,* PCK1*,* SLC17A3*,* SLC22A8*,* SLC27A2*

Note: DEGs were obtained using the limma package (threshold, |fold change| ≥ 1.0 and adjusted *p* < 0.05).

**Table 2:** ROC curve analysis of 5 hub genes for IgAN

Hub Genes	AUC	95% CI	P	Youden index	Sensitivity (%)	Specificity (%)
*ALB*	0.824	0.740-0.907	< 0.0001	0.5413	0.8649	0.6765
*FABP1*	0.828	0.751-0.905	< 0.0001	0.5421	0.6892	0.8529
*G6PC*	0.856	0.787-0.925	< 0.0001	0.6439	0.7027	0.9412
*PAH*	0.802	0.707-0.897	< 0.0001	0.5533	0.7297	0.8235
*PCK1*	0.860	0.792-0.929	< 0.0001	0.5890	0.8243	0.7647

AUC, area under the receiver-operating characteristic curve; CI, confidence interval.

**Table 3:** Biological processes enriched for genes in the turquoise module

ID	Description	Count	P
GO: 0055114	Oxidation-reduction process	64	6.62E-09
GO: 0006094	Gluconeogenesis	12	6.73E-06
GO: 0008152	Metabolic process	23	2.74E-05
GO: 0035338	Long-chain fatty-acyl-CoA biosynthetic process	11	2.81E-05
GO: 0055085	Transmembrane transport	29	2.86E-05
GO: 0006814	Sodium ion transport	15	3.77E-05
GO: 0046415	Urate metabolic process	6	5.90E-05
GO: 0006805	Xenobiotic metabolic process	14	1.04E-04
GO: 0042632	Cholesterol homeostasis	12	2.65E-04
GO: 0006768	Biotin metabolic process	6	3.97E-04
GO: 0006099	Tricarboxylic acid cycle	8	4.15E-04
GO: 0042157	Lipoprotein metabolic process	9	4.42E-04
GO: 0006631	Fatty acid metabolic process	10	8.99E-04
GO: 0006633	Fatty acid biosynthetic process	10	8.99E-04
GO: 0006641	Triglyceride metabolic process	8	0.001383392
GO: 0006855	Drug transmembrane transport	6	0.00187743
GO: 0098869	Cellular oxidant detoxification	11	0.002177808
GO: 0042493	Response to drug	28	0.002333424
GO: 0001676	Long-chain fatty acid metabolic process	6	0.003027159
GO: 0009058	Biosynthetic process	7	0.003030921
GO: 0006508	Proteolysis	40	0.003201312
GO: 0043252	Sodium-independent organic anion transport	6	0.004613136
GO: 0005975	Carbohydrate metabolic process	18	0.005595974
GO: 0043401	Steroid hormone mediated signaling pathway	9	0.006551124
GO: 0015698	Inorganic anion transport	5	0.006637744
GO: 0046487	Glyoxylate metabolic process	6	0.007981866
GO: 0006544	Glycine metabolic process	4	0.008023211
GO: 0006817	Phosphate ion transport	4	0.008023211
GO: 0051603	Proteolysis involved in cellular protein catabolic process	8	0.008707414
GO: 0051289	Protein homotetramerization	9	0.008917152
GO: 0051281	Positive regulation of release of sequestered calcium ion into cytosol	6	0.009406444
GO: 0006139	Nucleobase-containing compound metabolic process	8	0.009737803

**Note:** Significant GO enrichment terms of DEGs with *p* < 0.01 and count ≥ 2

**Table 4:** KEGG pathways enriched for genes in the turquoise module

ID	Description	Count	%	P
hsa01100	Metabolic pathways	137	13.01044634	4.45E-18
hsa01200	Carbon metabolism	24	2.279202279	4.14E-08
hsa01130	Biosynthesis of antibiotics	31	2.943969611	1.78E-06
hsa00630	Glyoxylate and dicarboxylate metabolism	10	0.949667616	8.95E-06
hsa00330	Arginine and proline metabolism	12	1.13960114	7.20E-05
hsa00640	Propanoate metabolism	9	0.854700855	9.95E-05
hsa00020	Citrate cycle (TCA cycle)	9	0.854700855	1.70E-04
hsa00260	Glycine, serine and threonine metabolism	10	0.949667616	2.23E-04
hsa01212	Fatty acid metabolism	10	0.949667616	1.13E-03
hsa00380	Tryptophan metabolism	9	0.854700855	1.37E-03
hsa03320	PPAR signaling pathway	11	1.044634378	3.65E-03
hsa00280	Valine, leucine and isoleucine degradation	9	0.854700855	3.98E-03
hsa01230	Biosynthesis of amino acids	11	1.044634378	0.006171739
hsa00340	Histidine metabolism	6	0.56980057	0.006245291
hsa00410	Beta-alanine metabolism	7	0.664767331	0.006432604
hsa00480	Glutathione metabolism	9	0.854700855	0.006642089

**Note:** Significant KEGG enrichment terms of DEGs with *p* < 0.01 and count ≥ 2

**Table 5:** Basic clinical data of patients with IgAN and healthy controls

		Healthy controls	IgAN
Sex	Male	7	35
	Female	5	28
Age (years)		58.0 ± 7.5	38.2 ± 11.8
Creatinine		48.3 ± 8.9	113.3 ± 74.0
eGFR		113.3 ± 12.7	77.3 ± 27.4
BUN		4.24 ± 0.58	5.77 ± 3.01
CysC		0.64 ± 0.18	1.26 ± 0.63
UA		327.4 ± 61.9	362.2 ± 92.3
24U-pro		139.7 ± 9.05	1649.8 ± 1487.5
ACR		21.73 ± 3.44	753.13 ± 662.59

IgAN, immunoglobulin A nephropathy; ACR, albumin-to-creatinine ratio; eGFR, estimated glomerular filtration rate; BUN, blood urea nitrogen; CysC, cystatin C; UA, uric acid; 24U-pro, 24 h urine protein. Patients with IgAN, n = 63; Healthy controls, n = 12. Data are presented as mean ± SD.
