# Supplementary material for: Downregulation of PPARα mediates FABP1 expression, contributing to IgA nephropathy by stimulating ferroptosis in human mesangial cells
Source: Int J Biol Sci. 2022 Aug 29;18(14):5438–58. doi: 10.7150/ijbs.74675 (PMC9461665; doi:10.7150/ijbs.74675)
Supplement: Supplementary file 1 — Supplementary figures and tables. [file ijbsv18p5438s1.pdf]

Supplementary Material

Supplementary Figure S1. Boxplots of original and normalized gene expression in GEO datasets. (A, C, and E) Boxplots of original gene expression in GSE93798 (A), GSE37460 (C), and GSE104948 (E). (B, D, and F) Boxplots of normalized gene expression in GSE93798 (B), GSE37460 (D), and GSE104948 (F).

Supplementary Figure S2. Violin plot of initial expression after removing batch effects of the 14 downregulated DEGs, indicating that these DEGs were clearly decreased in patients with IgAN relative to healthy controls ($P < 0.01$).

Supplementary Figure S3. Cluster trees and heatmaps for ACR in different modules and relationships between key modules and clinical features of IgAN in WGCNA. (A) Cluster trees and heatmaps for ACR. (B) Scatterplots of GS vs. MM in the turquoise module with creatinine and eGFR in patients with IgAN. Correlations between GS and creatinine and eGFR were –0.16(P = 1.6e−07) and 0.0088 (P = 0.77), respectively.

Supplementary Figure S4. GO analyses of genes in the turquoise module showing Molecular functions (A) and Cellular components (B) with threshold count ≥ 2 and $P < 0.01$.

Supplementary Table S1. Basic information of 23 IgAN-related datasets from the GEO database.

Supplementary Table S2. Basic information of the three GEO datasets analyzed in this study.

Supplementary Table S3. General clinical information of patients with IgAN in the GSE93798 dataset after exclusion of inappropriate samples.

Supplementary Table S4. General clinical information of 63 patients with IgAN included in this study.

Supplementary Table S5. General clinical information of 12 healthy control subjects included in this study.

Supplementary Table S6. All DEGs identified in the GSE93798 dataset.

Supplementary Table S7. All DEGs identified in the GSE37460 dataset.

Supplementary Table S8. All DEGs identified in the GSE104948 dataset.

Supplementary Table S9. Top 5 common downregulated DEGs identified using the Degree, Radiality, Stress, and Betweenness algorithms.

Supplementary Table S10. Gene expression matrix of the 5 hub genes after removal of batch effects.

Supplementary Table S11. GS and MM values with IgAN clinical traits in the turquoise module.

Figue S1

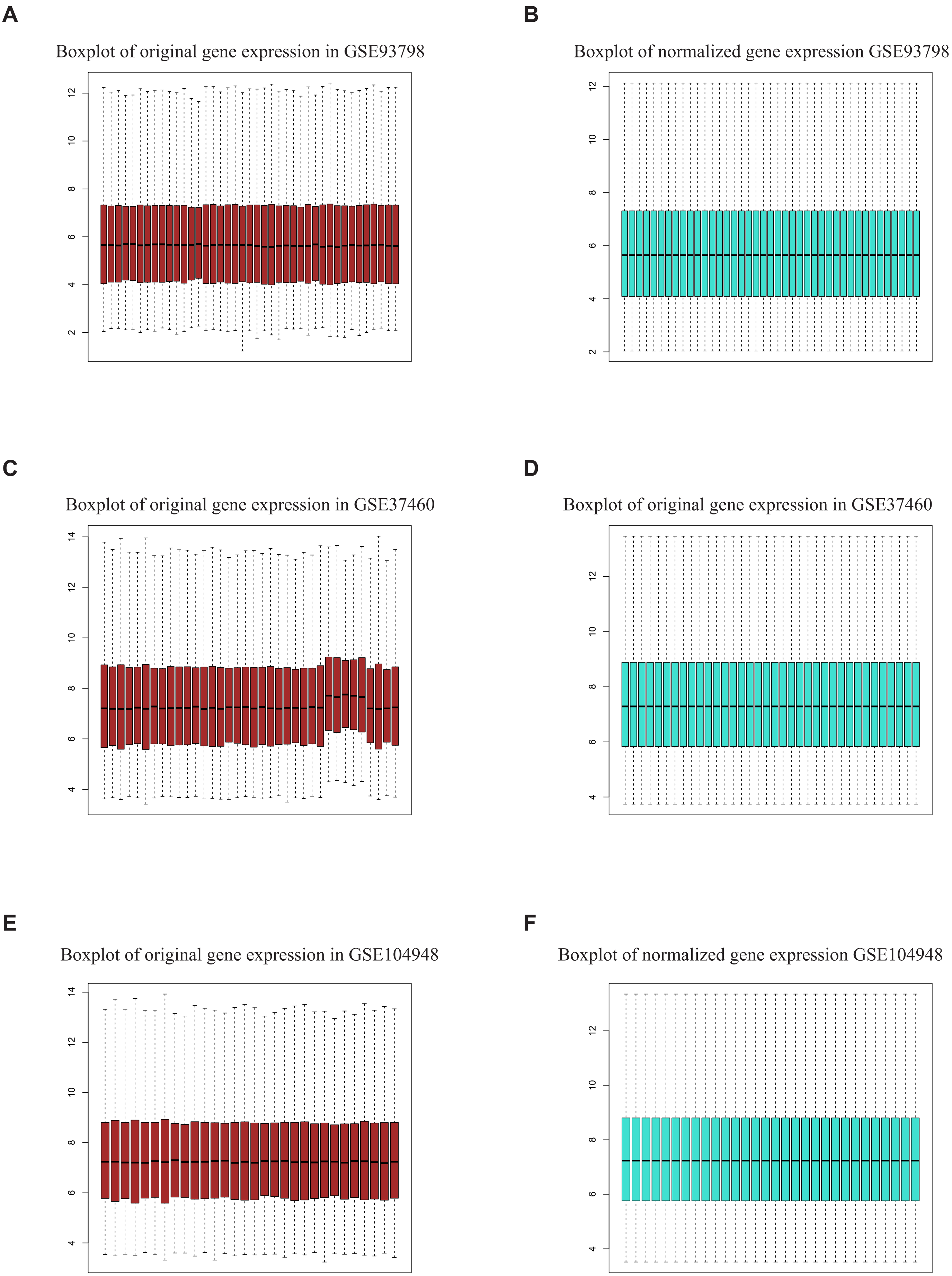
A
Boxplot of original gene expression in GSE93798
B
Boxplot of normalized gene expression GSE93798
C
Boxplot of original gene expression in GSE37460
D
Boxplot of original gene expression in GSE37460
E
Boxplot of original gene expression in GSE104948
F
Boxplot of normalized gene expression GSE104948

Figue S2

A

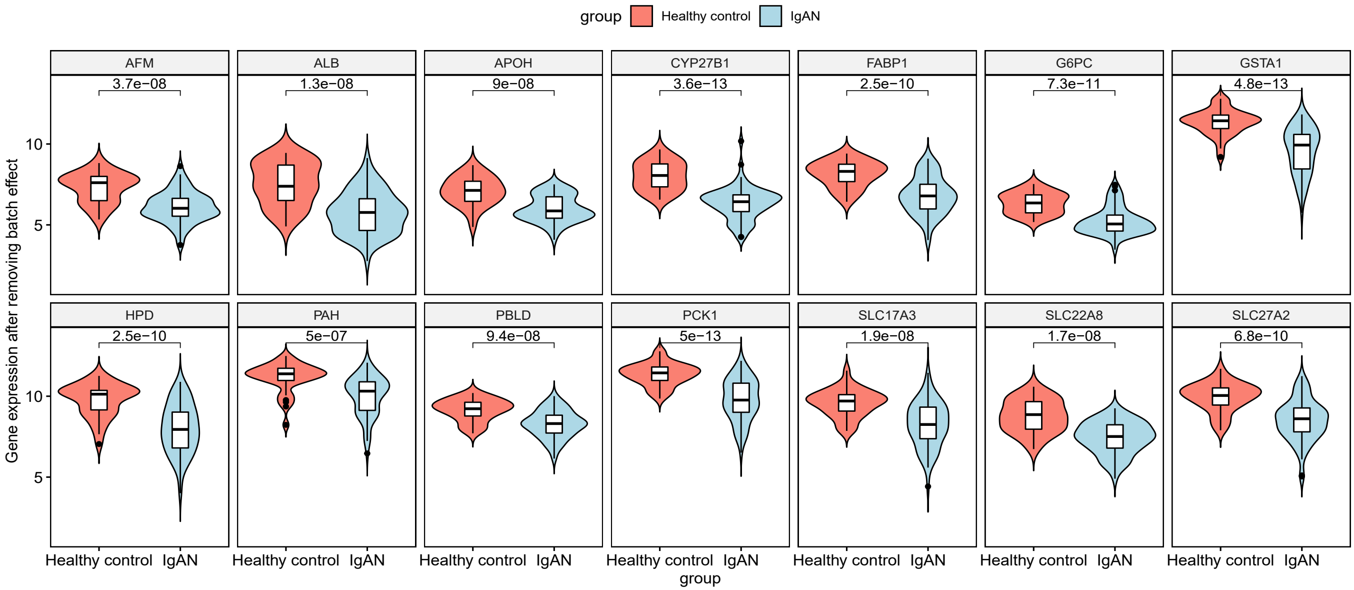

group
Healthy control
IgAN
AFM
3.7e−08
ALB
1.3e−08
APOH
9e−08
CYP27B1
3.6e−13
FABP1
2.5e−10
G6PC
7.3e−11
GSTA1
4.8e−13
HPD
2.5e−10
PAH
5e−07
PBLD
9.4e−08
PCK1
5e−13
SLC17A3
1.9e−08
SLC22A8
1.7e−08
SLC27A2
6.8e−10
Gene expression after removing batch effect
10
5
Healthy control
IgAN
group

Figue S3

A

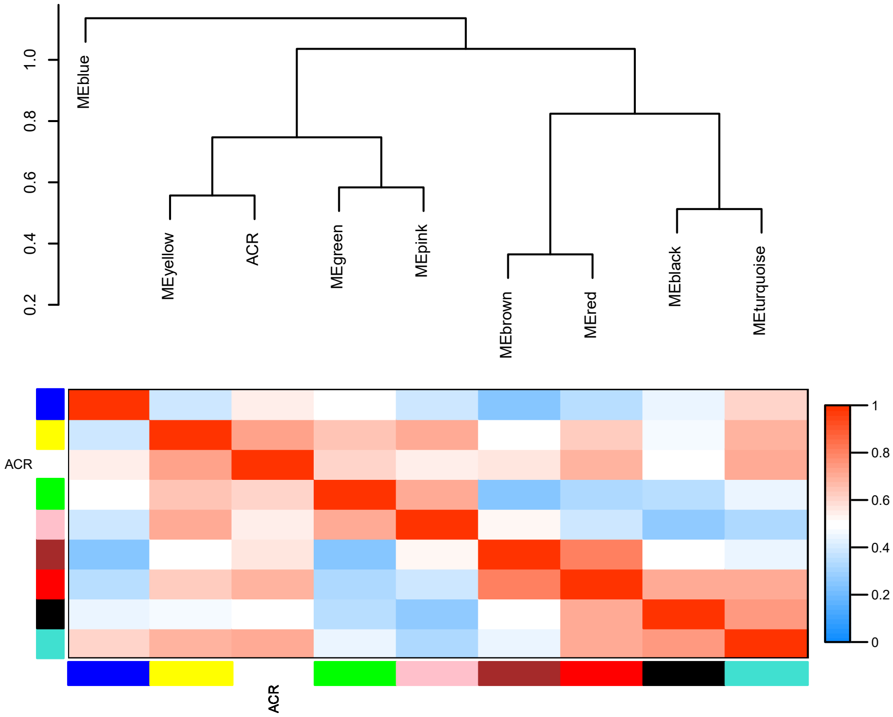
1.0
0.8
0.6
0.4
0.2
MEblue
MEyellow
ACR
MEgreen
MEpink
MEbrown
MEred
MEblack
MEturquoise
ACR
1
0.8
0.6
0.4
0.2
0
ACR

B

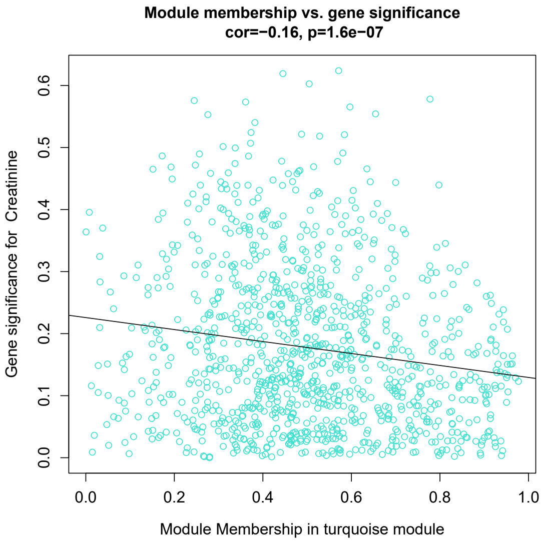
Module membership vs. gene significance
cor=−0.16, p=1.6e−07
Gene significance for Creatinine
Module Membership in turquoise module
0.0
0.1
0.2
0.3
0.4
0.5
0.6
0.0
0.2
0.4
0.6
0.8
1.0

C

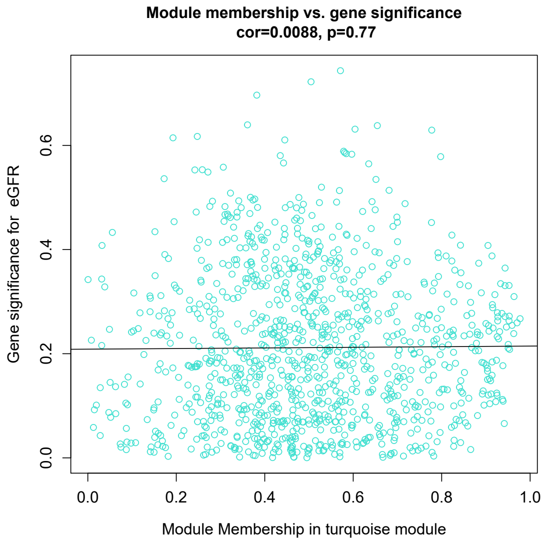
Module membership vs. gene significance
cor=0.0088, p=0.77
Gene significance for eGFR
Module Membership in turquoise module
0.0
0.2
0.4
0.6
0.0
0.2
0.4
0.6
0.8
1.0

Figue S4

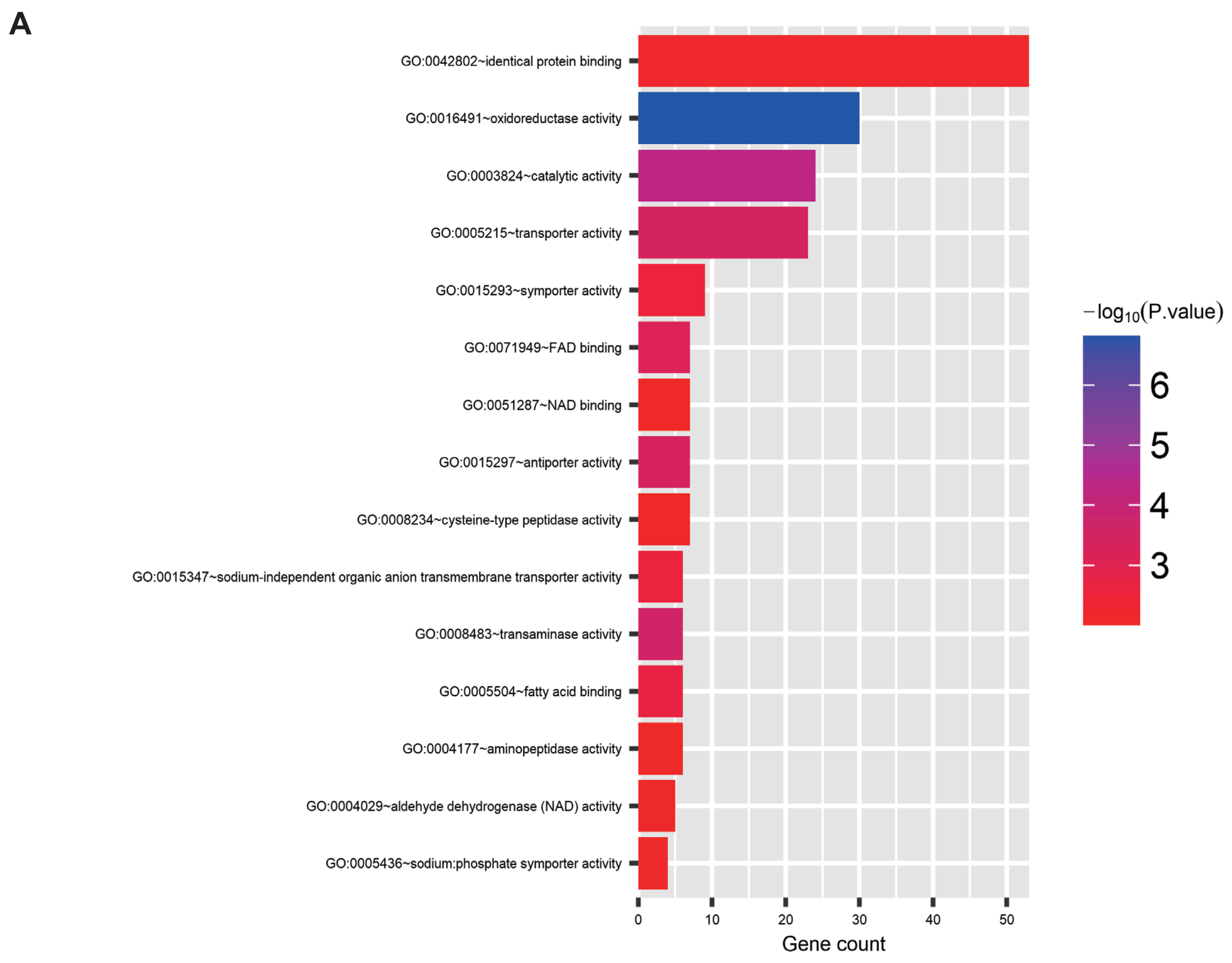
A
GO:0042802~identical protein binding
GO:0016491~oxidoreductase activity
GO:0003824~catalytic activity
GO:0005215~transporter activity
GO:0015293~symporter activity
GO:0071949~FAD binding
GO:0051287~NAD binding
GO:0015297~antiporter activity
GO:0008234~cysteine-type peptidase activity
GO:0015347~sodium-independent organic anion transmembrane transporter activity
GO:0008483~transaminase activity
GO:0005504~fatty acid binding
GO:0004177~aminopeptidase activity
GO:0004029~aldehyde dehydrogenase (NAD) activity
GO:0005436~sodium:phosphate symporter activity
0
10
20
30
40
50
Gene count
$-\log_{10}$(P.value)
6
5
4
3

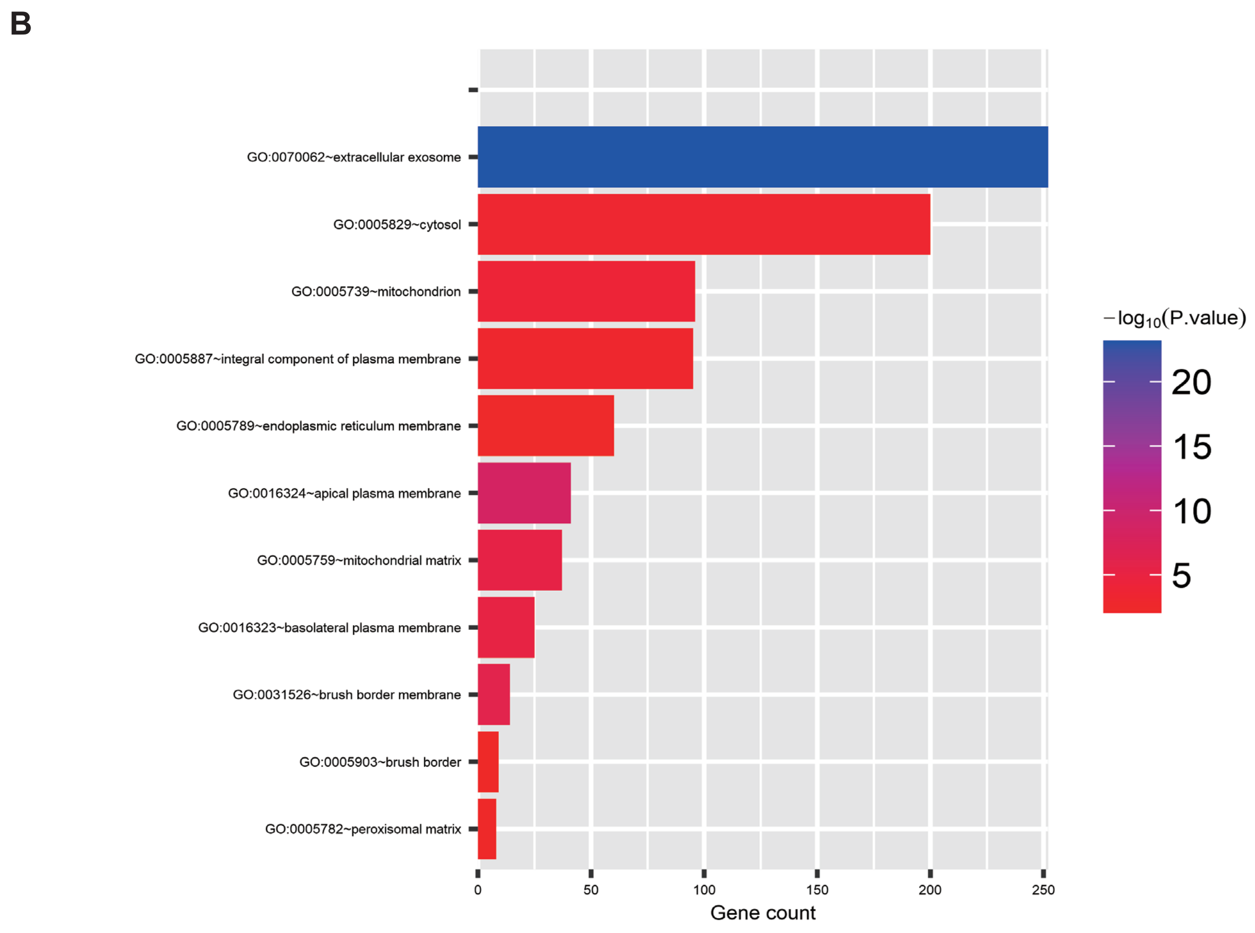
B
GO:0070062~extracellular exosome
GO:0005829~cytosol
GO:0005739~mitochondrion
GO:0005887~integral component of plasma membrane
GO:0005789~endoplasmic reticulum membrane
GO:0016324~apical plasma membrane
GO:0005759~mitochondrial matrix
GO:0016323~basolateral plasma membrane
GO:0031526~brush border membrane
GO:0005903~brush border
GO:0005782~peroxisomal matrix
0
50
100
150
200
250
Gene count
$-\log_{10}$(P.value)
20
15
10
5

Table S1 Basic information of 23 IgAN-related datasets from the GEO database

Serial number	Platform	IgAN	Control	Study type	Tissue	Notes
GSE115857	GPL14951	55	7	Expression profiling by array	Renal tissue	
GSE104954	GPL24120	25	21	Expression profiling by array	Tubulointerstitium	
GSE104948	GPL24120	27	3	Expression profiling by array	Glomeruli	
GSE99340	GPL19184	50	0	Expression profiling by array	Glomeruli	
GSE99339	GPL19184	26	0	Expression profiling by array	Glomeruli	
GSE99325	GPL19184	24	0	Expression profiling by array	Tubulointerstitium	
GSE93798	GPL22945	20	22	Expression profiling by array	Glomeruli	
GSE73953	GPL4133	15	16	Expression profiling by array	Peripheral blood mononuclear cells (PBMCs)	
GSE60861	GPL13497	4	0	Expression profiling by array<br>Non-coding RNA profiling by array	Kidney Biopsy	
GSE60860	GPL13497	2	0	Expression profiling by array<br>Non-coding RNA profiling by array	Kidney Biopsy	
GSE45980	GPL13497	2	0	Expression profiling by array<br>Non-coding RNA profiling by array	Kidney Biopsy	
GSE69438	GPL11670	2	0	Expression profiling by array	Tubulointerstitium	
GSE50469	GPL17662	22	0	Expression profiling by array	Glomeruli	

GSE58539	GPL10558	8	9	Expression profiling by array	Peripheral blood mononuclear cells (PBMCs)	
GSE47185	GPL14663	1	0	Expression profiling by array	Tubulointerstitium	
GSE47184	GPL14663	1	0	Expression profiling by array	Tubulointerstitium	
GSE37463	GPL14663	27	9	Expression profiling by array	Glomeruli	This dataset contains 2 superseries of GSE37455 and GSE37460, and IgAN- related dataset is only in GSE37460.
GSE37460	GPL14663	27	9	Expression profiling by array	Glomeruli	
GSE27676	GPL96	6	0	Expression profiling by array	Peripheral blood mononuclear cells (PBMCs)	
GSE35489	GPL14663/ GPL96	50	12	Expression profiling by array	Tubulointerstitium	
GSE35488	GPL14663	25	6	Expression profiling by array	Tubulointerstitium	
GSE35487	GPL96	25	6	Expression profiling by array	Tubulointerstitium	
GSE14195	GPL96	12	8	Expression profiling by array	Peripheral blood mononuclear cells (PBMCs)	

Table S2 Basic information of the three GEO datasets analyzed in this study

Serial number	Tissue	Platform	IgAN	Healthy control
GSE37460	Glomeruli	GPL14663	27	9
GSE104948	Glomeruli	GPL24120	27	3
GSE93798	Glomeruli	GPL22945	20	22

Note: The specimens in these datasets are derived from the kidney tissues of IgAN and Healthy control.

Table S3 General clinical information of patients with IgAN in the GSE93798 dataset after exclusion of inappropriate samples

Sample	Sex	Age,yr	Urine Albumin-to-Creatinine Ratio	Creatinine,μmol/L	eGFR	CKD Stage	BP	BP Medication	Steroids	Immuno suppressive Therapy	Oxford Classification				Progression
											M	E	S	T	
GSM2462561	Female	54	229	132	39.4	3	130/80	Yes	No	No	0	0	1	1	-4.55
GSM2462562	Male	46	323	155	45.6	3	150/90	Yes	No	No	1	1	1	1	0.6
GSM2462564	Female	45	155	132	40.5	3	140/80	Yes	No	No	1	0	1	1	-3.05
GSM2462567	Female	41	189	165	33	3	130/80	Yes	No	No	0	1	1	1	-5.37
GSM2462556	Male	49	37	124	58.4	3	125/80	No	No	No	0	1	0	1	-3.86
GSM2462573	Male	48	65	139	51.3	3	125/85	No	No	No	0	0	1	1	-18.99
GSM2462560	Male	58	70	107	65.6	2	140/80	Yes	No	No	0	0	1	0	-8.49
GSM2462565	Male	35	14	103	80.7	2	120/80	Yes	No	No	0	0	1	0	2.40
GSM2462563	Male	58	61	117	67.9	2	130/90	Yes	No	No	0	0	1	2	-0.84
GSM2462555	Male	34	5	124	64.9	2	126/85	Yes	No	No	0	0	1	1	1.18
GSM2462566	Male	53	74	96	77.4	2	110/70	No	Yes	No	0	1	0	0	-27.74
GSM2462571	Male	61	560	84	86	2	150/95	Yes	No	No	0	0	1	0	-3.94
GSM2462572	Male	65	490	93	74	2	155/95	Yes	No	No	0	0	1	1	-1.88
GSM2462557	Female	17	15	71	108.2	1	130/80	No	No	No	1	1	1	0	-2.24
GSM2462558	Male	30	75	91	97.1	1	130/90	Yes	No	No	0	0	1	0	4.49
GSM2462559	Male	20	1.2	76	124.8	1	100/60	No	No	No	0	0	0	0	-6.94
GSM2462568	Female	23	0.6	68	109.3	1	110/65	No	No	No	0	0	0	0	3.92
GSM2462570	Male	24	12	77	125.6	1	120/70	No	No	No	0	0	0	0	-17.45
GSM2462574	Female	23	13	66	113.3	1	120/70	No	No	No	0	1	1	0	3.77

Table S4 General clinical information of 63 patients with IgAN included in this study

Case number	Gender	Age	Serum creatinine(μmol/L)	eGFR	BUN（mmol/L）	Cysc(mg/L)	UA(μmol/L)	24U-pro(mg/24h)	ACR(mg/g)	M	E	S	T	C	Lee classification
1	Female	52	158.5	32	6.1	2.05	338	816	892.1	1	0	1	1	1	IV
2	Male	28	154	52	7.3	1.46	472	3931.4	662.3	1	0	1	1	1	IV
3	Male	43	136	55	7.05	1.41	456	2765.3	1453.6	1	0	1	1	1	IV
4	Female	42	97	62	3.1	1.14	342	3632	1920.9	1	0	1	1	1	IV
5	Male	31	103	83	4.9	1.11	312	1251	575.6	1	0	1	1	1	IV
6	Female	30	83	82	5.4	1.04	383	1282.6	356.2	1	0	1	1	1	IV
7	Male	31	99	87	6.1	0.89	435	205.2	139	1	0	1	0	0	IV
8	Female	30	74	94	6.2	1.11	380	2081.7	552.3	0	0	1	0	0	IV
9	Male	23	74	124	3	0.75	283	228.8	107.5	1	0	1	0	0	III
10	Female	37	44	124	3.5	0.63	238	1178	2271.7	0	0	1	0	1	III
11	Male	24	352.8	20	8	2.63	540	3353	458.1	1	0	1	2	0	V
12	Male	42	110.2	71	6	1.03	458	555	122.7	1	0	1	0	0	III
13	Male	28	89	101	4	0.99	351	840	273.3	1	0	1	0	1	III-IV
14	Female	64	79	69	4.9	1.04	320	1890	1015.7	0	0	0	0	0	III-IV
15	Male	27	80	116	4.4	1.02	226	451	146.6	1	0	1	1	1	IV
16	Female	50	63	99	3.1	0.90	296	441	146.7	0	0	0	0	0	III
17	Male	44	104	75	4.8	1.58	372	3741.4	525.6	1	1	1	0	0	III-IV
18	Female	27	71	101	5	0.89	309	1102.5	1969.3	1	1	1	0	1	IV
19	Male	26	77	119	3.7	1.27	477	357	252.9	1	0	0	0	0	III-IV
20	Male	20	445	15	21.4	4.57	307	3268.4	2854.7	1	1	1	2	1	V

21	Male	52	294	20	17.5	2.84	539	5022	1584.1	1	0	1	2	1	V
22	Male	50	79.1	99	3.6	1.03	297	306.6	281.2	1	0	1	1	0	IV
23	Male	33	96	89	5.1	1.17	396	2541.5	465.5	1	0	1	1	1	IV
24	Female	36	96	66	4.8	1.07	318	538.5	270	1	0	1	0	0	IV
25	Male	20	98	91	5.6	1.02	418	1110	741.6	1	0	1	0	1	IV
26	Male	55	96.3	76	7.2	1.23	493	944	430.3	1	1	0	1	0	IV
27	Female	32	60	116	4.3	0.94	267	552	199.4	0	0	0	0	1	III
28	Male	35	140	56	6.5	1.43	313	1304.8	946.1	1	0	1	1	0	IV
29	Male	31	81.3	111	4.9	0.93	336	368	73.7	0	0	1	1	1	IV
30	Female	41	49	117	3.6	0.65	255	568.4	359.5	0	0	0	0	0	III
31	Male	38	382	16	13.8	3.16	517	3914	1536.9	1	0	1	2	0	V
32	Female	71	47.1	95	5.3	1.05	246	3271.2	1293.7	1	0	1	0	1	III
33	Male	41	131	58	5.8	1.39	527	7657.6	1535.8	1	0	1	1	1	IV
34	Female	49	100	57	5.8	0.93	209	681.6	303.2	1	0	1	1	0	IV
35	Male	45	120.7	62	5.8	1.5	256	1042.8	540.9	1	0	1	1	1	IV
36	Male	24	151	55	5.2	1.17	410	2513	970.4	1	0	1	0	1	IV
37	Female	38	84	76	4.8	1.01	326	402.6	819.9	1	0	1	0	1	IV
38	Female	33	160	36	6.6	1.96	423	4613.8	2402	1	0	1	1	1	IV
39	Male	55	98	75	5.1	1.07	294	697.4	279.5	1	0	0	0	0	III
40	Female	29	75	93	5.5	0.98	270	556.8	569.2	1	0	1	1	0	IV
41	Female	26	76	94	5.1	0.87	293	1020.6	204.4	1	1	0	0	0	III-IV
42	Male	27	88	103	5.3	0.88	395	950.3	202.8	1	1	1	0	1	III-IV
43	Male	45	95	83	5.2	0.88	446	236.5	40.5	0	0	1	0	0	III-IV
44	Female	36	70	97	4.2	0.9	414	252	270.8	0	0	1	0	1	III
45	Female	49	99	58	5.8	1.19	274	716	1021.7	1	0	1	1	1	IV

46	Female	43	90	67	3.9	1.14	361	2406.6	963.4	0	0	1	0	0	III-IV
47	Female	31	69	102	2.9	0.75	303	500.4	1911.1	0	0	0	0	0	III
48	Male	28	108.4	80	4.5	1.25	435	3670.5	886.6	1	0	1	1	1	IV
49	Female	49	77.9	77	4.9	1.17	349	701	392	1	0	1	0	0	IV
50	Male	23	88	106	4.6	1.17	174	784.3	803.8	1	0	1	1	1	IV
51	Female	59	102	52	6.7	1.37	375	3909.4	2098.8	1	0	1	1	0	IV
52	Female	56	69.5	84	5.3	1.01	255	1350	501.4	0	0	1	0	0	III-IV
53	Male	40	122	64	6	1.3	454	1911.6	282.9	1	1	1	0	0	III-IV
54	Female	50	83	71	5.3	0.98	347	1453.5	578.7	1	0	1	1	1	IV
55	Male	50	104	72	3.7	1.08	144	137.6	8.5	0	0	0	0	1	III-IV
56	Male	45	172	40	5.8	1.74	360	1773.2	500.2	1	0	1	1	0	IV
57	Male	28	121	70	4.7	1.16	413	233.1	95.1	1	0	1	1	0	IV
58	Female	47	75	82	6.5	1.04	369	1520.2	529	0	1	1	1	1	IV
59	Male	28	90	100	4.5	0.94	417	1494	390.2	1	0	0	0	1	III
60	Female	48	137	39	9.6	1.65	445	1791.3	837	1	1	1	1	0	IV
61	Female	46	44	117	4.3	0.58	384	1166.4	1520.9	0	0	0	0	0	III
62	Male	27	127	66	5.5	1.16	489	3146.4	881.4	1	1	1	0	1	III-IV
63	Male	18	96	99	4.3	0.87	517	836	226.3	1	0	1	0	0	III

Table S5 General clinical information of 12 healthy control subjects included in this study

Healthy control sample	sex	Age,yr	Creatinine ,µmol/L	eGFR	BUN,mmol/L	Cysc,mg/L	Uric acid ,µmol/L	24U-pro ,mg/24h	Urine Albumin-to-Creatinine Ratio,mg/g
HC 1	Male	45	45	130	3.4	0.56	245	145	25.4
HC 2	Male	56	45	120	4.3	0.45	324	123	17.8
HC 3	Female	67	43	101	5.1	0.54	325	143	23.5
HC 4	Male	65	41	117	3.5	0.43	245	124	24.6
HC 5	Female	63	56	95	4.2	0.82	267	145	18.6
HC 6	Male	57	56	107	3.6	0.46	278	146	18.9
HC 7	Male	58	36	130	4.6	0.78	327	150	15.6
HC 8	Female	52	37	118	4.3	0.58	347	132	20.4
HC 9	Female	61	46	103	4.1	0.67	452	143	26.3
HC 10	Male	53	65	106	5.1	0.76	367	138	24.6
HC 11	Male	49	54	117	4.8	0.61	387	149	21.4
HC 12	Female	70	56	91	3.9	1.01	365	138	23.6

Table S6 All DEGs identified in the GSE93798 dataset

Gene symbol	logFC	AveExpr	t	P.Value	adj.P.Val	B
**FOSB**	-6.289019018	8.063847414	-23.38144236	4.28E-26	8.43E-22	48.30009791
**DUSP1**	-2.370905904	7.853314766	-20.31501751	1.11E-23	1.09E-19	43.18762435
**PCDH18**	1.610023084	6.315998763	17.4106943	4.20E-21	2.76E-17	37.60363865
**RNF186**	-2.018016661	6.604720111	-16.59059349	2.57E-20	1.27E-16	35.87589177
**FOS**	-4.621790837	8.325760192	-16.41786948	3.80E-20	1.50E-16	35.50280508
**ZFP36**	-2.327003482	9.473161679	-15.91527329	1.20E-19	3.96E-16	34.39840548
**EGR1**	-2.745621084	7.870757706	-15.81620778	1.52E-19	4.27E-16	34.17736886
**LYL1**	1.183061051	6.704706961	14.88599957	1.39E-18	3.42E-15	32.046372
**CEBPD**	-2.288957142	9.305391512	-14.61569777	2.69E-18	5.86E-15	31.4078549
**JUN**	-2.248258748	9.111591173	-14.57538739	2.97E-18	5.86E-15	31.31187061
**CYP27B1**	-2.27167444	5.643306493	-14.34206268	5.30E-18	9.50E-15	30.75237246
**CSRNP1**	-2.139593467	8.538650083	-13.82382527	1.96E-17	2.97E-14	29.48541832
**DEPDC7**	-2.505566689	4.84862484	-13.62952218	3.23E-17	4.54E-14	29.00164101
**ERRFI1**	-1.724939775	10.56367543	-13.48052912	4.75E-17	6.24E-14	28.62739751
**PPP1R10**	-1.036730772	7.448865362	-13.33470274	6.94E-17	8.55E-14	28.25833153
**ETNK2**	-1.001154203	5.794954643	-13.24593142	8.76E-17	1.01E-13	28.03231142
**SOX17**	1.739492592	6.648895465	13.15967335	1.10E-16	1.20E-13	27.81170552
**KLF4**	-2.219034936	6.824061089	-13.03571483	1.52E-16	1.58E-13	27.49297376
**ATF3**	-2.093246079	6.627960657	-12.86112716	2.43E-16	2.28E-13	27.04063008
**GATA3**	1.887230476	8.024489515	12.8359363	2.59E-16	2.28E-13	26.9750299
**COL1A2**	2.39493239	7.543250367	12.82584914	2.67E-16	2.28E-13	26.9487381
**APOLD1**	-3.768081152	8.967159782	-12.76420204	3.14E-16	2.48E-13	26.78776393
**C8orf4**	1.657909663	10.08171764	12.74043405	3.35E-16	2.54E-13	26.7255657

**SLC19A2**	-1.656797351	6.879320041	-12.70569974	3.68E-16	2.68E-13	26.63453473
**FPR3**	1.352092209	4.517118259	12.69195794	3.82E-16	2.69E-13	26.59847618
**EMP3**	1.068978312	7.950185853	12.66823975	4.07E-16	2.76E-13	26.53618038
**TIPARP**	-1.770088858	9.398886509	-12.39220547	8.60E-16	5.47E-13	25.80564942
**PER1**	-1.665091474	7.702722264	-12.26852299	1.21E-15	7.21E-13	25.47500485
**NR4A2**	-2.442532405	5.396133833	-12.06927761	2.09E-15	1.11E-12	24.93801701
**JUNB**	-1.878995417	7.839178416	-12.05010252	2.21E-15	1.14E-12	24.88605495
**GUCY1A3**	1.040248318	7.910769213	11.98550044	2.64E-15	1.33E-12	24.71062482
**GDF15**	-1.887634344	5.83268112	-11.70462481	5.79E-15	2.65E-12	23.94130628
**CD44**	1.050814499	6.522545633	11.55298224	8.90E-15	3.73E-12	23.5214945
**FXYD5**	1.111372573	6.701036845	11.50446321	1.02E-14	4.11E-12	23.38651104
**RASD1**	-2.630223752	8.375242167	-11.46279113	1.15E-14	4.47E-12	23.27032001
**NETO2**	1.023455156	6.480826797	11.39044556	1.41E-14	5.25E-12	23.06804181
**BHLHE40**	-1.737513108	6.987039078	-11.27253133	1.98E-14	7.23E-12	22.73682309
**PDE4B**	-1.215424132	7.173222976	-11.192373	2.50E-14	8.85E-12	22.5105768
**BRE-AS1**	-2.647193542	5.749064358	-11.18959062	2.52E-14	8.85E-12	22.50270781
**GSTA3**	-1.482543796	5.338985542	-11.17629826	2.61E-14	8.90E-12	22.46510047
**KCNK5**	-1.820754002	6.361329641	-11.17540176	2.62E-14	8.90E-12	22.46256316
**NR4A1**	-2.170498701	7.479667893	-11.12460024	3.04E-14	9.97E-12	22.3186049
**SYNPO2**	1.041420586	7.799855881	11.11713979	3.10E-14	1.00E-11	22.29743432
**KLF6**	-1.415310668	9.016389069	-11.03444367	3.94E-14	1.21E-11	22.06225844
**PHLDA1**	-1.610476003	7.692267323	-10.95261468	5.00E-14	1.43E-11	21.82863156
**PTGS1**	1.481740966	6.681142706	10.94921942	5.05E-14	1.43E-11	21.81891818
**SPRY2**	-1.513289974	8.004736918	-10.94680865	5.09E-14	1.43E-11	21.81202032
**RASL11B**	-1.226301496	10.47637132	-10.92551291	5.41E-14	1.46E-11	21.75105334

**CD69**	-2.840153867	6.936621465	-10.91815683	5.53E-14	1.46E-11	21.7299795
**NR1H4**	-1.12408522	5.315264351	-10.91680735	5.55E-14	1.46E-11	21.72611267
**BTG2**	-1.315704965	8.185199253	-10.88657199	6.07E-14	1.57E-11	21.63941095
**KLF9**	-1.150012653	7.49767663	-10.84801179	6.79E-14	1.74E-11	21.52865692
**SRPX2**	1.094323505	4.162152751	10.75933081	8.81E-14	2.20E-11	21.27317783
**RDH10**	-1.118861502	6.874104631	-10.75295354	8.98E-14	2.21E-11	21.25476458
**SNAI2**	1.279488106	9.197399691	10.74168006	9.28E-14	2.26E-11	21.22220087
**NFIL3**	-1.669225779	8.130204903	-10.68891717	1.08E-13	2.60E-11	21.06956505
**CTH**	-1.040060963	5.306394075	-10.61004824	1.37E-13	3.25E-11	20.84070547
**PDK4**	-1.918846043	10.32230478	-10.58259842	1.48E-13	3.48E-11	20.76085516
**MECOM**	1.221644043	8.849552247	10.54107042	1.68E-13	3.76E-11	20.63985874
**FN1**	1.459891585	7.352451777	10.45196624	2.19E-13	4.59E-11	20.37945978
**PCK1**	-1.884196328	9.828024846	-10.43586143	2.30E-13	4.65E-11	20.33228098
**CD34**	1.023894792	8.564410817	10.33809247	3.07E-13	5.97E-11	20.04512154
**ATOH8**	-1.53345873	8.195713463	-10.33011881	3.15E-13	6.02E-11	20.02164547
**PLD6**	-1.203702078	6.172391183	-10.26051222	3.88E-13	7.35E-11	19.81634886
**TGFBI**	1.234440069	7.584873149	10.20677738	4.56E-13	8.48E-11	19.6574224
**COL5A2**	1.101907726	6.029430857	10.18221126	4.91E-13	8.96E-11	19.58463762
**LINC00473**	-2.379659205	4.910491718	-10.17669784	4.99E-13	9.03E-11	19.5682914
**CYSLTR1**	1.104201544	5.921103149	10.13885058	5.60E-13	1.00E-10	19.45597269
**SYT11**	1.156226407	6.138479037	10.1244288	5.85E-13	1.04E-10	19.41312348
**IP6K3**	-2.308124021	6.586424158	-10.03440844	7.68E-13	1.29E-10	19.14503882
**RUNDC3B**	-1.211510593	4.50981674	-9.927843175	1.06E-12	1.71E-10	18.82630489
**CPNE4**	-1.536797726	6.725491489	-9.921626212	1.08E-12	1.73E-10	18.80766421
**ESM1**	-2.089218311	8.644590671	-9.911838163	1.11E-12	1.77E-10	18.7783059

**SOWAHC**	-1.360138838	8.031964461	-9.761743817	1.76E-12	2.63E-10	18.32655206
**NFKBIZ**	-1.685608267	8.183953761	-9.750596796	1.83E-12	2.70E-10	18.29288542
**HLX**	1.296792033	7.432455815	9.68839423	2.21E-12	3.23E-10	18.10472545
**CRYM**	-1.487373271	8.582356855	-9.609783396	2.82E-12	3.99E-10	17.86622231
**GSTA1**	-2.099860699	10.2395768	-9.539893266	3.49E-12	4.76E-10	17.65351729
**ECM1**	1.7339616	8.682260193	9.539119819	3.50E-12	4.76E-10	17.65115991
**MMP2**	1.112447836	6.168747468	9.530560303	3.60E-12	4.85E-10	17.62506637
**CMBL**	-1.289093049	6.205174437	-9.496996045	3.99E-12	5.24E-10	17.5226572
**SLC23A1**	-1.336430081	5.800488417	-9.48640119	4.12E-12	5.37E-10	17.4903014
**VNN1**	-1.415773321	5.554773341	-9.484707956	4.15E-12	5.37E-10	17.4851291
**ZNF331**	-1.518394433	7.173070406	-9.416437048	5.13E-12	6.47E-10	17.27628366
**WDR72**	-1.144178649	6.211806389	-9.290815799	7.58E-12	9.11E-10	16.89048573
**NUP58**	-1.027915114	4.574911725	-9.279234859	7.86E-12	9.33E-10	16.85482135
**ARG2**	-1.119054073	7.58000798	-9.263292285	8.26E-12	9.52E-10	16.80569811
**CRTAM**	1.407606905	3.707520866	9.2073562	9.85E-12	1.11E-09	16.63309918
**LOC727944**	-1.29119028	5.648465333	-9.205839176	9.89E-12	1.11E-09	16.62841288
**FOSL2**	-1.097363953	6.708410252	-9.173965893	1.09E-11	1.22E-09	16.52988747
**GADD45B**	-1.366893586	9.163535061	-9.138980788	1.22E-11	1.34E-09	16.42160178
**GPR65**	1.155557853	4.887829048	9.138254796	1.22E-11	1.34E-09	16.41935313
**MGST1**	-1.38675407	7.198178994	-9.10786955	1.35E-11	1.46E-09	16.32518298
**NR4A3**	-1.493203778	4.503890596	-9.101839502	1.37E-11	1.48E-09	16.30648147
**DDC**	-1.154160888	7.487089736	-9.097015495	1.39E-11	1.48E-09	16.29151724
**UGT2A3**	-1.435177284	6.178951175	-9.089361175	1.43E-11	1.51E-09	16.26776759
**SGK223**	1.005888062	5.888032015	9.084988759	1.45E-11	1.52E-09	16.25419782
**AK4**	-1.157071268	8.981002951	-9.081202339	1.46E-11	1.53E-09	16.24244484

**C3AR1**	1.281981295	7.066115702	9.068665909	1.52E-11	1.57E-09	16.20351984
**CDO1**	1.075867851	5.887901167	9.037167995	1.68E-11	1.71E-09	16.10563807
**ANKRD33B**	-1.338346316	9.501462404	-9.010207286	1.83E-11	1.83E-09	16.0217628
**ALDH6A1**	-1.058863769	8.995098871	-8.974450245	2.05E-11	2.02E-09	15.91039012
**HPD**	-2.32322933	7.90172066	-8.941184621	2.28E-11	2.22E-09	15.80664322
**RTN1**	1.139223122	5.216199194	8.90949914	2.52E-11	2.41E-09	15.70770474
**FNDC1**	1.027791972	3.38596896	8.904158623	2.56E-11	2.43E-09	15.69101744
**SHMT1**	-1.188970981	6.845941499	-8.876128138	2.80E-11	2.58E-09	15.60337774
**TOP2A**	1.218074162	3.539982973	8.868369804	2.87E-11	2.63E-09	15.57910468
**CXCL2**	-3.051234035	6.560892488	-8.853490685	3.01E-11	2.73E-09	15.53253392
**TSC22D3**	-1.017363113	10.62117946	-8.845730104	3.08E-11	2.79E-09	15.50823371
**NRARP**	1.427634252	7.468818013	8.803844475	3.52E-11	3.14E-09	15.37696143
**SH3GL2**	-1.301308955	5.099768856	-8.781145466	3.79E-11	3.33E-09	15.30573813
**CD14**	1.158529143	7.18587169	8.75922001	4.06E-11	3.48E-09	15.23688688
**RRM2**	1.628594383	4.561516323	8.757928352	4.08E-11	3.48E-09	15.23282907
**ID4**	-1.002785064	8.513542418	-8.754897852	4.12E-11	3.49E-09	15.22330788
**LPAR6**	1.242977474	9.173360371	8.706880048	4.80E-11	3.95E-09	15.07230903
**LRRC19**	-1.644605846	6.454156466	-8.701926007	4.87E-11	3.99E-09	15.05671573
**LRP2**	-1.286424537	8.661190544	-8.701273597	4.88E-11	3.99E-09	15.054662
**TWIST1**	1.27551725	5.612937223	8.699525752	4.91E-11	4.00E-09	15.04915973
**ACMSD**	-1.904893393	7.258665635	-8.667530079	5.44E-11	4.32E-09	14.94837653
**CEBPB**	-1.585145331	9.54705806	-8.663888879	5.50E-11	4.34E-09	14.93689995
**APOC1**	1.569600394	5.757480228	8.644646186	5.85E-11	4.58E-09	14.8762254
**TNFAIP3**	-1.777081396	7.856017294	-8.611993737	6.50E-11	5.00E-09	14.77317562
**GIPC2**	-1.245104917	5.966763785	-8.607419455	6.59E-11	5.05E-09	14.75873011

**DNAJB1**	-2.040880348	7.494325048	-8.586905566	7.04E-11	5.36E-09	14.69391968
**CSF1R**	1.375683298	6.957000639	8.568769196	7.46E-11	5.61E-09	14.6365828
**SLC7A9**	-1.936734039	7.920749543	-8.503732336	9.20E-11	6.76E-09	14.43068372
**COL3A1**	1.384614392	9.258772697	8.49273935	9.53E-11	6.93E-09	14.39583683
**FABP1**	-1.766891509	7.169673994	-8.481076137	9.89E-11	7.16E-09	14.35885145
**SULT1C2**	-1.203538289	6.541048295	-8.471975546	1.02E-10	7.35E-09	14.3299825
**SLC2A2**	-1.894747172	5.834970208	-8.424939179	1.19E-10	8.43E-09	14.18063554
**LINC01279**	1.362298843	5.471282845	8.417034512	1.22E-10	8.62E-09	14.15551458
**NAPSA**	-1.072578708	6.645339634	-8.413459773	1.23E-10	8.67E-09	14.14415197
**ESRRG**	-1.054269883	6.435782371	-8.40349796	1.27E-10	8.87E-09	14.11248056
**ANK2**	-1.181145928	7.211150934	-8.357455872	1.47E-10	1.00E-08	13.96596762
**CLEC5A**	1.26236854	3.205834527	8.354072225	1.49E-10	1.01E-08	13.95519182
**SLC17A1**	-1.39540118	5.863238267	-8.293469105	1.81E-10	1.19E-08	13.76199539
**ALDH8A1**	-1.593931907	8.616825324	-8.277757365	1.91E-10	1.25E-08	13.71184806
**MN1**	1.139641273	5.985639501	8.256591191	2.04E-10	1.33E-08	13.64425314
**TMEM252**	-1.248198628	6.033119878	-8.248872173	2.09E-10	1.36E-08	13.6195912
**ACADL**	-1.037473486	4.860475839	-8.204931723	2.41E-10	1.53E-08	13.47909244
**BHMT2**	-1.36334082	8.119010334	-8.185535267	2.57E-10	1.60E-08	13.4170131
**ALB**	-1.92731472	5.020482548	-8.18336685	2.59E-10	1.60E-08	13.41007072
**AEBP1**	1.155616754	9.017656675	8.130526308	3.07E-10	1.86E-08	13.24075848
**HGD**	-1.123961287	6.989100056	-8.06392548	3.82E-10	2.23E-08	13.02698164
**SLC17A3**	-2.096407038	7.290505157	-8.052404926	3.96E-10	2.28E-08	12.98996111
**ZNF189**	-1.032774773	8.583576895	-8.025034969	4.33E-10	2.47E-08	12.90196106
**AFM**	-1.782379939	5.931404897	-8.016441607	4.46E-10	2.52E-08	12.87431763
**KHK**	-1.059735753	7.409257738	-8.012172177	4.52E-10	2.54E-08	12.8605811

**MAFF**	-1.909067526	8.855816435	-8.005269726	4.62E-10	2.57E-08	12.83836959
**DUSP6**	-1.012761952	8.448696376	-7.986297423	4.92E-10	2.69E-08	12.77729634
**CYP8B1**	-1.268470573	5.68841341	-7.962776443	5.31E-10	2.83E-08	12.70153615
**BAG3**	-1.060022194	8.783841845	-7.959394453	5.37E-10	2.84E-08	12.69063887
**A1CF**	-1.073539697	5.500873928	-7.948727577	5.56E-10	2.91E-08	12.65626202
**ASS1**	-1.066376611	10.48221515	-7.927771492	5.95E-10	3.08E-08	12.5886965
**PBLD**	-1.103006874	7.35493137	-7.927252328	5.96E-10	3.08E-08	12.58702215
**IL33**	1.019320911	3.34177884	7.904083418	6.43E-10	3.27E-08	12.51227682
**SLC22A11**	-1.302223513	5.949719385	-7.899313342	6.54E-10	3.31E-08	12.49688233
**CRIP1**	1.060592173	7.063133863	7.890125939	6.74E-10	3.40E-08	12.46722629
**PHACTR3**	-2.102711565	8.286896368	-7.878449218	7.00E-10	3.50E-08	12.42952459
**COL1A1**	1.669758911	5.20719506	7.804135819	8.93E-10	4.27E-08	12.18931409
**USH1C**	-1.012134709	6.308045192	-7.794902714	9.20E-10	4.37E-08	12.15943716
**SDC1**	-1.121383207	7.246739343	-7.785420503	9.49E-10	4.50E-08	12.12874694
**CCL8**	1.199881475	4.289536961	7.778717906	9.70E-10	4.56E-08	12.10704886
**SLCO4C1**	-1.43542561	6.784793238	-7.77860226	9.71E-10	4.56E-08	12.10667445
**UCP2**	1.098740764	6.748928989	7.775895236	9.79E-10	4.58E-08	12.09791004
**MRO**	-1.345870553	5.377978489	-7.774525181	9.84E-10	4.59E-08	12.09347405
**APOM**	-1.19453206	7.659419035	-7.738891072	1.11E-09	5.08E-08	11.97804457
**PPP1R15A**	-1.088897645	7.485239592	-7.735207266	1.12E-09	5.11E-08	11.96610587
**G6PC**	-1.083494884	5.676507815	-7.733433963	1.13E-09	5.11E-08	11.96035847
**DIO1**	-1.8728561	7.409096409	-7.723845013	1.16E-09	5.26E-08	11.9292757
**SOX18**	1.040100763	4.604309438	7.7228738	1.17E-09	5.27E-08	11.92612709
**SLC27A2**	-2.005430825	8.628068376	-7.695415975	1.28E-09	5.70E-08	11.83708025
**GPAT3**	-1.452008761	9.185386459	-7.684999652	1.32E-09	5.86E-08	11.80328445

**TMEM200A**	1.424688911	8.391396321	7.677494029	1.35E-09	5.95E-08	11.77892727
**DPYS**	-1.982861937	8.006226232	-7.667139556	1.40E-09	6.09E-08	11.74531798
**COL15A1**	1.654340967	5.535389027	7.663580904	1.42E-09	6.15E-08	11.73376518
**MT1X**	-1.276634461	7.641284752	-7.648437381	1.49E-09	6.38E-08	11.68459258
**AZGP1**	-1.780249469	7.125734274	-7.630168289	1.58E-09	6.69E-08	11.62524807
**ALDOB**	-1.501549993	11.02704443	-7.582803776	1.85E-09	7.61E-08	11.47127682
**SLC4A4**	-1.108983678	6.873731992	-7.473306232	2.66E-09	1.03E-07	11.11471629
**DDIT4**	-1.490551439	9.358367642	-7.46478928	2.73E-09	1.06E-07	11.08694795
**RBP5**	-1.211455489	7.6926305	-7.451588407	2.85E-09	1.09E-07	11.04389887
**SLC13A3**	-1.084952817	7.304504783	-7.388488376	3.52E-09	1.30E-07	10.83796902
**FBP1**	-1.291080711	8.535864341	-7.384263299	3.57E-09	1.31E-07	10.82417128
**GADD45G**	-1.238640345	6.163200803	-7.349016285	4.01E-09	1.45E-07	10.70902314
**DMGDH**	-1.445030554	6.867087782	-7.346333744	4.04E-09	1.46E-07	10.70025648
**DUSP2**	-1.187615713	5.950987015	-7.340102831	4.13E-09	1.47E-07	10.67989192
**TMEM27**	-1.610545096	8.818426913	-7.328886419	4.28E-09	1.51E-07	10.64322737
**PPARGC1A**	-1.089989412	7.579419542	-7.293702226	4.81E-09	1.66E-07	10.52816827
**ARC**	-1.559490509	5.396501845	-7.2848937	4.96E-09	1.70E-07	10.49935151
**CTHRC1**	1.480077415	5.742540811	7.284469415	4.96E-09	1.70E-07	10.49796337
**ANKS1B**	-1.431641842	5.78071273	-7.281896739	5.01E-09	1.71E-07	10.48954606
**CLRN3**	-1.691869752	6.813761159	-7.271996937	5.17E-09	1.75E-07	10.45715231
**XPNPEP2**	-1.441221445	6.789357817	-7.252739357	5.52E-09	1.85E-07	10.39412271
**HCLS1**	1.029661555	7.222685733	7.22835979	5.98E-09	1.97E-07	10.31429979
**CYR61**	-1.191369362	8.98418478	-7.218926089	6.17E-09	2.02E-07	10.28340362
**OLFML3**	1.117410957	5.940623438	7.193149974	6.72E-09	2.14E-07	10.19896074
**ARRDC2**	-1.623556222	8.866255651	-7.186544717	6.87E-09	2.17E-07	10.17731628

**SFRP2**	1.275802185	4.092898151	7.17962519	7.03E-09	2.21E-07	10.15463961
**PLA2G4A**	1.110748177	5.517732217	7.140257638	8.02E-09	2.49E-07	10.02557855
**ARG1**	-1.21184609	3.500183778	-7.133278766	8.21E-09	2.54E-07	10.00269134
**FOXQ1**	-1.440179066	7.45855268	-7.113753436	8.76E-09	2.68E-07	9.938645643
**ENPP6**	-1.539213206	5.166447991	-7.109375569	8.88E-09	2.71E-07	9.924283181
**HRG**	-1.323790659	6.048355311	-7.087276933	9.56E-09	2.87E-07	9.851770673
**LOC284454**	-1.130181472	7.874252326	-7.079237788	9.82E-09	2.93E-07	9.825386207
**SLC22A6**	-1.300470613	6.705543672	-7.02202543	1.19E-08	3.45E-07	9.637533479
**CYP3A7**	-1.300131106	4.956439397	-7.020086734	1.20E-08	3.46E-07	9.631165472
**IFI30**	1.233127565	8.888232271	7.015361207	1.22E-08	3.51E-07	9.615642959
**LUM**	2.402815912	6.455873362	7.001840109	1.27E-08	3.64E-07	9.571223535
**SOCS3**	-1.152588542	5.996224948	-6.996919692	1.29E-08	3.68E-07	9.55505719
**TYROBP**	1.303691822	7.493023967	6.987369698	1.33E-08	3.78E-07	9.523677331
**SLC16A9**	-1.545964737	9.17378781	-6.975600337	1.39E-08	3.89E-07	9.485000062
**BBOX1**	-1.513974753	8.89353809	-6.953725269	1.49E-08	4.15E-07	9.413098694
**ITPRIP**	-1.033283194	6.874982062	-6.922164838	1.66E-08	4.55E-07	9.309331423
**MIOX**	-1.831860102	8.702546659	-6.904852789	1.76E-08	4.79E-07	9.252396385
**SLC5A12**	-1.468597346	7.316242955	-6.887273242	1.86E-08	5.06E-07	9.194571263
**SDPR**	-1.247914472	7.716605594	-6.844804787	2.15E-08	5.74E-07	9.054837195
**HHEX**	1.06549527	5.841369735	6.843054637	2.16E-08	5.77E-07	9.049077485
**SLC13A1**	-1.711056775	6.503843762	-6.832844999	2.24E-08	5.94E-07	9.015475973
**GJA4**	1.09227461	8.018889244	6.829031995	2.26E-08	6.00E-07	9.002926006
**GATM**	-1.402970123	10.38180115	-6.81356648	2.38E-08	6.27E-07	8.952019202
**DPEP1**	-1.162359206	7.727917944	-6.808160577	2.43E-08	6.36E-07	8.934223383
**NDC80**	1.152494544	5.740002532	6.794487672	2.54E-08	6.62E-07	8.889209702

**IL10RA**	1.192261538	6.313495037	6.789961225	2.58E-08	6.69E-07	8.874306727
**SLC3A1**	-1.084291605	10.2403658	-6.780672356	2.66E-08	6.87E-07	8.843722196
**GLYAT**	-1.497804477	7.364893583	-6.773889571	2.72E-08	7.00E-07	8.821387813
**PLG**	-1.541067027	7.908221867	-6.772671144	2.73E-08	7.01E-07	8.81737565
**PIPOX**	-1.701722282	8.750092013	-6.767751142	2.78E-08	7.11E-07	8.801174186
**ACE2**	-1.647323157	6.664097986	-6.745843095	2.99E-08	7.53E-07	8.729024394
**GBA3**	-1.51020569	7.23423336	-6.740111406	3.05E-08	7.66E-07	8.710146382
**FAM150B**	-1.326236507	4.957124949	-6.726988381	3.19E-08	7.97E-07	8.666921357
**MGAM**	-1.363456597	7.228803284	-6.711049878	3.36E-08	8.36E-07	8.614417669
**PMAIP1**	-1.171923069	4.466771891	-6.699716567	3.49E-08	8.63E-07	8.57708098
**IMPA2**	-1.030387011	8.383224621	-6.677305877	3.76E-08	9.19E-07	8.503243491
**FREM2**	-1.52640637	5.287664589	-6.649935898	4.12E-08	9.92E-07	8.413054352
**C10orf10**	-1.170104399	7.842159265	-6.633449471	4.36E-08	1.04E-06	8.358722717
**UCHL1**	1.285618289	8.145484583	6.614008875	4.65E-08	1.10E-06	8.29465045
**LOC100506498**	-1.766176375	4.226073028	-6.613217836	4.66E-08	1.10E-06	8.292043237
**MOXD1**	1.136982461	5.320943227	6.583372321	5.15E-08	1.20E-06	8.193668753
**TINAG**	-1.213135663	7.034005399	-6.582111263	5.17E-08	1.20E-06	8.189511927
**SLC47A2**	-1.300170529	6.745913669	-6.571781949	5.36E-08	1.24E-06	8.155462773
**MYC**	-1.549764202	7.037371765	-6.463132124	7.71E-08	1.69E-06	7.797266643
**SMIM3**	-1.012747196	7.183510054	-6.460799016	7.77E-08	1.70E-06	7.789574339
**MRLN**	-1.410993442	4.479426956	-6.451979449	8.00E-08	1.74E-06	7.760496011
**SMIM24**	-1.369242912	9.105685358	-6.451842482	8.01E-08	1.74E-06	7.760044425
**SLC22A8**	-1.363177592	7.799818382	-6.448875873	8.09E-08	1.75E-06	7.750263435
**SLC47A1**	-1.39192612	8.031048271	-6.424908771	8.76E-08	1.86E-06	7.671243416
**FAM151A**	-1.448639838	6.742167319	-6.415735681	9.04E-08	1.90E-06	7.640999786

**AGXT2**	-1.416894681	8.836575823	-6.366329973	1.07E-07	2.19E-06	7.478114934
**PSAT1**	-1.127931171	6.361742277	-6.361866653	1.08E-07	2.22E-06	7.463400558
**BNC1**	-1.340187825	3.677295998	-6.348856184	1.13E-07	2.31E-06	7.420509364
**GLYATL1**	-1.36036073	7.458746452	-6.334998434	1.18E-07	2.41E-06	7.374826526
**AGMAT**	-1.387904622	8.992768786	-6.320107266	1.25E-07	2.51E-06	7.325739011
**PLA2G7**	1.159915684	3.99250967	6.317317439	1.26E-07	2.53E-06	7.316542834
**CDKN1A**	-1.247999327	9.320896296	-6.314735543	1.27E-07	2.54E-06	7.308032141
**CYBB**	1.256469217	6.077320486	6.304020509	1.31E-07	2.61E-06	7.272713043
**AGT**	-1.220086168	6.349638743	-6.239614271	1.63E-07	3.15E-06	7.060449345
**DAO**	-1.151868088	6.459187734	-6.239115412	1.63E-07	3.16E-06	7.058805517
**C1QA**	1.131325283	5.188880683	6.220718194	1.74E-07	3.34E-06	6.998186577
**LOC284825**	-1.024303585	5.692881569	-6.219552389	1.74E-07	3.35E-06	6.994345461
**VSIG4**	1.303102805	5.086588774	6.20109164	1.86E-07	3.53E-06	6.93352431
**HTR2B**	1.736914948	6.316976227	6.179620221	1.99E-07	3.75E-06	6.862793371
**METTL7B**	-1.076562514	5.551929487	-6.149871389	2.20E-07	4.08E-06	6.764813081
**TAC1**	1.660781537	3.787374601	6.131461129	2.34E-07	4.33E-06	6.704188776
**CUBN**	-1.430301406	8.004837711	-6.0561206	3.02E-07	5.39E-06	6.456200069
**MPEG1**	1.250993725	6.993841556	6.045703431	3.12E-07	5.54E-06	6.421925963
**PAH**	-1.50632597	9.748157931	-6.024820697	3.35E-07	5.85E-06	6.353230438
**C15orf48**	1.63811012	4.187241255	5.974342985	3.97E-07	6.72E-06	6.187250228
**KCNJ15**	-1.074105528	7.60897141	-5.972770304	3.99E-07	6.75E-06	6.182080658
**RGS1**	-1.04163578	3.75066498	-5.938714377	4.47E-07	7.42E-06	6.070162173
**FMO1**	-1.506666367	8.616000167	-5.912448392	4.88E-07	7.96E-06	5.98388076
**MYRIP**	-1.028323863	6.344284514	-5.900301346	5.09E-07	8.26E-06	5.943990239
**POSTN**	1.182338645	10.62825308	5.861683558	5.79E-07	9.21E-06	5.817221289

**AKR1C1**	-1.161820697	6.760245511	-5.854761174	5.92E-07	9.32E-06	5.79450594
**MS4A7**	1.070955231	5.493960444	5.812475567	6.82E-07	1.05E-05	5.655807586
**NAT8**	-1.388005829	8.919251105	-5.787240276	7.42E-07	1.12E-05	5.573085542
**SUGCT**	-1.001687064	6.133769032	-5.785655384	7.46E-07	1.13E-05	5.567891533
**COL6A3**	1.476427191	6.074606577	5.768552585	7.90E-07	1.18E-05	5.511852395
**SOX9**	-1.065296122	5.842601393	-5.721099945	9.26E-07	1.35E-05	5.356469764
**CXCR2**	-1.400069143	6.590879963	-5.720431753	9.28E-07	1.35E-05	5.35428288
**HSPA1B**	-1.331954758	10.25424727	-5.704313248	9.79E-07	1.42E-05	5.301539256
**BHMT**	-1.249416198	9.898127794	-5.696166314	1.01E-06	1.45E-05	5.274887609
**IYD**	-1.03597658	4.576311457	-5.676424544	1.07E-06	1.53E-05	5.210325128
**EGR3**	-2.112774569	7.306039812	-5.638519639	1.22E-06	1.71E-05	5.086445655
**HBEGF**	-1.189117761	8.43081454	-5.636645558	1.23E-06	1.72E-05	5.080323756
**APOH**	-1.25007519	5.715959556	-5.606909496	1.36E-06	1.86E-05	4.983225506
**HAO2**	-1.339157789	6.784597869	-5.571832424	1.52E-06	2.06E-05	4.868781763
**APLNR**	1.175206228	9.461301458	5.569651291	1.53E-06	2.07E-05	4.861669012
**MT1M**	-1.550108784	7.521496596	-5.526935852	1.77E-06	2.35E-05	4.722457852
**SLC25A18**	-1.350176933	5.689294492	-5.479998518	2.07E-06	2.67E-05	4.569682548
**CX3CR1**	1.36012187	7.37998102	5.476902576	2.09E-06	2.69E-05	4.559613108
**CD36**	1.053081585	4.659179208	5.458913974	2.22E-06	2.81E-05	4.501124653
**TOX3**	-1.061174298	4.840912259	-5.449423907	2.29E-06	2.89E-05	4.470281588
**C1QB**	1.342155839	5.153302103	5.411673697	2.60E-06	3.21E-05	4.34768407
**SERPINA1**	-1.222663852	7.717605383	-5.404849599	2.66E-06	3.26E-05	4.325538179
**AREG**	-1.864234092	5.904274577	-5.397032893	2.72E-06	3.33E-05	4.300177194
**C2CD4A**	-1.115751047	3.655600409	-5.377327376	2.91E-06	3.52E-05	4.236273027
**FGB**	-1.352671819	4.515614904	-5.364195553	3.04E-06	3.64E-05	4.193710973

**SERPINE1**	-1.374268857	7.490851687	-5.351382178	3.17E-06	3.77E-05	4.152199793
**CLDN2**	-1.126919149	7.17423296	-5.347425244	3.21E-06	3.81E-05	4.139384398
**THBS2**	1.078850251	5.971469145	5.304621455	3.70E-06	4.29E-05	4.000871642
**OMD**	1.088813148	4.143289776	5.264429929	4.23E-06	4.81E-05	3.871012938
**CTXN3**	-1.55874656	6.389430354	-5.258980698	4.30E-06	4.89E-05	3.853421938
**LYZ**	1.256096188	8.738803673	5.246261279	4.49E-06	5.08E-05	3.812376254
**HSPA6**	-1.177455116	5.957398228	-5.155085045	6.07E-06	6.50E-05	3.518773601
**SLC23A3**	-1.311203732	7.185616129	-5.119824457	6.81E-06	7.17E-05	3.405536339
**AOC1**	-1.264245054	8.202132516	-5.103385676	7.19E-06	7.51E-05	3.352805628
**CALB1**	-1.934861355	7.667483188	-5.081540273	7.73E-06	7.98E-05	3.28279399
**MT1G**	-1.302137213	10.55713844	-5.060423012	8.28E-06	8.44E-05	3.21518437
**RNF212B**	-1.243982199	6.589784577	-5.016138646	9.58E-06	9.56E-05	3.073626705
**SLC10A2**	-1.498081268	5.729867958	-4.992375037	1.04E-05	0.00010184	2.997793636
**CRISPLD2**	-1.211871675	7.642179345	-4.978901414	1.08E-05	0.000105483	2.954838317
**ERAP2**	-1.397203265	6.578253216	-4.966721565	1.13E-05	0.000108925	2.916033551
**IL1B**	1.13139983	6.457648052	4.941720474	1.22E-05	0.000116365	2.83645867
**SUCNR1**	1.112614863	6.809913096	4.93345656	1.26E-05	0.000119147	2.810179228
**TMED6**	-1.604936893	5.697356	-4.898565205	1.41E-05	0.000130933	2.699354652
**UBD**	1.228426707	7.255398608	4.890542441	1.44E-05	0.000133779	2.673902476
**CYP4F2**	-1.459484122	6.633060117	-4.82957623	1.76E-05	0.00015782	2.480869122
**COL21A1**	1.108103335	5.505118273	4.695599159	2.72E-05	0.000227798	2.059172004
**S100A12**	-1.495177088	5.456803587	-4.657523527	3.08E-05	0.000252335	1.939996179
**LOC100505985**	-1.32719119	6.582055467	-4.613434153	3.54E-05	0.000284313	1.802388048
**FOSL1**	-1.360205458	3.748695138	-4.603240108	3.66E-05	0.00029212	1.770632285
**TMEM213**	-1.255555623	5.984419556	-4.584213934	3.89E-05	0.000307173	1.711425752

**IGSF6**	1.046346569	5.78955266	4.51430616	4.87E-05	0.000366523	1.494598987
**FCAMR**	-1.014512796	5.723241289	-4.481199777	5.41E-05	0.000399017	1.392319979
**ERP27**	-1.100703966	8.042162563	-4.406311334	6.87E-05	0.000486219	1.161958497
**LOC101927451**	1.012903236	5.018310999	4.390231616	7.23E-05	0.000506756	1.112682237
**CHODL**	1.413059342	6.392561808	4.34010508	8.47E-05	0.000576763	0.959505092
**FCGR3B**	-1.058496531	7.722305316	-4.276335373	0.000103607	0.000680194	0.765618057
**CXCL11**	1.164081441	4.478914312	4.219357639	0.000123897	0.000788695	0.593345964
**S100A8**	-1.288344245	8.495074548	-4.144931222	0.000156314	0.000957914	0.369746828
**RBP4**	-1.04592867	6.207877112	-4.053444257	0.0002076	0.001217304	0.097215373
**S100A2**	-1.21738441	7.563901155	-4.012481897	0.000235553	0.001356183	-0.023942315
**IDO1**	1.143679623	6.562489087	4.01067743	0.000236865	0.001361103	-0.029266893
**IGHM**	1.193706521	4.876696664	3.981433054	0.000259141	0.001465937	-0.115409643
**HBB**	1.072981734	10.82211473	3.955382067	0.000280687	0.001567044	-0.191903936
**COLEC12**	1.218362878	5.041878046	3.941545762	0.000292827	0.001622861	-0.232437953
**CCL4**	1.508216088	7.59749237	3.87753265	0.000355932	0.001907663	-0.419101795
**AKR1B10**	-1.057594341	4.569231211	-3.782617773	0.000474297	0.00241525	-0.693170952
**APOD**	-1.245667911	10.06749415	-3.732241229	0.000551732	0.002736714	-0.837270768

**CXCL10**	1.149027664	7.159456875	3.639428552	0.000727423	0.00343768	-1.100173581
**IGFBP1**	-1.260902258	6.280725548	-3.347018234	0.001703721	0.006968385	-1.904854721
**CLDN8**	-1.093035536	5.916999417	-3.344478602	0.001716119	0.007006004	-1.911676248
**HLA-DQA1**	2.166606518	8.142000617	3.148605707	0.002978418	0.01101366	-2.428428857
**IGKC**	1.355694662	7.601493376	2.876372991	0.006233546	0.020226173	-3.113699871
**JCHAIN**	1.112509598	3.766868739	2.797473196	0.007671488	0.023937861	-3.304581838
**SELE**	1.107713848	5.533038687	2.6313572	0.011755876	0.033787638	-3.69431968

Table S7 All DEGs identified in the GSE37460 dataset

Gene symbol	logFC	AveExpr	t	P.Value	adj.P.Val	B
**CDH5**	1.552352723	9.059642029	8.810023997	1.28E-10	2.56E-07	14.08536641
**COL4A3BP**	1.020623238	6.733354508	4.415497914	8.43E-05	0.000905193	1.348334484
**TSPAN2**	1.062650568	7.912157564	3.53015035	0.001130546	0.005674113	-1.09118365
**CDH13**	1.061065704	8.888634233	3.555389011	0.001052768	0.005399725	-1.024831614
**LPAR6**	1.08200333	10.32449637	7.456817564	7.02E-09	4.87E-06	10.29696148
**RCAN2**	1.17225988	10.34341681	6.225380591	3.11E-07	4.12E-05	6.685454266
**KLF2**	1.121279954	10.42502054	4.294100407	0.000121529	0.001157328	1.002437591
**CD52**	1.071371199	7.403202471	2.836466299	0.00735492	0.022814367	-2.813361904
**POSTN**	1.432416223	10.93084866	4.589268447	4.98E-05	0.000658987	1.848113092
**PLK2**	1.023211197	8.194383171	4.25826379	0.000135317	0.001240184	0.90089336
**FGL2**	1.49291306	10.10141766	6.525146346	1.22E-07	2.66E-05	7.574177557
**IFI44L**	1.185281233	8.200998172	2.478913071	0.017859468	0.045498183	-3.608715486
**TMSB15A**	1.037597981	9.553104865	2.969439913	0.00521063	0.017645625	-2.50009108
**RAPGEF4**	1.544759441	7.34864945	7.844863322	2.18E-09	1.74E-06	11.40633857
**VSIG4**	1.034838309	7.89182041	2.301011312	0.02712305	0.06364897	-3.976321441
**COL1A2**	1.199459246	8.746935938	2.64393376	0.01194836	0.033259097	-3.250572794
**COL4A1**	1.289359053	10.92710325	7.376882224	8.95E-09	5.10E-06	10.06640428
**COL6A3**	1.363632558	7.669465257	2.656095181	0.01159357	0.03249079	-3.223557143
**COL15A1**	1.037987751	7.07553577	2.710580614	0.010120368	0.029239063	-3.101508441
**CFD**	1.007326037	7.353987358	3.312214849	0.002074655	0.008787165	-1.654302241
**AGTR1**	1.215635085	9.222869303	4.969823501	1.55E-05	0.000321255	2.957940947
**ECM1**	1.336145285	10.16254169	3.228222656	0.002610386	0.01044591	-1.866314435
**S1PR1**	1.104918864	9.15197811	5.090173505	1.07E-05	0.000263868	3.31228503

**EDNRB**	1.376504005	7.274342848	3.652980142	0.000797751	0.004444294	-0.766191609
**MECOM**	1.759858032	7.114919723	5.429020419	3.72E-06	0.000153085	4.315669392
**F8**	1.144741315	10.05976967	6.089736842	4.74E-07	5.25E-05	6.282161391
**FCN1**	1.104586425	8.453004814	2.460404437	0.01866726	0.047035831	-3.647877072
**CD93**	1.122248645	9.093177333	6.268497288	2.72E-07	3.90E-05	6.813523319
**FOXO3**	1.326214884	8.409683091	4.158381804	0.000182347	0.001524147	0.619336265
**FN1**	1.033271616	8.3177039	3.03002465	0.004442537	0.015596473	-2.35447881
**HMHA1**	1.066278985	8.666052782	6.205326219	3.31E-07	4.21E-05	6.625864584
**LY96**	1.410177724	7.30839176	5.354959415	4.69E-06	0.000164776	4.095798269
**SOSTDC1**	1.24285443	8.106526654	3.885228906	0.000407962	0.00271883	-0.138380005
**RWDD3**	1.073472291	6.537609738	5.855180533	9.85E-07	8.09E-05	5.583876506
**GATA3**	1.183854271	7.985318754	6.339586904	2.18E-07	3.43E-05	7.024522533
**GBP2**	1.076319588	8.946956518	6.0285292	5.74E-07	5.92E-05	6.100024625
**TSPAN13**	1.020814865	9.578781184	4.776042272	2.81E-05	0.000462273	2.390516051
**SLCO3A1**	1.21246117	7.662693203	6.352062536	2.09E-07	3.43E-05	7.061529247
**CNIH4**	1.209720009	8.572925424	6.259757829	2.79E-07	3.90E-05	6.787570123
**PYCARD**	1.131257831	7.742670582	4.089804355	0.00022354	0.001766039	0.427340924
**HBA2**	3.553117694	12.06980329	7.26119091	1.27E-08	6.86E-06	9.731571437
**HBB**	3.17214727	11.19713547	6.302372027	2.44E-07	3.66E-05	6.91409172
**HCLS1**	1.10492492	9.593180539	5.01558973	1.34E-05	0.000295838	3.092533412
**HLA-DQB1**	1.044983721	9.841434014	3.697697434	0.000701899	0.004059676	-0.646616806
**HLX**	1.216152414	8.487456138	6.644162486	8.47E-08	2.26E-05	7.925726458
**HOXA7**	1.011337841	6.793314745	4.15539635	0.000183975	0.001532398	0.610954982
**HTR2B**	1.413193686	6.786578583	3.514053201	0.001183007	0.005864216	-1.133384312
**ID1**	1.178851585	10.65116876	5.773819396	1.27E-06	9.16E-05	5.341562359

**IL10RA**	1.029289426	7.756381653	3.117645803	0.00351838	0.013080176	-2.140840391
**IL13RA2**	1.022863349	11.34125394	3.009513387	0.004689768	0.016292277	-2.403972856
**ITGB2**	1.156373782	7.870179471	3.369502851	0.00177131	0.007864378	-1.508047531
**KDR**	1.208370562	10.53174522	4.746482164	3.08E-05	0.000489332	2.304351349
**TNPO1**	1.147787312	9.428765149	4.2679133	0.00013146	0.00121797	0.928209142
**LTF**	1.217728327	8.199522725	2.717111202	0.009955931	0.02887558	-3.086769736
**MATN2**	1.14133209	9.449793107	6.536962036	1.18E-07	2.66E-05	7.609116209
**NCF2**	1.012119366	8.040017315	2.82995361	0.007478675	0.023101986	-2.828474553
**GIMAP6**	1.103406278	10.25465408	4.863461389	2.15E-05	0.000398247	2.645968076
**NNMT**	1.185642328	7.51666121	2.365264397	0.023368363	0.056216611	-3.84584928
**NPY1R**	1.062283698	9.917020684	5.325609771	5.13E-06	0.000173942	4.008738385
**GOLT1B**	1.026835172	7.358042296	5.747112143	1.38E-06	9.67E-05	5.262025704
**GLTP**	1.193240016	6.672622226	5.82730132	1.07E-06	8.63E-05	5.500844803
**PCDH12**	1.10757492	9.061615107	5.899383597	8.58E-07	7.57E-05	5.715519361
**MS4A4A**	1.010793419	5.924519999	2.048418337	0.047660624	0.098287711	-4.462664045
**PCYOX1**	1.007966289	7.435814943	3.188404015	0.002908132	0.01134513	-1.965785704
**PDGFRA**	1.336710925	9.008859602	5.577109591	2.35E-06	0.000124753	4.755946219
**KLF13**	1.082898283	6.554022677	5.244922536	6.60E-06	0.00020169	3.769641232
**PECAM1**	1.305287209	9.909293336	6.386124359	1.88E-07	3.27E-05	7.16253194
**FXYD6**	1.102153139	10.3111038	5.988024304	6.51E-07	6.54E-05	5.979453884
**EXOSC10**	1.129299347	7.652999597	4.97752067	1.51E-05	0.000316455	2.980563158
**SOX18**	1.161483643	6.138122087	5.603361664	2.16E-06	0.000120775	4.834061912
**MPHOSPH8**	1.067566128	8.165042264	4.968947125	1.55E-05	0.000321497	2.955365622
**XAF1**	1.071221999	7.523052639	3.281204422	0.002258933	0.009362125	-1.73291868
**DPP8**	1.02366165	10.10240276	4.219461066	0.000151977	0.001346513	0.79125156

**RCBTB1**	1.042635781	8.41338151	6.4271105	1.66E-07	3.10E-05	7.283994902
**GIMAP4**	1.177784109	10.55796008	5.859240054	9.73E-07	8.09E-05	5.59596669
**C8orf4**	1.834724813	9.574594235	11.4143524	1.10E-13	1.32E-09	20.65337353
**KIAA1462**	1.178024458	7.73631875	4.826093168	2.41E-05	0.000421629	2.536660093
**PTPRB**	1.046827211	8.805374698	3.739549845	0.000622339	0.00371806	-0.534120946
**RALA**	1.389771048	6.887841259	5.416756272	3.87E-06	0.000153085	4.279242934
**ACTA2**	1.355590822	10.88535578	5.575530423	2.36E-06	0.000124753	4.751247774
**SOX17**	1.398297591	6.712229846	7.914777671	1.77E-09	1.60E-06	11.60437779
**ST3GAL1**	1.091891502	7.757937959	3.547841563	0.00107547	0.005493429	-1.044697348
**SLC14A1**	1.359511839	8.444283768	4.51287923	6.28E-05	0.0007568	1.627781573
**SLCO2A1**	1.159555924	10.78506775	4.356017452	0.000100881	0.001025177	1.178498679
**SNAI2**	1.274023789	9.589178625	6.341562816	2.16E-07	3.43E-05	7.030384173
**TCF4**	1.188092524	6.814730015	3.983309117	0.000306105	0.002221881	0.131473426
**TGFBI**	1.033497645	9.09778333	2.737175664	0.009466085	0.027839646	-3.041341213
**C1QA**	1.647305039	7.662639505	3.539302117	0.001101725	0.005581418	-1.067149883
**C1QB**	1.596988869	7.381863251	3.038352017	0.004345714	0.015324023	-2.334328033
**PHLDA2**	1.228365682	6.270189758	3.574609348	0.000997018	0.005178336	-0.974151363
**TYROBP**	1.433864429	8.618173788	3.685843875	0.000726163	0.004151836	-0.678376712
**ZNF148**	1.070636681	7.108647884	4.424849358	8.20E-05	0.000893598	1.375096117
**NETO2**	1.498491956	7.860988326	5.710944636	1.54E-06	0.000100784	5.154324284
**DYSF**	1.113336759	10.56016054	5.64792564	1.88E-06	0.000110219	4.966700294
**SRPX**	1.042160747	7.127189499	4.254518478	0.000136844	0.00124575	0.890296453
**MAGT1**	1.496877812	8.069826051	5.174608604	8.21E-06	0.000230152	3.561615344
**FCN3**	1.074019698	11.58531639	3.413354274	0.001568274	0.007172647	-1.395223522
**PPAP2B**	1.214924222	11.12246007	4.384658438	9.25E-05	0.000959938	1.260194177

**HYAL2**	1.056538791	9.910179762	6.769473645	5.75E-08	1.86E-05	8.294886184
**ARHGEF6**	1.035591141	9.036024604	7.249321426	1.32E-08	6.86E-06	9.697144485
**GMFG**	1.331397679	8.959294476	6.747181547	6.16E-08	1.92E-05	8.229293901
**CD53**	1.04995358	9.722248685	3.110757108	0.003583875	0.013242734	-2.157763775
**ISG15**	1.049645795	9.546434613	2.845554053	0.007185426	0.022387311	-2.792237151
**CD97**	1.096187154	8.315328995	6.030260957	5.71E-07	5.92E-05	6.105178898
**TRIB1**	-1.491711965	8.255223581	-5.384652304	4.27E-06	0.000158683	4.183919928
**HRSP12**	-1.104862449	9.215028863	-3.388911412	0.001678523	0.007556195	-1.458203789
**GLYAT**	-1.372095951	9.168020276	-3.422277717	0.001529781	0.007059301	-1.37217396
**SLC17A3**	-1.064539879	9.536682249	-2.328758855	0.02543964	0.060400027	-3.920302144
**ALDH1L1**	-1.031375018	8.717257269	-2.608513058	0.013039548	0.035584311	-3.328779683
**FTCD**	-1.351917778	8.288068539	-3.969987465	0.000318325	0.002287015	0.094668156
**SLC27A2**	-1.355433435	9.30019277	-2.975554575	0.005127782	0.017454012	-2.485474796
**SLC7A9**	-1.37404893	9.919865547	-2.860114523	0.006921449	0.021797643	-2.758302238
**SUPT16H**	-1.12233501	9.999968259	-7.778170966	2.67E-09	1.99E-06	11.21688733
**CISH**	-1.016934871	8.5939087	-5.513064172	2.86E-06	0.000139459	4.565448333
**SIK1**	-2.346134951	7.561520538	-8.943928498	8.74E-11	2.48E-07	14.44718161
**CYP27B1**	-2.309864818	8.056781774	-8.032017259	1.25E-09	1.25E-06	11.93514606
**AFM**	-1.105737784	6.898236458	-3.270612309	0.002325376	0.009561299	-1.759680854
**DPYS**	-1.192578066	8.82375325	-2.554046543	0.014897443	0.039465851	-3.447631286
**AGT**	-1.097415972	7.843369716	-2.8979699	0.006276954	0.020258741	-2.669569711
**EGR1**	-1.465114905	8.705837432	-5.150753197	8.84E-06	0.000239678	3.491116908
**EGR2**	-1.245315226	6.700681561	-2.589464361	0.013663611	0.036916751	-3.370541364
**EGR3**	-1.402932959	6.53626771	-3.354874043	0.001844467	0.008090089	-1.545518622
**ALB**	-2.551299538	7.276346735	-4.645893499	4.19E-05	0.0005953	2.012028858

**FABP1**	-1.0984944	7.324101442	-2.513326168	0.01644174	0.042557813	-3.535352454
**FBP1**	-1.211960032	10.24513365	-3.339755759	0.001923103	0.00834624	-1.584154489
**SYNE2**	-1.081983129	8.833932077	-5.488278323	3.09E-06	0.000142481	4.491758238
**FOS**	-1.188835732	8.688825221	-2.37558681	0.022810967	0.05505286	-3.824643246
**FOSB**	-3.341766962	7.494501371	-9.816772788	7.55E-12	4.51E-08	16.74271588
**G6PC**	-1.114683087	5.658903924	-3.14175755	0.003297948	0.012475257	-2.081437472
**GHR**	-1.007894323	10.63338424	-5.447315976	3.51E-06	0.000150626	4.37002154
**GK**	-1.079402694	7.966286375	-4.044691517	0.000255455	0.001954891	0.301657966
**GSTA1**	-1.635867298	10.26326078	-3.038754085	0.00434109	0.015312232	-2.333354275
**NR4A1**	-1.325952802	8.385455378	-8.528508345	2.90E-10	4.16E-07	13.31661685
**HPD**	-1.490040135	9.03146323	-2.621076732	0.012642416	0.03476624	-3.30112157
**APOD**	-1.269297949	10.66012299	-2.484919789	0.017604241	0.044963555	-3.595961505
**IGF1**	-1.089669577	8.160322507	-3.281234488	0.002258747	0.009362125	-1.732842649
**IGFBP1**	-1.161332861	6.348828466	-2.241788239	0.031054677	0.070657217	-4.094209001
**APOH**	-1.599830799	6.798838274	-5.725415397	1.48E-06	0.000100533	5.197414583
**KNG1**	-1.183726365	8.836744633	-2.241938605	0.031044081	0.070653357	-4.09391261
**ALDH6A1**	-1.173824973	9.341034872	-3.166797014	0.003082925	0.011879788	-2.019475307
**MT1G**	-1.186524426	12.08204496	-3.756090344	0.000593366	0.003584469	-0.489509814
**ATF3**	-2.260742787	8.786199622	-7.431210997	7.59E-09	4.87E-06	10.22317653
**NFIL3**	-1.161818473	8.549887512	-6.157176689	3.84E-07	4.64E-05	6.4827395
**NR4A2**	-1.56229012	5.571102474	-8.065203067	1.13E-09	1.23E-06	12.02846686
**PAH**	-1.002858968	10.80372515	-2.238721536	0.031271487	0.071082908	-4.100250572
**SERPINA5**	-1.071301782	8.944461082	-2.140890698	0.038935496	0.083941506	-4.289667908
**PCK1**	-1.383859169	10.6604668	-2.915822855	0.005992912	0.019579683	-2.62747177
**HAO2**	-1.443899382	9.109771323	-2.950445715	0.005476082	0.01832664	-2.545378125

**UPB1**	-1.264628559	7.485189413	-3.23385978	0.002570653	0.010322489	-1.852177539
**SERPINA1**	-1.291844107	9.832523765	-3.846577693	0.00045657	0.002961515	-0.243987047
**PLG**	-1.168485396	9.02760318	-2.064928683	0.045987352	0.095628428	-4.432216319
**RIPK4**	-1.155817957	7.921586565	-5.04338837	1.23E-05	0.000282553	3.174381242
**CYCS**	-1.036362474	10.1510195	-4.740307728	3.14E-05	0.000495138	2.286367854
**SLC22A11**	-1.034313121	7.958077693	-3.05829424	0.004121875	0.014677506	-2.285939327
**APOM**	-1.250406681	9.114313449	-2.971080856	0.005188274	0.017594825	-2.496170404
**AZGP1**	-1.169649123	7.373095082	-2.243925824	0.030904349	0.070390013	-4.089994152
**DNAJC12**	-1.099802587	7.980267414	-3.673739806	0.000751777	0.004264847	-0.710760923
**PSG5**	-1.191161147	7.066126584	-6.052130577	5.33E-07	5.69E-05	6.17026503
**GBA3**	-1.135654087	8.025412761	-3.186751565	0.00292116	0.011377401	-1.969898936
**PRODH2**	-1.224670465	10.29153703	-3.486902274	0.001276837	0.006215812	-1.204351787
**ACE2**	-1.073387102	7.413174936	-2.54433142	0.015253307	0.04017723	-3.468648913
**RBP4**	-1.503149019	7.671206292	-2.833610568	0.007408948	0.022934374	-2.819991315
**PBLD**	-1.178126374	9.421179714	-3.625864702	0.000861903	0.004671092	-0.83837821
**SLC13A3**	-1.065365184	9.150678132	-2.719560931	0.009894889	0.028754318	-3.081234991
**SLC2A2**	-1.449625298	6.560089784	-4.299643963	0.000119523	0.001141861	1.018169209
**SLC10A2**	-1.02141606	7.201454832	-3.227197217	0.002617676	0.010470535	-1.868884601
**SLC17A1**	-1.057718477	7.597580598	-2.686835298	0.010739701	0.030628891	-3.154900987
**TFRC**	-1.071044067	9.060853464	-5.378738469	4.35E-06	0.000160139	4.166365676
**KLF10**	-1.007394187	10.47383143	-6.017555621	5.94E-07	6.02E-05	6.067362396
**UMOD**	-1.932676382	10.79751351	-2.54548426	0.015210675	0.04009932	-3.466157769
**XPNPEP2**	-1.264430507	7.821286788	-2.81719434	0.007726778	0.023720616	-2.858018445
**CRISPLD2**	-1.203473314	8.255596506	-3.686392519	0.000725023	0.004147296	-0.676907701
**ARID5B**	-1.728444452	9.754701516	-6.948540478	3.31E-08	1.28E-05	8.820425695

**CMAH**	-1.03573809	8.377270186	-3.947854094	0.000339686	0.002388587	0.033622234
**SERPINA6**	-1.228765466	7.312265038	-3.454809072	0.001396974	0.006638543	-1.287888885
**ZNF160**	-1.02924176	5.915684518	-4.245382896	0.000140639	0.001269563	0.864460954
**SLC22A6**	-1.104164009	7.950999497	-2.518905567	0.016221823	0.042189437	-3.523391259
**SLC22A8**	-1.770381656	8.008869277	-4.319463222	0.000112612	0.00109547	1.074463881
**ACY1**	-1.091683834	10.50124246	-2.815506009	0.007760177	0.023793765	-2.861921389
**GDF15**	-1.406852915	7.053575515	-3.987570505	0.000302293	0.002208751	0.143256738
**CXCL14**	-1.134964481	10.5747387	-2.439885267	0.019601572	0.048834493	-3.691048451

Table S8 All DEGs identified in the GSE104948 dataset

Gene symbol	logFC	AveExpr	t	P.Value	adj.P.Val	B
**ARPC1B**	1.360885162	10.36448289	4.885782955	3.04E-05	1.18E-02	2.421870674
**LPAR6**	1.450233058	10.442624	6.371822266	4.46E-07	0.000672562	6.253895084
**CD52**	1.887398653	7.439724943	3.22686704	0.002970322	0.102761217	-1.731900062
**IFI30**	2.149995268	11.2166439	4.82860117	3.58E-05	0.012010107	2.273374987
**IFI44**	1.472088714	9.380521976	3.372721136	2.03E-03	8.38E-02	-1.389266322
**POSTN**	1.421473838	11.14037014	2.661508353	1.23E-02	2.04E-01	-2.98632081
**HCP5**	1.131826817	9.19820908	3.666903433	0.000921908	0.058896643	-0.679628048
**FGL2**	1.320446064	10.29808521	5.138447993	1.48E-05	0.006615243	3.078767183
**IFI44L**	1.540730494	8.328950322	2.077402699	4.62E-02	0.352280749	-4.122030393
**GLIPR1**	1.176642928	7.234930791	2.851764664	0.007705281	0.165855275	-2.578804045
**TMSB15A**	1.838050816	9.547429812	3.726683169	7.84E-04	5.38E-02	-0.532782777
**ADAMTS5**	1.300642256	7.088110997	2.975984254	0.005648774	0.144775357	-2.304312962
**PRSS23**	1.356152562	10.18305852	2.629818072	0.013226327	0.208820308	-3.052577457
**CORO1A**	1.226272969	7.320316783	3.304563472	2.42E-03	9.15E-02	-1.550211558
**CD300A**	1.184994049	7.0133043	2.950012942	0.006030321	0.148895893	-2.362220859
**COL1A2**	2.256204517	8.841113599	2.929958974	0.006341462	0.153440504	-2.406750458
**COL3A1**	1.834150781	10.32634563	4.334073686	1.45E-04	2.29E-02	0.996635101
**COL4A2**	1.002280572	9.97635417	3.227181588	0.002967893	0.102761217	-1.731168473
**COL6A3**	2.050548378	7.810978951	2.304269178	0.028137242	0.288613415	-3.703583093
**CSF1R**	1.396328336	8.417158236	3.10678447	0.004050392	0.121225366	-2.008723046
**CSF2RB**	1.261373678	8.584319632	2.40914132	2.22E-02	0.261881445	-3.500027357
**CSTA**	2.084641381	6.767904999	4.167987355	0.000231687	0.030406402	0.572822805

**CTSS**	1.707389595	7.655448702	3.598433664	1.11E-03	0.063586715	-0.846794681
**CX3CR1**	3.132192485	8.709849726	6.596553462	2.38E-07	0.000603163	6.819161798
**CYBB**	1.532654741	6.933826678	2.868036802	7.40E-03	0.163651966	-2.543210128
**CFD**	1.033154007	7.415769529	2.078486298	4.61E-02	3.52E-01	-4.120106558
**DOCK2**	1.541909379	6.742175219	3.759388986	0.000716847	0.051807147	-0.452106793
**APLNR**	1.595903984	10.66681863	4.536156125	8.22E-05	1.74E-02	1.516240308
**APOBEC3A**	1.399958156	6.904207151	2.3333476	0.026354596	0.280117022	-3.647758053
**EMP1**	1.106515789	8.332418621	2.663674801	0.012195332	0.203903015	-2.981773776
**MECOM**	1.201573004	7.769111426	3.091560116	4.21E-03	1.24E-01	-2.043456197
**EVI2A**	1.441098425	7.156738716	2.332758247	2.64E-02	0.280117022	-3.648894249
**EVI2B**	1.651512898	7.102283286	2.812789417	0.008483858	0.172946727	-2.663599023
**FCER1G**	2.331024157	9.01540843	5.381054736	7.40E-06	4.24E-03	3.709362976
**FCGR3B**	1.850548765	8.746890056	2.975224984	5.66E-03	1.45E-01	-2.306009685
**FCN1**	2.923510264	8.404253082	5.298565269	9.36E-06	4.52E-03	3.495069259
**FGR**	1.203818957	8.438312939	3.374520615	2.02E-03	0.083685347	-1.384998028
**FHL2**	1.163647267	9.526287218	2.969098457	5.75E-03	1.46E-01	-2.319692182
**FN1**	1.400642184	8.446943454	2.357312505	2.50E-02	2.75E-01	-3.601391808
**FPR1**	1.017560275	7.386854892	2.193630163	0.035957976	0.320477043	-3.911519267
**LY96**	1.650033919	7.572700938	4.389714539	1.24E-04	2.14E-02	1.139319418
**TNFAIP8**	1.4284181	8.149750757	3.757345964	7.21E-04	5.18E-02	-0.457153115
**SOSTDC1**	1.185075141	8.317564616	2.423294591	2.15E-02	0.258710789	-3.472092081
**GATA3**	1.108513314	8.216325527	3.313242531	0.002370163	0.090417879	-1.529796515
**GBP1**	1.535847541	8.187850064	3.571368686	1.19E-03	6.54E-02	-0.912556817
**GBP2**	1.615897665	8.402800421	5.69733536	3.00E-06	0.00242709	4.528720381
**GEM**	1.428033586	8.127939039	4.298260127	0.000160822	0.02398204	0.904970083

**TSPAN13**	1.073986319	9.56130702	2.786070883	9.06E-03	1.79E-01	-2.721346942
**DKK3**	1.166336079	8.663287927	2.285473841	0.029346775	0.293079375	-3.739410113
**PYCARD**	1.797360553	7.828540098	4.206120996	0.000208248	0.028900934	0.669827769
**GZMA**	1.184185591	6.774406243	2.619777367	0.013547286	0.209974234	-3.073469228
**ANXA1**	1.005895067	11.53435709	3.502319732	1.44E-03	0.070172473	-1.079480367
**HBB**	3.296744388	11.46394193	4.176401448	0.000226302	0.030196825	0.594210054
**HCK**	2.6398409	7.813158766	5.349869248	8.09E-06	0.004236103	3.628368393
**HCLS1**	1.736850227	9.718789727	6.039781163	1.14E-06	1.25E-03	5.409323565
**AOAH**	1.116127075	6.591542201	2.789684088	0.008979647	0.178911318	-2.713555922
**HLX**	1.733114796	8.551807483	6.143738583	8.47E-07	0.001057373	5.674847263
**HMOX1**	1.229336838	8.989917666	2.462277565	0.019622014	0.248079788	-3.394592999
**IFI27**	1.126270095	10.77401532	3.097420419	4.15E-03	0.12217618	-2.030096345
**IL10RA**	1.758412442	7.741003398	3.760680306	7.14E-04	5.18E-02	-0.448916736
**IDO1**	1.726049947	8.191623374	2.824352429	8.25E-03	1.70E-01	-2.638510724
**CXCL10**	1.769454009	8.46969993	2.075231628	4.64E-02	3.53E-01	-4.125882705
**ISG20**	1.45907201	7.652014498	3.10186306	0.004101815	0.121683825	-2.019960048
**ITGAM**	1.287680606	7.088475734	2.706386842	0.011001205	0.19852981	-2.891676554
**ITGB2**	2.464996223	7.840432579	4.523481362	8.52E-05	0.017435001	1.483545989
**LCP1**	1.51020218	8.874061162	3.429820859	1.74E-03	0.077903959	-1.253364999
**LTF**	1.664651775	8.16718412	2.151985788	3.94E-02	3.34E-01	-3.987900664
**LYN**	1.443538636	7.934231417	4.246664501	1.86E-04	2.64E-02	0.773168949
**LYZ**	3.461839278	9.138313494	5.513669632	5.07E-06	0.003398946	4.053439457
**MEF2C**	1.06367527	8.864186566	6.495995376	3.15E-07	0.000633354	6.566914021
**MICB**	1.283842867	8.432257502	3.832196515	5.87E-04	0.046662668	-0.271701006
**MNDA**	1.944058451	7.68802875	3.405638692	0.001857523	0.079530964	-1.311036019

**MX1**	1.053339525	10.15669734	2.129124163	0.041367918	0.340011054	-4.029381896
**MX2**	1.208272091	8.90964076	2.784273318	9.10E-03	1.79E-01	-2.725220815
**NCF2**	1.949044693	7.961713901	3.649872236	9.65E-04	5.98E-02	-0.721313789
**NME1**	1.002868864	9.907840822	3.155207126	3.58E-03	1.14E-01	-1.897698368
**OAS1**	1.883982044	9.238331718	3.726464874	7.84E-04	0.053795539	-0.533320467
**CALHM2**	1.149877227	7.717842571	5.661573694	3.32E-06	0.00242709	4.436314132
**HN1**	1.223286865	7.92981769	4.344144204	1.41E-04	0.022918504	1.022435856
**CKLF**	1.029976095	9.114519974	3.542825412	1.29E-03	0.066954132	-0.981709777
**PLAC8**	2.005619689	6.904337542	3.734320791	7.68E-04	5.36E-02	-0.513963759
**TNFRSF12A**	1.505470167	8.862815372	3.067891809	0.004474091	0.126971151	-2.097285314
**CPVL**	1.210937396	7.923576346	2.523069717	1.70E-02	0.229734218	-3.27214045
**PPP1R3C**	1.081564064	9.810652913	2.790065265	0.008971252	0.178911318	-2.712733669
**GIMAP4**	1.066095495	10.51388123	4.418183879	1.15E-04	2.05E-02	1.212445487
**CYP26B1**	1.486128163	7.889780569	2.303553239	0.028182476	0.288613415	-3.704951498
**C8orf4**	1.464050458	9.91534729	5.851981163	1.93E-06	0.001946115	4.927420444
**PSMB9**	1.149756786	10.9382252	4.541310085	8.10E-05	0.017410612	1.529538356
**TIGAR**	1.062978409	7.552244523	3.320516087	0.002325317	0.089912261	-1.512669443
**PTGER2**	1.262261601	7.121536319	3.228962396	0.002954177	0.102761217	-1.727025988
**KIAA1462**	1.058038031	7.758668561	3.518102528	0.001376638	0.068968973	-1.041436211
**PTPRC**	1.520083188	6.649678491	3.534597222	1.32E-03	0.067671768	-1.001605512
**PTPRE**	1.045228874	7.86665182	2.953498611	5.98E-03	0.14881474	-2.354464495
**RAC2**	1.298734215	9.039301916	3.527947469	1.34E-03	0.068020575	-1.017671667
**ACTA2**	1.384026648	11.05579051	3.125026618	0.003865086	0.117698285	-1.966994894
**RNASE6**	1.359659579	7.485663043	2.110629291	4.30E-02	0.3451199	-4.062703594
**S100A8**	2.081494511	9.691964093	2.669478063	0.012026294	0.203903015	-2.969582677

**CCL2**	1.345476319	7.561830542	2.208988717	3.48E-02	0.317231702	-3.88308556
**CCL4**	1.868492407	8.8979264	3.486436536	1.50E-03	7.15E-02	-1.117699022
**MS4A6A**	1.501160919	8.384973582	2.583967851	1.48E-02	2.18E-01	-3.147577381
**BMP2**	1.278951698	9.788394794	2.523444183	1.70E-02	2.30E-01	-3.271380245
**TAGLN**	1.463379584	9.351012226	2.813637808	0.008466152	0.172946727	-2.661760242
**TGFBI**	1.792390756	9.264062891	2.736344105	0.010229685	0.191580633	-2.827978933
**THBD**	1.075737036	7.978789754	3.271882779	2.64E-03	9.66E-02	-1.626870472
**TIE1**	1.092945646	7.576198937	3.128567908	3.83E-03	0.117677204	-1.958880539
**TIMP1**	1.022837412	11.80032286	2.73853	0.010175414	0.191367512	-2.823315019
**TLR1**	1.020357579	5.710092232	2.467618682	0.019380496	0.24723966	-3.383911837
**TLR2**	1.485748754	7.814631146	2.744684615	0.010024049	0.189405887	-2.810171617
**C1QA**	2.063383904	7.751268347	2.483697284	0.018669798	0.242125823	-3.351667305
**TNFRSF1B**	1.180125557	7.364571534	3.312878724	0.002372428	0.090417879	-1.530652742
**C3AR1**	1.430225049	8.100369544	2.467368504	0.019391747	0.24723966	-3.38441248
**PHLDA2**	1.558843075	6.458213679	2.657620994	0.012374019	0.205343598	-2.994474172
**TYROBP**	2.98300807	8.71921773	5.200008138	1.24E-05	5.76E-03	3.238856823
**UCP2**	1.010563454	8.78271952	2.701769038	1.11E-02	0.19852981	-2.90145851
**LAPTM5**	1.762819849	9.77238142	3.80520208	6.32E-04	4.86E-02	-0.338716186
**PXDN**	1.040816746	9.163979004	2.955008217	5.96E-03	1.49E-01	-2.351103791
**CXCR4**	1.349866177	8.55377937	3.111855793	0.003998045	0.120380038	-1.997134603
**LST1**	1.573271773	7.782253429	4.647875154	5.99E-05	0.015716044	1.804910487
**TFPI2**	1.170968688	8.832996252	2.516368647	0.017298822	0.231815728	-3.285732177
**NETO2**	1.723306281	8.011145364	4.053317625	3.19E-04	0.03535287	0.282354789
**CASP1**	1.320751283	7.350815488	4.341010088	0.000142605	0.022918504	1.014405063
**GPR65**	1.138480159	6.670757888	2.838605441	0.00796032	0.168017697	-2.60750641

**IFITM1**	1.054065714	11.67585823	2.958870095	5.90E-03	0.148445424	-2.342502292
**ACTN1**	1.243479948	7.358068609	3.104465594	0.004074545	0.121471738	-2.014018802
**TNFSF10**	1.103006477	9.868178229	3.236999039	0.00289303	0.101838021	-1.70831823
**NMI**	1.021717837	9.062323387	3.710262204	8.20E-04	0.055588936	-0.573200209
**CD14**	1.928388565	9.036057353	4.099860707	0.000280191	0.033830252	0.400019583
**LY86**	1.034540159	8.018126254	3.002465142	5.28E-03	1.38E-01	-2.244995501
**CD36**	1.289686314	5.894315327	2.084666815	0.045506152	0.35070011	-4.109119423
**ADAMTS1**	1.925424756	9.230857747	4.673171181	5.57E-05	0.015189876	1.870383019
**GMFG**	1.07230973	9.241330034	4.63942951	6.13E-05	0.015754953	1.783059374
**CYTIP**	1.444773094	6.724741251	3.333208852	0.002249003	0.088451015	-1.482742523
**CD48**	2.340554521	8.382224724	5.651865741	3.42E-06	2.43E-03	4.411217068
**CD53**	2.77291088	9.682215503	5.674505668	3.20E-06	0.00242709	4.469737939
**ISG15**	1.352320169	9.675726941	2.187461347	0.036446519	0.321695779	-3.922899923
**ADGRE5**	1.071642431	8.333642554	4.331913553	0.000146302	0.022918504	0.991102239
**RASSF2**	1.312315694	7.500783902	3.410785842	0.001832353	0.079296895	-1.298774908
**RAPGEF5**	1.082850875	8.278699721	4.128261748	2.59E-04	0.032314064	0.47197854
**RIDA**	-1.404858899	9.11860268	-2.65409705	0.012479151	0.205343598	-3.001859091
**MYL9**	-1.139408367	11.6116181	-3.588297243	0.001140099	0.063729423	-0.871445487
**SLC19A2**	-1.260045943	6.687292907	-6.131792338	8.76E-07	1.06E-03	5.64438292
**LEFTY1**	-1.302868789	6.476979523	-5.39819794	7.04E-06	4.24E-03	3.753875335
**SLC17A3**	-1.598468896	9.363650571	-2.070443493	4.69E-02	3.54E-01	-4.134368143
**FTCD**	-1.207557298	8.059013021	-2.222774059	0.033728251	0.313740296	-3.85744449
**SLC27A2**	-1.496801272	9.109639244	-2.100159255	0.044024236	0.348829764	-4.08147278
**ESM1**	-1.550234226	8.417158163	-2.677732977	0.011789595	0.201911454	-2.952213881
**CHI3L1**	-1.0583532	11.87250355	-2.278145677	0.029830943	0.294023513	-3.753323769

**CYP17A1**	-1.471979338	7.113312807	-2.63447574	0.013079848	0.208071253	-3.042869644
**CYP27B1**	-2.858765526	7.768789338	-6.734243395	1.62E-07	0.000603163	7.162654885
**DEFB1**	-2.041889621	9.779850363	-2.152239441	0.039351235	0.333920768	-3.987438624
**AFM**	-1.188476527	6.753612759	-2.134319959	4.09E-02	0.338293425	-4.019982653
**DPEP1**	-1.399103764	8.820233201	-2.095174335	4.45E-02	0.348829764	-4.090384937
**TSC22D3**	-1.120316741	11.825893	-4.800217262	3.88E-05	0.012669398	2.199705199
**EPHX1**	-1.307537254	8.239811227	-4.053064339	0.000319154	0.03535287	0.281715373
**ALB**	-2.51390987	6.880747528	-3.150952781	0.00361557	0.114160332	-1.907486053
**FABP1**	-1.588334299	7.305360131	-2.244624801	0.032138723	0.306200727	-3.816570998
**FKBP5**	-1.685801422	8.329460742	-4.062403249	3.11E-04	3.52E-02	0.305297943
**DIP2C**	-1.06748798	8.452447707	-4.61651042	6.55E-05	0.015930989	1.723783688
**FBXO21**	-1.168201217	10.41650496	-4.769268614	4.24E-05	0.013126723	2.119414043
**LPIN1**	-1.092325784	8.502391361	-6.39617494	4.16E-07	0.000672562	6.315413351
**TMEM2**	-1.447358504	8.973066213	-6.578797509	2.50E-07	0.000603163	6.7747047
**G6PC**	-1.907203988	5.479825589	-3.451893251	0.0016428	0.075906542	-1.20058006
**SLC37A4**	-1.068172383	9.344609722	-3.859063753	0.000545594	0.046662668	-0.20485764
**GHR**	-1.129292393	10.48695576	-3.319613382	0.002330838	0.089912261	-1.514795939
**SLC6A16**	-1.244249347	7.273214099	-4.539298976	8.15E-05	0.017410612	1.524349133
**GSTA1**	-1.91350484	10.07005976	-2.108123171	0.043279214	0.346154442	-4.06720244
**HPD**	-2.339718954	8.94215143	-2.557555716	1.57E-02	0.22327652	-3.201830932
**IGF1**	-1.621566441	7.929502259	-2.915883501	6.57E-03	1.56E-01	-2.437907645
**APOH**	-1.379341032	6.677843793	-2.87131135	0.007340587	0.16322329	-2.536033818
**ARG2**	-1.130332916	7.23048032	-3.38141245	0.00198053	0.083284359	-1.368641971
**LPL**	-1.935374001	8.951093054	-3.488674417	0.001489349	0.071463652	-1.112318292
**MAOA**	-1.253876723	9.281233704	-3.792143524	6.55E-04	4.90E-02	-0.371081577

**MT1X**	-1.107954285	9.510941421	-2.538282184	0.016431826	0.225322304	-3.241199913
**PAH**	-1.567727035	10.72078849	-2.090445225	0.044948342	0.349907335	-4.09882534
**PCK1**	-2.158054677	10.48775084	-2.643313538	0.012806036	0.206711341	-3.024420598
**PDK4**	-2.133589177	7.967459341	-7.701722928	1.16E-08	0.000140441	9.505992077
**UPB1**	-1.219863602	7.307488702	-2.042519372	0.049763103	0.364445691	-4.183564219
**CCDC91**	-1.050755989	10.13957593	-3.89381653	0.00049585	0.045355275	-0.118188979
**PRODH2**	-1.354938654	10.04144547	-2.401046922	0.022594524	0.264101178	-3.515955012
**SDC1**	-1.330188309	8.130051544	-2.398532798	0.02272493	0.264917842	-3.520894891
**PBLD**	-1.136603119	9.008453816	-2.323771175	0.026930046	0.282222869	-3.666195874
**XYLT1**	-1.111264775	7.302998736	-4.933273142	0.0000266	0.011062871	2.545266893
**USP46**	-1.049927684	9.258699673	-3.511347888	0.001401755	0.069649353	-1.057726284
**SOWAHC**	-1.124288088	7.546180566	-4.063409292	0.000310107	0.03521262	0.307839207
**RASL11B**	-1.039689522	11.18406912	-3.544628923	0.001282143	0.066954132	-0.977346542
**ELOVL4**	-1.093221906	6.305596762	-3.472877129	0.00155348	0.073268407	-1.150271838
**UMOD**	-2.843265793	10.5106908	-2.251940074	0.031621817	0.304467159	-3.802824451
**ZBTB16**	-2.264087666	7.881084284	-3.367251839	0.002056022	0.084436766	-1.402233356
**ZNF189**	-1.418661523	9.172964451	-7.022647205	7.31E-08	0.000441538	7.874548055
**CACNB2**	-1.037091041	7.924522186	-3.338797812	0.002216168	0.088199444	-1.469549203
**LRRC2**	-1.124154605	9.271682467	-3.126568278	0.003849801	0.117677204	-1.963462952
**CALML3**	-1.126856678	6.62470469	-3.079183611	0.004346923	0.125940929	-2.071629931
**C1orf21**	-1.449631569	8.554906677	-3.850219787	0.000559011	0.046662668	-0.226876282
**USP9Y**	-1.111509719	4.868581731	-2.728999324	0.010414026	0.192851143	-2.843634102
**TLN2**	-1.042106251	7.831024086	-2.701344332	0.011136285	0.19852981	-2.902357671
**KCNK5**	-1.306860591	7.005931179	-3.754627336	0.000726222	0.051884074	-0.463866819
**DLG5**	-1.218962185	9.549684511	-4.840270591	0.0000346	0.012010107	2.303670969

**SLC22A8**	-2.181565233	7.901843046	-3.730706821	0.000775159	0.053788879	-0.522870139
**CXCL14**	-2.411662049	10.45026144	-3.073477029	0.004410755	0.126603014	-2.084601378

Table S9 Top 5 common downregulated DEGs identified using the Degree, Radiality, Stress, and Betweenness algorithms

Gene symbol	Degree		Radiality		Betweenness		Stress	
	Rank	Score	Rank	Score	Rank	Score	Rank	Score
ALB	1	11	1	3.15385	1	39.7	1	72
FABP1	2	9	2	3	4	24	4	48
PAH	3	9	3	2.92308	6	14	5	30
G6PC	4	8	4	2.92308	3	7.86667	3	28
PCK1	5	8	5	2.92308	7	6	6	20

Table S10 Gene expression matrix of the 5 hub genes after removal of batch effects

**Sample number**	**ALB**	**FABP1**	**G6PC**	**PAH**	**PCK1**
**GSM1046922**	9.086677009	8.562296554	6.304110116	11.27091202	11.20982464
**GSM1046923**	9.00681915	8.849875552	6.727468497	11.46536285	11.11406352
**GSM1046924**	8.466159249	7.859006345	6.090725383	11.31088432	11.4297326
**GSM1046925**	8.341659151	7.928733459	6.792098219	11.26330762	11.4529867
**GSM1046926**	9.44792641	8.359369571	5.629522567	11.44083754	10.95401675
**GSM920350**	4.905122533	6.571131258	5.467792127	9.36273674	10.33584067
**GSM920351**	7.294916004	7.621222651	6.853742602	10.81833052	11.19679954
**GSM920352**	7.366198069	7.307334279	5.594596921	11.00898303	11.72646246
**GSM920353**	8.678103745	8.454956019	7.200831939	11.55021769	11.51125977
**GSM920401**	7.116818884	8.61714839	6.803185522	11.55746693	11.57602057
**GSM920402**	5.821841117	7.956126009	6.125414693	10.75777312	10.65237786
**GSM920403**	4.239272926	5.445416884	4.542282562	7.273853231	7.392827783
**GSM920404**	5.455302747	5.677806088	4.346947225	9.122214946	8.993158507
**GSM920405**	4.802505902	6.745124773	5.227500953	9.942707123	9.79706942
**GSM920406**	4.35344662	5.920581291	4.497660325	9.697283706	8.377826476
**GSM920407**	4.024539609	5.053882063	4.379840554	8.554606113	9.338974354
**GSM920408**	7.11124715	7.088447987	5.161074397	10.85153343	10.58214813
**GSM920409**	3.950439181	5.842272616	4.500824835	7.724935001	7.478469495
**GSM920410**	5.917827544	6.705481894	5.189973908	10.65584171	9.06539671
**GSM920411**	7.177774545	7.57300591	5.51237597	10.80628457	10.71808981
**GSM920412**	6.415805146	7.637042499	6.196877675	10.90572131	11.89024879
**GSM920413**	5.247111005	5.518704961	4.532453725	8.735982512	9.727870464

**GSM920414**	7.079271932	7.366642881	5.456649365	10.81302989	10.73102845
**GSM920415**	6.21292432	6.350623624	5.021614121	10.34819778	9.679260484
**GSM920416**	5.820664477	8.463934574	4.906105664	10.75944483	11.44659315
**GSM920417**	4.198138658	6.149464289	4.593306341	9.120643847	7.8868673
**GSM920418**	6.296028487	6.553755948	4.568445868	10.06848966	9.714593662
**GSM920419**	5.374404617	6.342569502	4.625273769	9.83974	9.074690475
**GSM920420**	8.418928707	8.065242288	5.636532258	11.23726589	10.83149621
**GSM920421**	5.672327353	6.845375435	5.230413589	10.65559111	10.30503178
**GSM920422**	7.500689102	8.67955291	7.381703688	11.30595655	11.15003246
**GSM920423**	5.246659054	6.553314005	5.010161468	10.25971194	10.41988965
**GSM920424**	6.260793927	8.74659604	7.026247042	11.71769978	11.09072005
**GSM920425**	5.202506378	7.076396551	6.613251599	10.2974381	11.07496647
**GSM920426**	7.573723464	8.198470392	5.63381397	11.21465478	10.61421313
**GSM920427**	5.923149905	7.133584147	5.15517606	10.33256468	10.28578093
**GSM2462533**	5.779569931	9.040150501	5.323333783	10.16593748	10.08411516
**GSM2462534**	8.935133193	8.254389666	6.240635501	11.72250754	12.14085011
**GSM2462535**	5.365326384	6.432159624	5.176737057	8.229916576	9.858233313
**GSM2462536**	7.408602136	6.994188068	5.856794414	11.40641719	11.46497884
**GSM2462537**	5.697636145	8.041638776	5.961239154	10.84553065	10.68660557
**GSM2462538**	7.983938743	8.54180657	6.887911607	11.87309232	11.78568824
**GSM2462539**	6.916733156	9.264541069	6.943290196	12.48636491	12.6813464
**GSM2462540**	9.009639955	8.654835808	7.46385631	11.92613546	11.34461542
**GSM2462541**	7.314974044	6.950240917	6.425157947	11.22040242	11.39806275
**GSM2462542**	7.274604117	9.269423204	6.569221467	11.49164588	11.62291701
**GSM2462543**	7.17851593	8.91734774	6.853383365	11.97762108	12.10601089

**GSM2462544**	7.314083198	7.246285482	5.949092535	9.761724965	10.26965144
**GSM2462545**	6.699815614	8.224338231	5.517959252	10.96168069	10.82818136
**GSM2462546**	9.325924395	7.926042914	6.635151534	11.86874635	11.60016749
**GSM2462547**	6.137542864	9.182555817	5.698766328	12.32673467	11.90695363
**GSM2462548**	6.060852183	8.043971469	5.707248667	11.4346026	11.03790127
**GSM2462549**	8.090380009	8.503621766	7.281403004	11.36464693	11.62823563
**GSM2462550**	5.585923447	7.838153899	6.404541762	11.09725503	11.8730813
**GSM2462551**	9.327616862	8.787466279	6.031413939	11.74019755	11.98770547
**GSM2462552**	6.441586312	9.408476843	6.304797292	10.05263865	10.86073674
**GSM2462553**	8.702142863	7.040662093	7.080986305	11.01424566	11.0473531
**GSM2462554**	6.00214907	7.52255558	5.463348805	9.634816544	10.36872513
**GSM2462555**	4.428720406	5.065884476	3.828648705	6.463964545	6.515112456
**GSM2462556**	5.082639858	6.392515631	4.71422219	8.674611676	9.503126766
**GSM2462557**	4.457380449	7.122383018	5.209877732	10.33355607	8.832082908
**GSM2462558**	4.400024957	4.936414044	5.4605417	9.131378048	9.584858204
**GSM2462559**	3.950912353	4.139392842	4.641888858	7.233927798	7.291532181
**GSM2462560**	4.624540895	6.853047461	4.775407995	10.60573968	8.870586442
**GSM2462561**	3.631730183	5.767496535	4.279162082	9.051682686	8.005089712
**GSM2462562**	4.11348402	6.944596096	4.70038251	10.80803167	9.256581624
**GSM2462563**	5.857061502	6.258970586	5.058274197	9.950769845	9.70245504
**GSM2462564**	6.827508996	7.249738826	6.405998198	10.41872841	10.52089723
**GSM2462565**	4.325870851	5.625037033	4.314567367	8.960774639	9.273460621
**GSM2462566**	5.332490389	5.596616501	4.610238679	8.572599819	8.384744331
**GSM2462567**	5.111571828	6.10623798	4.81184927	10.61975362	9.664936748
**GSM2462568**	4.938420475	7.17360315	5.052725269	10.12971168	8.80301823

**GSM2462569**	4.23468586	6.386023739	4.995762894	8.636776232	9.048964886
**GSM2462570**	2.766173118	5.366086918	3.467309871	9.028003352	7.903011164
**GSM2462571**	5.610934397	6.312489048	4.834294756	9.29742158	8.944500864
**GSM2462572**	5.597507969	6.676005944	5.504657809	10.19099462	9.604965355
**GSM2462573**	5.885388716	4.063781857	4.335921517	8.86680789	8.500000861
**GSM2462574**	4.962522897	6.838333651	5.972875248	9.369267807	9.833798701
**GSM2810714**	9.424858071	8.781813923	7.525505479	12.19131219	12.77521288
**GSM2810715**	7.845361949	8.543882091	6.827727758	11.49829105	11.8465531
**GSM2810716**	8.669808855	8.509493433	6.94553036	11.72941574	11.81546315
**GSM2810679**	7.689772925	7.849547986	5.557756708	11.04389637	10.92617908
**GSM2810680**	7.643832794	8.814840015	6.850984292	11.85226578	11.85274504
**GSM2810681**	6.266965626	8.175825197	6.18183396	10.99233608	10.85424962
**GSM2810682**	4.600749073	5.539373628	4.674354899	7.353990749	7.512297388
**GSM2810683**	5.868758734	5.833914703	4.351843282	9.255566101	9.161125445
**GSM2810684**	5.220949886	6.915737509	5.316083308	10.14014911	9.99492308
**GSM2810685**	4.740693091	6.101047581	4.540687575	9.867714027	8.516520447
**GSM2810686**	4.361635005	5.195805274	4.383660671	8.701128583	9.537621592
**GSM2810687**	7.628268284	7.22808786	5.205072893	11.09073973	10.84815337
**GSM2810688**	4.321557455	5.946382881	4.620407381	7.782479876	7.603619634
**GSM2810689**	6.389760555	6.835649997	5.209227622	10.9051551	9.244703337
**GSM2810690**	9.129150472	8.37008051	5.762079298	11.59037708	11.18035252
**GSM2810691**	6.925198146	7.843337553	6.272678349	11.13553274	12.18789041
**GSM2810692**	5.705346303	5.673701132	4.555622213	8.854357476	9.943423121
**GSM2810693**	7.598322272	7.557620887	5.620671049	11.07641603	10.90381969
**GSM2810694**	6.666613291	6.435210643	5.04643189	10.57366242	9.88811301

**GSM2810695**	6.244406176	8.735625621	4.96213362	11.03339388	11.73656998
**GSM2810696**	4.545278856	6.343414027	4.692603904	9.261971501	7.994783978
**GSM2810697**	6.772373091	6.664004317	4.616649737	10.2537068	9.906434523
**GSM2810698**	5.930083391	6.523363292	4.806608886	10.07390325	9.327265778
**GSM2810699**	6.288745448	7.16100389	5.337807469	10.9253369	10.59272081
**GSM2810700**	8.175973701	9.060023029	7.481414006	11.64342698	11.50449364
**GSM2810701**	5.863795166	6.905416071	5.090765878	10.56062858	10.73296629
**GSM2810702**	6.916412415	9.095088488	7.128951386	12.07280826	11.42102385
**GSM2810703**	5.715933467	7.248072217	6.702700113	10.5338017	11.28591949
**GSM2810704**	8.145652134	8.455868742	5.67819564	11.53106638	10.8689046
**GSM2810705**	6.464912986	7.360086805	5.188558561	10.59293454	10.53861939

Table S11 GS and MM values with IgAN clinical traits in the turquoise module

Gene symbol	GS.ACR	p.GS.ACR	MMturquoise	p.MMturquoise
**TMEM52B**	0.078712576	0.76395898	0.828870599	3.92E-05
**SLC27A2**	0.322622782	0.206592502	0.928657046	7.49E-08
**HLA-DQA1**	0.290049505	0.258769435	0.567018467	0.017616268
**CYP24A1**	0.506794204	0.037878871	0.596209265	0.011537691
**SLC13A1**	0.259833767	0.313859573	0.88657992	2.14E-06
**SLC10A2**	0.230835596	0.372720117	0.847588863	1.74E-05
**SLC17A3**	0.459033932	0.063812737	0.841322187	2.31E-05
**TMED6**	0.023646192	0.928222549	0.692449415	0.002064995
**CYP4F2**	0.162746629	0.53256642	0.429340445	0.085461946
**MT1G**	0.316360021	0.216045779	0.746942208	0.00057033
**UMOD**	0.152858944	0.558072573	0.626829722	0.007084684
**RBP4**	-0.349766482	0.168755354	0.262858821	0.308054946
**CXCL11**	0.313429319	0.220563704	0.412669753	0.099720406
**SOSTDC1**	0.319120845	0.21184472	-0.074881946	0.775161646
**SERPINA5**	-0.056815902	0.82852897	0.69344408	0.002021991
**ANPEP**	0.431399385	0.08380935	0.860409371	9.37E-06
**OTOGL**	0.522303467	0.031490956	0.565955903	0.017877271
**PLPPR1**	0.596197522	0.011539745	0.76013558	0.000398065
**SLC22A8**	0.425316294	0.088759758	0.938384469	2.57E-08
**GGT6**	-0.027194437	0.917484173	0.51822508	0.033084064
**DNER**	0.118159446	0.651511656	0.497413152	0.042194377
**AOC1**	0.307070593	0.230573448	0.796618929	0.000129543

**GBA3**	0.349807725	0.168701689	0.920318253	1.67E-07
**PIPOX**	0.226015884	0.383059502	0.924527585	1.13E-07
**HPD**	0.458198914	0.064359563	0.943393416	1.38E-08
**BBOX1**	0.184662974	0.477996162	0.903638474	6.63E-07
**SLC6A19**	0.46178133	0.062037779	0.925248428	1.05E-07
**TUBAL3**	-0.091788074	0.726063886	0.3323121	0.192504621
**AGXT2**	0.351942748	0.165939279	0.950455389	5.18E-09
**AKR1C2**	0.323434474	0.205387324	-0.383570069	0.128544001
**OMD**	-0.183936672	0.479759435	-0.66826268	0.003364979
**CXCL10**	0.332451579	0.192306568	0.535394565	0.026772256
**CTXN3**	0.492925626	0.04438572	0.828170869	4.03E-05
**SLCO4C1**	0.32980656	0.196085233	0.941553409	1.74E-08
**SLC12A1**	-0.054829823	0.834443741	0.48807285	0.046850959
**DIO1**	0.263279186	0.307253398	0.881077076	3.00E-06
**MGAM**	0.585274122	0.01358023	0.822973881	4.96E-05
**FABP1**	0.304226886	0.235141953	0.76290979	0.00036803
**KMO**	0.336499137	0.186617605	0.843562042	2.09E-05
**MIOX**	0.289361572	0.259952032	0.938925239	2.41E-08
**SLC22A12**	0.46317048	0.06115436	0.751046068	0.000511156
**TMEM27**	0.378300354	0.134324398	0.970511091	1.12E-10
**CUBN**	0.416810271	0.096030806	0.863513759	7.99E-06
**DEFB1**	0.081887565	0.75470707	0.692267808	0.002072927
**FREM2**	0.25904818	0.315377466	0.9160619	2.44E-07
**XPNPEP2**	0.394698759	0.116910393	0.925628866	1.01E-07

**DPYS**	0.444173317	0.074070088	0.95283276	3.61E-09
**SLC47A1**	0.373924301	0.139259334	0.792763388	0.00014735
**CAPN3**	0.379703269	0.132768278	0.72824686	0.000916793
**PLG**	0.20864245	0.421609223	0.913892145	2.94E-07
**FAM150B**	0.150238803	0.564919349	0.290781548	0.257514681
**GLYAT**	0.309109855	0.227332347	0.953635315	3.18E-09
**LOC102724873**	-0.09745785	0.709811279	-0.244453922	0.344356749
**SLC5A12**	0.407940633	0.104057048	0.977265452	1.63E-11
**FAM151A**	0.138795129	0.595238206	0.827393392	4.16E-05
**HAO2**	0.329031509	0.197201629	0.906976804	5.14E-07
**UGT3A1**	0.310269731	0.225501941	0.812236299	7.47E-05
**AFM**	0.118729737	0.649930532	0.844214153	2.03E-05
**FMO1**	0.360121545	0.155639566	0.91541185	2.58E-07
**FBP1**	0.166112418	0.524006619	0.851485737	1.45E-05
**LRRTM2**	-0.37926324	0.133255009	-0.477716937	0.052454959
**FOLH1**	0.289765245	0.259257688	0.707672843	0.001482865
**ALDOB**	0.390748089	0.120952074	0.946341129	9.31E-09
**TMEM174**	0.550091334	0.022146484	0.925994471	9.78E-08
**LOC100505985**	0.408762373	0.10329405	0.959052602	1.27E-09
**MPP3**	-0.039282173	0.881013537	-0.367678683	0.146516897
**TMEM207**	-0.026889247	0.918407292	0.430521191	0.084511369
**SLC16A9**	0.439074173	0.077852991	0.908105267	4.70E-07
**SLC13A3**	0.392030684	0.11962936	0.922283517	1.40E-07
**GLYATL1**	0.164561403	0.527943262	0.895780068	1.17E-06

**IYD**	0.552661759	0.021405752	0.740656554	0.000671923
**SLC2A2**	0.256495291	0.320339845	0.817157692	6.21E-05
**PAQR5**	0.171089285	0.511466626	0.659308752	0.003988948
**C9orf66**	0.510644242	0.036208437	0.943193448	1.42E-08
**PARD3-AS1**	-0.564423286	0.018259061	-0.291449673	0.256372792
**SMIM24**	0.372784089	0.140565413	0.93756288	2.83E-08
**CLRN3**	0.273835709	0.287531126	0.853854706	1.30E-05
**SLC34A1**	0.401896764	0.109792379	0.903239294	6.83E-07
**SLC22A2**	0.398653622	0.112960466	0.728824107	0.000903965
**BORA**	0.052108394	0.842562059	-0.050491971	0.847391182
**SLC4A4**	0.334907851	0.188840759	0.831215857	3.56E-05
**KCNJ1**	0.101444579	0.698453146	0.715714958	0.001234689
**NPTX2**	0.072338494	0.782623342	-0.61728808	0.008290479
**CYP8B1**	0.314715893	0.218572932	0.686556129	0.002335427
**DAO**	0.47885986	0.051813005	0.933308285	4.58E-08
**BHMT**	0.454622352	0.06674096	0.927012336	8.84E-08
**NAT8**	0.359138183	0.156854361	0.962954328	6.07E-10
**APOD**	0.172749652	0.507314565	-0.401211367	0.110456612
**SLC23A3**	0.122989225	0.638166177	0.782025292	0.000208102
**ASPSCR1**	-0.112331285	0.667749699	-0.371734159	0.141775511
**ACMSD**	0.30321356	0.236783659	0.878662373	3.46E-06
**DMGDH**	0.271542583	0.291748637	0.822153901	5.13E-05
**SLC3A1**	0.374842501	0.138213662	0.903767665	6.56E-07
**ACSM5**	0.423306039	0.090441108	0.764385197	0.000352842

**GDA**	0.415002779	0.097629225	0.621395219	0.007752816
**PAH**	0.405440323	0.106403263	0.880756789	3.06E-06
**RNF128**	0.235714978	0.362412409	0.827211154	4.19E-05
**ACPP**	0.000287369	0.999126641	0.267729326	0.298843913
**KCNJ15**	0.489513164	0.046108771	0.963589611	5.34E-10
**DPP4**	0.036758692	0.888610986	0.596701401	0.011451858
**PCK1**	0.277378063	0.281088884	0.868644474	6.09E-06
**CX3CR1**	0.507217105	0.037692572	0.222644331	0.390384747
**UBD**	0.218903356	0.398601077	0.574440045	0.015875487
**UNC80**	0.151350214	0.562010668	0.380097711	0.132333021
**LRRC19**	0.274511009	0.286296178	0.905393007	5.80E-07
**DOK7**	-0.20324581	0.433983822	-0.408920951	0.103147267
**LMO3**	0.03993733	0.879042633	-0.392031419	0.119628605
**RNF183**	0.364327698	0.150515697	0.561171642	0.019090204
**PAX8-AS1**	0.076055065	0.771726299	-0.458475067	0.064178337
**ENC1**	0.273955517	0.287311794	-0.131439283	0.615069992
**ALB**	0.291981798	0.255465596	0.519027584	0.032765816
**SLITRK2**	-0.071058983	0.786383855	-0.459187223	0.063712726
**TINAG**	0.380257424	0.132157064	0.847539966	1.75E-05
**SLC44A3**	0.385493275	0.126478215	0.613572033	0.008802251
**SUSD2**	0.60099548	0.010724229	0.829623709	3.80E-05
**PKI55**	-0.303653301	0.236070339	-0.343927918	0.176468449
**PSAT1**	0.408096153	0.103912339	0.746503815	0.000576974
**SLC7A9**	0.166213846	0.523749655	0.818867674	5.82E-05

**CDH10**	-0.069369421	0.791356354	-0.366127987	0.148358225
**LOC100506922**	-0.089052714	0.733945063	-0.472735025	0.055322873
**C1D**	-0.051070185	0.845663147	-0.478876541	0.05180368
**ERICH4**	0.29015265	0.258592411	0.783450678	0.000198999
**KIF4A**	0.060663605	0.81709508	0.287310214	0.263498299
**HLA-DQB1**	0.269087076	0.296305841	0.619930751	0.007941226
**ENPP6**	0.06860833	0.793598798	0.778283431	0.000233671
**LGALS2**	0.289617935	0.259510937	0.840196427	2.43E-05
**RALYL**	0.036732622	0.888689522	0.526287752	0.029992097
**ELOVL7**	0.192749205	0.458581401	0.522064825	0.031582521
**CMBL**	0.400283709	0.111360145	0.89110474	1.60E-06
**RIDA**	0.452497152	0.068186387	0.931027016	5.85E-08
**NPL**	0.540388092	0.025123933	0.662284311	0.003771979
**CLHC1**	-0.473721541	0.054745896	-0.389060839	0.122707657
**SLC22A11**	0.310492455	0.225151542	0.923906326	1.20E-07
**ELOVL4**	-0.288499901	0.261438017	-0.559650206	0.019489107
**CXCL9**	0.208859002	0.421116569	0.556119761	0.02043984
**ADAM1A**	-0.384286257	0.127771993	-0.233697587	0.366654591
**IGSF11**	0.182804734	0.482513809	0.70135394	0.00170544
**GATM**	0.387455114	0.124394889	0.908710507	4.48E-07
**FGG**	-0.271758481	0.291349979	-0.403343324	0.108399769
**SLC1A1**	0.198811656	0.444290307	0.771677714	0.000285225
**LOC100505715**	-0.385530789	0.126438151	-0.524069247	0.030819754
**LOC102723864**	0.085472249	0.744299139	0.543296241	0.024200857

**SLC7A13**	-0.277258217	0.281305394	0.181727004	0.48514341
**PBLD**	0.32189651	0.207674739	0.950510189	5.14E-09
**SLC47A2**	0.390015774	0.121711873	0.913740671	2.98E-07
**SUGCT**	0.228275604	0.378192447	0.784020446	0.000195455
**RSPO3**	-0.381625464	0.130656524	-0.571122135	0.016636174
**CRYM**	0.243359907	0.346588452	0.900132796	8.57E-07
**PPP1R14C**	0.36987241	0.143938852	0.176020099	0.499182875
**METTL7B**	0.139396347	0.593628921	0.693707837	0.002010712
**PROC**	0.196249533	0.450302046	0.419689201	0.09352373
**AZGP1**	0.319687683	0.210988771	0.900058553	8.62E-07
**ACSM3**	0.329203665	0.196953294	0.779560429	0.000224665
**USP43**	0.028487027	0.913575545	-0.10337158	0.692984591
**ACE2**	0.149488394	0.566886909	0.83274057	3.34E-05
**HIST1H3I**	0.297685644	0.245867016	0.306319524	0.231774531
**OGDHL**	0.239077695	0.355402824	0.893798045	1.33E-06
**FABP3**	0.304945311	0.233982405	0.713637573	0.001295247
**TMEM176A**	0.374667512	0.138412526	0.769250481	0.000306409
**AGT**	0.183858945	0.479948323	0.685205458	0.002401314
**SMTNL2**	0.162009666	0.534449065	0.500198674	0.04087597
**ECT2**	0.366849009	0.147500111	0.49204104	0.044827628
**PRORSD1P**	-0.021520338	0.934661947	-0.41590925	0.096825245
**MRLN**	0.381123209	0.131206043	0.499506692	0.041200534
**FAM69A**	-0.115707929	0.658324383	-0.443075054	0.074873254
**GGTLC1**	0.361526894	0.153914593	0.723751565	0.001021934

**THY1**	0.457575373	0.064770151	0.632145227	0.006476667
**ACSF2**	0.192307993	0.459630398	0.599753112	0.010930859
**PCDHB14**	-0.264645813	0.304656082	-0.495231564	0.043249214
**APOE**	0.357192346	0.159277079	0.523626386	0.030987051
**ADAM32**	0.011101065	0.966271154	-0.00797767	0.975757972
**KCNJ10**	0.011211829	0.965934797	0.685921945	0.002366176
**GLDC**	0.323415368	0.20541564	0.775610261	0.000253504
**CLCN5**	0.054470737	0.835514047	0.858954111	1.01E-05
**AKR7A3**	-0.051332312	0.844879981	0.582580842	0.014124638
**SLC22A6**	0.488768952	0.046491146	0.952353054	3.89E-09
**LOC100506388**	0.096855778	0.711531672	0.36413761	0.150744741
**PTTG1**	0.271166742	0.292443414	0.360979789	0.154584574
**ZFPM2**	-0.282761699	0.271467575	-0.354654492	0.162474816
**VNN1**	0.3767635	0.136043525	0.431597954	0.083651203
**CD99P1**	-0.424087529	0.089784778	-0.678654302	0.002742807
**ZNF185**	0.049634429	0.849955217	-0.53081222	0.028357196
**MGST1**	0.263697565	0.306456866	0.69144919	0.002108991
**GBP3**	0.064451601	0.805872568	0.391221481	0.120462688
**ITIH2**	0.419624957	0.093579159	0.336650701	0.186406765
**SPP1**	0.25862206	0.316202607	0.730471801	0.000868162
**IL17RB**	0.063372506	0.809066	0.810861329	7.86E-05
**CLDN2**	0.305123186	0.233695873	0.856996986	1.11E-05
**DMD**	0.276888072	0.281974723	-0.406143229	0.10573992
**IMPA2**	0.418650647	0.094422659	0.89534391	1.20E-06

**SLC7A7**	0.406736503	0.105182329	0.839585238	2.50E-05
**ADH6**	0.281766323	0.27323103	0.715260115	0.001247744
**AGMAT**	0.353058725	0.164507561	0.952293823	3.92E-09
**RAVER2**	0.31008025	0.225800316	0.383856577	0.128234774
**EHHADH**	0.247143174	0.338905922	0.753308518	0.000480779
**VEPH1**	0.248725281	0.335722477	0.655988285	0.004242902
**BTC**	-0.424501598	0.089438419	-0.467686528	0.058346783
**DUSP9**	0.255170304	0.322933304	0.794668759	0.00013831
**IGFBP7-AS1**	-0.357611303	0.158753316	-0.281799621	0.273171923
**ZMYND12**	-0.133561437	0.609321838	0.211649276	0.414795882
**LRP2**	0.371707967	0.14180579	0.926695213	9.12E-08
**NAT8B**	0.27929308	0.277643033	0.815090035	6.72E-05
**SHMT1**	0.313573913	0.220339387	0.865610523	7.16E-06
**ALDH8A1**	0.298113911	0.245155591	0.910663691	3.84E-07
**LINC00645**	0.146361117	0.575118071	0.750397354	0.000520154
**BHMT2**	0.294166894	0.251761302	0.911765334	3.51E-07
**RNF212B**	0.51495466	0.034405549	0.522675535	0.031348603
**FCAMR**	0.547901779	0.022793048	0.678826081	0.002733373
**TUBB4A**	-0.108308745	0.679039409	0.414623807	0.09796676
**FLJ22763**	0.004824531	0.985338245	0.700144466	0.001751017
**EPSTI1**	0.288384925	0.261636694	0.756574682	0.000439585
**SEPT7P2**	-0.031767287	0.903665053	-0.586029615	0.013430534
**PPP1R3C**	-0.190423306	0.464124818	-0.337119246	0.185755972
**TLR8**	0.241111708	0.351200405	0.521094664	0.03195687

**PLS1**	0.176377763	0.498297356	0.678068935	0.002775154
**CLDN10**	-0.047124765	0.857467154	0.485374912	0.048265349
**HSD17B2**	0.216308123	0.404355326	0.163873949	0.5296924
**WDR72**	0.088774976	0.734746707	0.861342761	8.94E-06
**USP6NL-IT1**	-0.464806041	0.06012623	-0.314522242	0.218871834
**MUC13**	0.318985556	0.212049344	0.429515672	0.08532039
**MAN2B1**	-0.047688955	0.855777389	-0.326152822	0.201384567
**UGT2A3**	0.286337218	0.265190755	0.623664277	0.007467977
**FOXQ1**	0.167982888	0.519277178	0.529970701	0.028655953
**ZNF417**	-0.410619759	0.101584122	-0.569021269	0.017132434
**ANKRD9**	-0.1374198	0.598926245	0.086989175	0.739907287
**PTGDS**	-0.030250987	0.908244591	-0.638309625	0.005824825
**KCNJ16**	0.153628088	0.55606959	0.843287848	2.12E-05
**LINC00052**	-0.064240417	0.806497312	-0.536458548	0.026414233
**ESRRG**	-0.28075817	0.275024253	0.66115638	0.003853067
**CCDC173**	-0.199268757	0.443222105	-0.174121399	0.503896264
**ZNF100**	-0.495076084	0.043325145	-0.476490552	0.053150388
**LONRF3**	-0.514599591	0.034551413	-0.305251479	0.233489351
**FAM182B**	-0.260847368	0.311907492	0.03214996	0.902509749
**ASS1**	0.441320691	0.076169406	0.906063737	5.51E-07
**ABCB1**	0.450231774	0.069752378	0.537150078	0.02618353
**APOM**	0.358985225	0.157043894	0.936336538	3.26E-08
**AIFM1**	0.305075682	0.233772373	0.620924599	0.007812969
**DYNC1I2**	0.090189655	0.730666183	0.458543642	0.064133393

**CD83**	0.083984432	0.748613967	0.431332965	0.083862297
**PNP**	0.347333318	0.171941665	0.698854946	0.001800707
**MRO**	0.452606406	0.068111523	0.842509582	2.19E-05
**SEC31B**	-0.116614315	0.655802554	-0.488669201	0.046542579
**CLEC3B**	0.385389995	0.12658856	0.493177666	0.044260414
**GSTA1**	0.43370663	0.081985106	0.914351752	2.83E-07
**UBE2C**	0.171599599	0.510188799	0.287134232	0.263803911
**SLC39A4**	0.016666599	0.949378102	0.696258342	0.001904276
**PLA2G10**	-0.116040729	0.65739803	-0.345780757	0.173995722
**GCNT2**	-0.113758227	0.66376081	0.567723083	0.017444842
**KCNK5**	0.289020103	0.260540281	0.665957154	0.003517477
**NUPR1**	0.09860777	0.706529121	-0.514040657	0.034781984
**LINC00942**	-0.318385467	0.212958516	-0.329013777	0.197227219
**DHRS11**	0.329213641	0.196938909	0.49494698	0.043388271
**CMAHP**	-0.335214727	0.188410674	-0.387227738	0.124635109
**RORC**	0.415308248	0.097357762	0.717268802	0.001190935
**NQO2**	0.452934797	0.067886863	0.468443114	0.057885958
**LDLR**	-0.142011305	0.586650314	-0.468220327	0.058021372
**ALMS1-IT1**	-0.38246214	0.129744667	-0.366379534	0.148058462
**PDZK1IP1**	0.304841174	0.234150259	0.582474471	0.014146488
**CYS1**	0.191809565	0.460816872	0.637031374	0.005955448
**OLR1**	-0.002813369	0.991449882	0.533505397	0.027417169
**TYRP1**	-0.224093287	0.387227364	0.280348642	0.275754739
**FAM201A**	-0.36401452	0.150893184	-0.36634459	0.148100079

**USH1C**	0.293435742	0.252997035	0.712026213	0.001343886
**PRRG4**	0.194099173	0.455379292	0.547437682	0.022931957
**GPAT3**	0.549878667	0.02220865	0.876045848	4.03E-06
**APOBEC3A**	-0.023195792	0.929586511	0.58688238	0.013263134
**GDF15**	0.216305198	0.404361836	0.427009658	0.087361043
**ANGPTL3**	0.593213407	0.012071263	0.810071646	8.09E-05
**SLC5A10**	0.542279078	0.024520674	0.746858847	0.000571588
**BEGAIN**	0.335484567	0.188033029	0.541568126	0.024746149
**SPC25**	-0.033530626	0.898343025	-0.428301316	0.086304889
**SH3GL2**	0.193901355	0.455847808	0.778784262	0.000230104
**HGD**	0.056477057	0.829537484	0.85060288	1.51E-05
**ZNF793-AS1**	-0.489050322	0.046346299	-0.62377058	0.007454841
**LOC100128361**	-0.006335376	0.980747487	-0.427691199	0.086802609
**LACTB2**	0.176094673	0.498998181	0.664616307	0.003608718
**SEPT7**	-0.057295511	0.827101935	-0.567055077	0.017607328
**DRAIC**	0.488305148	0.046730653	0.514612276	0.034546194
**ALDH6A1**	0.246757979	0.33968361	0.867761111	6.39E-06
**C1QL1**	0.030235274	0.908292062	-0.387571218	0.124272351
**KLHL12**	0.186248612	0.474157706	0.195078335	0.453063808
**TFAP2B**	0.241368809	0.350671233	0.634864669	0.00618224
**LINC00551**	0.391902743	0.119760845	0.40388862	0.107878072
**ITIH3**	0.116305347	0.656661801	-0.165170375	0.526396016
**CYP4A11**	0.08151347	0.75579557	0.53773268	0.025990377
**PECR**	0.005101208	0.984497513	0.764701641	0.000349654

**RANBP3L**	-0.035179317	0.893370722	0.178569438	0.492887509
**PLP2**	0.173088669	0.506468745	-0.554602383	0.020859407
**RGS18**	0.262319039	0.309086013	0.466074295	0.059337862
**SLC17A1**	0.562442955	0.018761799	0.779672934	0.000223886
**ANK2**	0.530301127	0.028538357	0.552322456	0.021502409
**LOC284825**	-0.013295986	0.959606917	0.564613452	0.018211345
**ANOS1**	-0.229389824	0.375805228	-0.372809306	0.140536436
**RBM47**	0.064833172	0.804744054	0.755375796	0.00045435
**ART4**	-0.200174737	0.441108804	-0.273995332	0.287238927
**CNDP1**	-0.183690989	0.480356607	0.463811859	0.060749642
**UPP2**	-0.056543984	0.829338267	0.60943112	0.009401924
**ACP2**	0.350660508	0.167594631	0.454395758	0.06689399
**PDPR**	-0.232727681	0.368703942	-0.421023078	0.092378219
**FTCD**	0.255360907	0.322559476	0.603692283	0.010286376
**PCDH19**	0.205525422	0.428733859	-0.082808486	0.752029339
**PKNOX2**	-0.179298013	0.491095394	-0.463659505	0.060845599
**G6PC**	0.181924618	0.484660721	0.734353635	0.000788429
**SFTA2**	0.041244369	0.875112691	0.419900611	0.093341499
**MAP7D2**	0.054172619	0.836402837	0.555850628	0.020513773
**HRG**	0.3226119	0.20660869	0.801054954	0.000111326
**LOC101927681**	0.184584756	0.478185903	0.629466452	0.006777605
**PIGZ**	0.015453974	0.953057277	-0.394164448	0.117451383
**MTTP**	0.417297117	0.095603498	0.790062785	0.00016101
**ISG15**	0.309200853	0.227188401	0.484784047	0.048579346

**WSCD2**	0.138185588	0.596871584	0.375655928	0.137291845
**TFCP2L1**	0.128531223	0.622981342	0.69766138	0.001847724
**LOC100506098**	0.46172923	0.062071094	0.699903732	0.001760207
**HNF4A**	0.456449117	0.065516662	0.78232651	0.00020615
**KCNQ1OT1**	-0.440354268	0.076890373	-0.362342669	0.152919273
**WDR49**	-0.493939768	0.043883145	-0.487385308	0.047208401
**CA4**	0.125150702	0.632227218	0.616430524	0.008406414
**DDX60L**	0.013433302	0.959190076	0.453029799	0.067821971
**MAP2K6**	0.082335066	0.753405554	0.399505195	0.112122433
**APOC1**	0.249820466	0.333528932	0.258854971	0.31575144
**HACD1**	-0.616885743	0.008344712	-0.426416372	0.087849272
**RAB11B-AS1**	-0.122727294	0.638887293	-0.509492808	0.03670201
**AK4**	0.224717839	0.385870735	0.826346161	4.34E-05
**LOC389906**	-0.226785343	0.381398373	-0.557789862	0.019985684
**RGN**	0.092337052	0.724485256	0.510947616	0.036079239
**UBE2T**	0.170512735	0.512912104	-0.104004269	0.691192216
**VEGFC**	0.34157989	0.179635577	0.698292839	0.001822726
**PSMB9**	0.270830389	0.293066039	0.424813673	0.089178013
**SDC1**	0.369985706	0.143806559	0.880607907	3.09E-06
**ADAM28**	-0.170247128	0.513578653	-0.360230957	0.155504801
**PANK1**	-0.269037606	0.296398089	0.56117024	0.019090569
**PLA2G4C**	0.477597297	0.0525225	0.57476967	0.015801435
**FGA**	-0.197440908	0.447501496	-0.341203232	0.180147128
**NHS**	0.027000246	0.918071536	0.526307351	0.029984863

**PPRC1**	-0.555059722	0.020732249	-0.251183201	0.330811111
**FRG1CP**	-0.410028972	0.102125802	-0.607670318	0.009666583
**LOC100506498**	-0.438626881	0.078191412	-0.584265843	0.013782063
**ZNF440**	-0.271715151	0.291429962	-0.275840725	0.283873869
**CCL8**	0.124336275	0.634462498	0.680761748	0.002628888
**PIR**	0.154655181	0.553399774	0.399017958	0.112601384
**EPB41L3**	0.330929617	0.194474935	0.772272121	0.000280228
**TUBA3FP**	-0.493603495	0.044049312	-0.377510121	0.135206465
**IGSF6**	0.267388536	0.299482992	0.625387535	0.007257307
**CALML4**	0.100925871	0.699927577	0.730270296	0.000872477
**FARSB**	-0.618689965	0.00810371	-0.357639444	0.158718178
**GJB1**	0.21650887	0.403908642	0.601022342	0.010719796
**SPATA1**	-0.120648553	0.644621036	-0.630260458	0.006687258
**ZNF385B**	0.185312045	0.476423078	0.684060225	0.002458362
**MCM8**	-0.380251232	0.132163883	-0.488376999	0.046693489
**LOC101929289**	-0.258491615	0.316455456	-0.294152997	0.251784755
**ZNF75A**	-0.201771825	0.437396047	-0.126756593	0.627828503
**DARS2**	0.01731612	0.947407809	-0.304945876	0.233981494
**ABCC2**	0.173026122	0.506624745	0.639429861	0.005712256
**SLC25A13**	0.16787437	0.519551023	0.599440564	0.010983336
**SLC6A9**	-0.090774545	0.728981104	0.504826141	0.038755088
**PAN3-AS1**	-0.273911066	0.287393158	-0.341350011	0.179947667
**SULT1C2**	-0.015178686	0.953892648	0.797499244	0.000125742
**ARSE**	0.274512747	0.286293004	0.601051075	0.010715056

**AURKA**	0.605196746	0.010048365	0.41281559	0.099588758
**UGT1A8**	0.381634186	0.130646996	0.357247041	0.159208635
**LOC101927402**	-0.018629112	0.943425798	0.508919938	0.036949477
**GRIK1-AS2**	0.146639103	0.574384352	-0.246943882	0.339308154
**MT1X**	0.315438119	0.217460483	0.28701939	0.264003465
**KIRREL3**	0.300162578	0.241770313	0.402950916	0.108776298
**HELLS**	0.232297242	0.369615472	0.409992134	0.102159646
**FAM26D**	0.401699178	0.109983573	-0.246180716	0.340850993
**EOGT**	-0.167554244	0.520359248	-0.445309112	0.073246122
**OAS1**	0.172394519	0.508201307	0.571490145	0.016550418
**C2CD4A**	-0.433530467	0.082123365	-0.357406649	0.15900902
**STEAP1**	0.227511495	0.379834376	0.581359045	0.014377214
**GOLGA8A**	-0.346567103	0.172953336	-0.098760911	0.706092385
**PNO1**	-0.347051015	0.172313941	-0.304608012	0.234526358
**DUS3L**	-0.039623983	0.87998519	-0.383397796	0.128730185
**RASL11A**	0.165563435	0.525398452	0.426127597	0.088087622
**NMRK1**	0.303766425	0.235887056	0.442439827	0.075340694
**COLEC11**	0.566312892	0.017789246	0.586278847	0.013381437
**ELMOD1**	0.300090386	0.241889101	0.274034075	0.287168033
**CDH16**	0.071563087	0.784901743	0.781476136	0.000211701
**C11orf54**	0.227616873	0.379607705	0.826898319	4.25E-05
**RPF2**	-0.219486237	0.397314797	0.337348392	0.185438245
**SMIM8**	-0.233625811	0.36680603	0.205272984	0.42931359
**RUNDC3B**	0.186964764	0.47242906	0.609497768	0.009392022

**ZNF789**	-0.436367487	0.079917263	-0.435555549	0.080544181
**MMACHC**	0.034740935	0.894692483	0.274409394	0.286481799
**SLC5A11**	0.020166938	0.93876353	0.628478992	0.006891329
**PRODH2**	0.614244499	0.008707818	0.582307316	0.014180876
**CTSB**	0.398323238	0.113286783	0.766599498	0.000331031
**FAM21C**	-0.166629923	0.522696143	-0.538052202	0.025884914
**EGOT**	0.095522686	0.715345599	0.570239526	0.016843268
**NEBL-AS1**	-0.083529451	0.749934875	-0.400332818	0.111312183
**LGMN**	0.477034252	0.052841235	0.798956444	0.000119655
**IL2RB**	0.288226616	0.261910402	-0.158916912	0.542382452
**ANKRD31**	-0.588924124	0.012869028	-0.310142405	0.225702413
**PTGS1**	0.066811849	0.798897909	0.682959693	0.002514222
**DOPEY2**	0.184117565	0.479319976	0.333552436	0.190748116
**MSRA**	0.462701169	0.061451766	0.889571731	1.77E-06
**C15orf61**	-0.134367652	0.607143716	0.463294481	0.061075959
**METTL7A**	0.151945472	0.560455487	0.441786106	0.075823966
**KBTBD3**	0.079487628	0.761697642	0.255429483	0.32242504
**SOX17**	0.243514545	0.346272504	0.485157302	0.048380813
**ZNF302**	-0.384424644	0.127623193	-0.533890555	0.027284723
**CGN**	0.056544081	0.829337977	0.607310875	0.009721332
**RPS23**	0.04391893	0.867079641	-0.140740585	0.590037262
**IFI44L**	0.25282483	0.327554169	0.533586414	0.027389269
**MEF2C-AS1**	0.016902256	0.948663212	-0.504058769	0.039100878
**PCK2**	0.384260898	0.127799273	0.914611545	2.76E-07

**ZNF195**	-0.697849605	0.001840244	-0.534640765	0.027028163
**TMEM116**	-0.345878815	0.173865507	-0.365990881	0.148521785
**SERPINA6**	0.059883826	0.819409542	0.698834581	0.001801501
**ALDH4A1**	0.57884667	0.01490777	0.797733933	0.000124744
**GSTO2**	0.001036224	0.996850764	-0.49916369	0.041362136
**CKB**	-0.234839539	0.364249892	-0.175634676	0.500137959
**KLHL29**	-0.403406727	0.108339018	-0.534446295	0.027094489
**EPN2-AS1**	-0.37335978	0.139904926	-0.363336723	0.151712375
**KL**	0.419118119	0.094017271	0.734054029	0.000794359
**KHK**	0.340460253	0.181159029	0.88365349	2.57E-06
**C7orf13**	-0.153653436	0.556003634	-0.536163244	0.026513228
**EGFL6**	0.149921153	0.565751861	0.461559142	0.062179949
**EAF2**	0.275448234	0.284587563	0.805147498	9.65E-05
**CTSZ**	-0.244355793	0.344556588	-0.383720701	0.12838136
**CYP26B1**	-0.068080161	0.795155872	0.199504288	0.442672203
**MGC16275**	0.335502934	0.188007343	0.529462163	0.028837666
**FGFBP3**	0.198391692	0.445272884	0.492301835	0.044697001
**STK4-AS1**	0.028304134	0.914128478	0.097808806	0.708809049
**ALDH1L1**	0.210175418	0.418128265	0.883772748	2.55E-06
**LOC102723903**	0.304824185	0.234177649	0.048910728	0.852120178
**ABAT**	0.29556492	0.249408989	0.865369903	7.25E-06
**PRR15**	0.121931957	0.641078793	0.596042619	0.011566871
**FLVCR1-AS1**	0.002223447	0.993242675	0.383492602	0.128627699
**SUPT3H**	-0.40620304	0.105683612	-0.455054111	0.066450086

**COMMD9**	0.400054826	0.111583876	0.631436869	0.006555179
**ZNF112**	-0.600674878	0.010777252	-0.310427532	0.225253646
**SLC45A1**	0.192476752	0.459229027	-0.575285552	0.015686084
**ZFP69B**	0.031135424	0.905573068	-0.382603323	0.129591234
**MYADM**	0.100984304	0.699761429	-0.46813262	0.058074747
**HPN**	0.422220332	0.091358656	0.943386157	1.38E-08
**NR1I3**	0.218890867	0.398628662	0.589013484	0.012851994
**DCUN1D3**	-0.362264328	0.153014665	-0.337529719	0.185187076
**RTKN**	0.195357939	0.452403706	0.737550551	0.000727408
**LOC101927244**	-0.159638539	0.540526677	0.440065257	0.077106944
**LOC100507557**	-0.265242422	0.303526318	-0.252748859	0.327704479
**C21orf62**	-0.298793306	0.244029655	-0.268859959	0.296729493
**ATAD2B**	-0.578860563	0.014904794	-0.700255211	0.001746803
**PC**	0.329815138	0.1960729	0.504649283	0.038834576
**BANK1**	0.00156979	0.995229192	0.091098365	0.728048674
**PNPLA3**	0.029431312	0.910721327	0.771072511	0.000290389
**TANGO2**	-0.14097597	0.589409266	-0.434340676	0.081488881
**RBP5**	0.308241693	0.228708585	0.904690919	6.12E-07
**WFDC1**	-0.23321773	0.367667702	-0.301109126	0.240216211
**KLB**	-0.237084211	0.35954894	-0.107960916	0.680018708
**LOC401261**	-0.188623911	0.468436197	-0.521115133	0.031948936
**SNHG17**	-0.617773402	0.008225436	-0.525123767	0.030424186
**GDPD3**	0.556456108	0.020347735	0.441208547	0.076252814
**LOC102723493**	-0.0417912	0.873469319	0.517830449	0.033241428

**KIRREL2**	-0.015067995	0.954228554	-0.465405505	0.05975263
**IFI27**	0.541868801	0.024650596	0.43709181	0.079360996
**ZNF709**	-0.005497706	0.983292715	0.523702157	0.030958378
**DOHH**	-0.093075433	0.722363638	-0.051684677	0.843827423
**ZNF586**	-0.410572389	0.101627479	-0.44005115	0.077117526
**CEP57L1**	0.266380337	0.301378434	0.525032948	0.030458099
**IFIT3**	0.487189744	0.047310445	0.602212179	0.010524889
**CA10**	0.109051659	0.676949388	0.186410864	0.47376579
**CTSH**	0.08010601	0.759894718	0.622441354	0.007620432
**LAS1L**	-0.081175549	0.75677919	-0.506804807	0.037874192
**CLYBL**	-0.09884907	0.70584101	0.466941548	0.058803199
**LY6G5B**	-0.414064888	0.098466087	-0.377271749	0.135473323
**MX2**	0.272027943	0.290852874	0.423933701	0.089913698
**LONRF2**	-0.281552998	0.273609877	-0.649320028	0.004792484
**FHIT**	0.29026233	0.258404253	0.513827743	0.034870124
**SLC6A13**	0.245491889	0.342246994	0.842872806	2.16E-05
**STT3A**	-0.286354125	0.265161289	-0.462172454	0.061788097
**RARS**	-0.462646003	0.061486795	-0.477134046	0.052784637
**PITPNC1**	-0.152519252	0.55895819	0.566510867	0.017740577
**MX1**	0.332422835	0.192347371	0.463246886	0.061106043
**IRX4**	0.39754188	0.114061169	0.496019556	0.042865931
**A1CF**	0.26003752	0.313466591	0.745116363	0.000598427
**BTAF1**	0.025478268	0.922676395	-0.471999564	0.055755959
**ADIRF**	0.125697855	0.630727185	-0.339497087	0.182476427

**CCDC58**	-0.040474765	0.87742637	-0.01266505	0.961522329
**SMIM2-AS1**	-0.23844642	0.356712836	0.283864408	0.269522137
**GPX3**	0.554834967	0.020794664	0.823119247	4.94E-05
**ATP13A3**	0.439515893	0.077519836	0.530467647	0.028479235
**NR1H2**	-0.170399637	0.513195879	-0.247848103	0.337485358
**SLC23A1**	0.07176956	0.784294896	0.700459864	0.001739036
**NEURL1B**	0.061308062	0.815183345	-0.432592597	0.082862291
**FLJ20021**	-0.112571242	0.667078328	-0.207493295	0.42422859
**CLDN16**	0.048631524	0.852955689	0.609399032	0.009406695
**DDIT4L**	-0.132530447	0.612111748	-0.537376175	0.026108439
**DPEP1**	0.580535692	0.01454942	0.703939351	0.001611274
**ZNF600**	-0.585338301	0.013567462	-0.442486617	0.07530619
**KCNS3**	0.247884441	0.337412223	-0.360602352	0.155047936
**RGS2**	-0.584624406	0.013710018	-0.497102571	0.042343347
**ACIN1**	-0.096505611	0.712532867	-0.331275077	0.193981349
**APRT**	0.032422809	0.901686123	0.33879948	0.18343456
**SEC24B-AS1**	-0.366198766	0.148273837	-0.158323368	0.543910974
**RCC2**	0.258442986	0.316549746	0.284281631	0.268788298
**SLC28A2**	0.17194538	0.509323816	0.501633947	0.040208959
**DEPDC7**	0.426189263	0.088036685	0.737065248	0.000736411
**ENOX1**	0.146491955	0.574772687	0.463788402	0.060764409
**PCDHB7**	-0.324537541	0.203756869	-0.394183317	0.117432249
**LOC101928227**	-0.393016159	0.118619975	-0.476186088	0.053324099
**HAUS1**	-0.094050603	0.719564581	-0.351804681	0.16611699

**HIST1H4D**	0.310644618	0.224912353	0.373301406	0.1399718
**SPAG5**	0.412338153	0.100020207	0.502003054	0.040038767
**GBP2**	0.240385335	0.352697895	0.333614789	0.190660094
**CYP4Z1**	-0.053645082	0.837976057	-0.458471526	0.064180659
**EIF2AK4**	0.337430785	0.185324089	0.394921396	0.11668549
**RFTN2**	-0.364400285	0.150428295	-0.038286766	0.884009274
**CA11**	-0.319242197	0.211661285	-0.580658611	0.014523609
**KCNE3**	-0.124712457	0.633429655	0.398603658	0.113009773
**MTA2**	0.193548893	0.456683187	0.520594935	0.032151018
**TMEM37**	-0.006643542	0.979811181	0.693412537	0.002023344
**DPY19L2**	-0.068450401	0.794064305	0.569798423	0.016947522
**DCXR**	-0.063921583	0.807440726	0.35100148	0.167153362
**TLN2**	0.309452946	0.226789928	0.769878226	0.000300808
**HSPC324**	0.55026709	0.022095209	0.592702481	0.01216418
**TYMS**	0.242055617	0.349259848	0.315913833	0.216729734
**XAB2**	0.146455164	0.5748698	0.452810575	0.067971782
**DRAP1**	0.097732908	0.709025755	-0.276180274	0.283257321
**SLC5A9**	0.210394728	0.417631516	0.551341323	0.021783819
**SNORA25**	-0.080340764	0.759210587	-0.625635633	0.007227373
**HNF4G**	0.20699539	0.42536615	0.668390342	0.003356695
**PQLC3**	-0.244954264	0.343338835	0.32372871	0.204951584
**SYT13**	-0.253734541	0.325757388	0.581948229	0.014254974
**ATG4A**	0.091327405	0.727389381	0.117937248	0.652128076
**HSPA12B**	0.528323704	0.029247681	0.651403846	0.004614897

**ATP23**	0.097544743	0.709563097	0.343221037	0.177417973
**ACY3**	0.015271071	0.953612296	0.473814757	0.05469161
**LOC284578**	0.146237368	0.575444825	0.669723704	0.003271155
**CAT**	0.322588037	0.206644193	0.416372901	0.096415855
**TRHDE**	0.323328369	0.205544606	0.634296954	0.006242798
**ALPK1**	0.631813531	0.006513336	0.289518166	0.259682544
**LOC100288152**	-0.386316046	0.125601554	-0.480031011	0.051161316
**LOC102723665**	0.100440362	0.701308557	0.598853375	0.011082467
**HOXC10**	-0.011207662	0.965947452	0.16343371	0.530813917
**C1orf115**	0.330119698	0.195635363	-0.352579068	0.165121902
**KIAA0753**	-0.14039123	0.590969838	-0.265880718	0.302320376
**TFEC**	0.184743257	0.477801452	0.753746628	0.000475074
**LOC100505555**	-0.056433181	0.82966809	-0.286385926	0.265105872
**PDDC1**	-0.2916019	0.256113062	-0.299613736	0.24267433
**DGCR8**	-0.186795448	0.472837473	-0.298227745	0.244966712
**WIPF3**	-0.436174177	0.0800662	-0.62642451	0.00713285
**LRRC59**	-0.257979803	0.317448675	-0.440405621	0.076851939
**RAB11FIP3**	0.628789495	0.006855405	0.757451411	0.000429043
**CTSC**	0.285096146	0.267359221	0.462521677	0.061565793
**MAP2**	0.350489582	0.16781613	0.31697073	0.215111891
**MACROD2**	0.339048998	0.183091476	-0.369761178	0.144068815
**NAPB**	-0.349979532	0.16847826	-0.728699877	0.000906713
**PCED1B**	-0.303632241	0.23610447	-0.568749226	0.017197535
**RAP1GAP**	0.027756048	0.915785708	0.543443214	0.024154914

**SACS**	-0.338388339	0.184000806	-0.569725724	0.016964753
**INTU**	-0.158509825	0.543430595	-0.318405498	0.212928128
**AMDHD1**	0.225975229	0.38314738	0.583127798	0.014012709
**NEK4**	-0.62371092	0.007462211	-0.550008883	0.022170569
**MYH8**	-0.300906374	0.240548569	0.377037004	0.135736477
**LARGE1**	-0.340262267	0.181429312	-0.698513619	0.001814051
**REEP6**	0.222008826	0.391773967	0.739772818	0.00068734
**ZNF274**	-0.33479013	0.189005915	-0.714316273	0.001275199
**SDHAF4**	-0.421539605	0.091937355	-0.630955014	0.006609023
**F11**	0.463657862	0.060846634	0.569674495	0.016976904
**SPTLC3**	0.293525612	0.25284494	0.422252819	0.091331105
**GBP1**	0.385065445	0.126935748	0.731474433	0.000846956
**NXT1**	-0.287090469	0.263879945	-0.428110463	0.08646036
**SLC51A**	0.289240547	0.260160428	0.525143363	0.030416872
**EHD4**	0.139816582	0.592505136	-0.274654694	0.28603383
**PRSS8**	0.243128222	0.347062126	0.555913766	0.02049641
**AGA**	0.081802941	0.75495326	0.497772395	0.042022561
**SIGIRR**	0.392438053	0.119211379	0.346751828	0.17270907
**PIGL**	-0.483321862	0.049362976	-0.578375471	0.015008971
**EIF2AK3**	-0.024674189	0.925110144	-0.44625732	0.072563398
**SLC30A1**	-0.105343722	0.687402735	-0.182287259	0.48377555
**ABHD18**	-0.272873875	0.289295619	-0.445207984	0.073319212
**GRAMD1C**	0.243609253	0.346079081	0.799474651	0.000117551
**PCDHB6**	-0.122836836	0.638585678	-0.646512943	0.005040401

**FLVCR1**	-0.279950063	0.276466832	-0.596737406	0.011445599
**REV3L**	-0.073809482	0.778305694	-0.085992709	0.742791439
**ELMOD3**	-0.025981745	0.921152824	-0.387331786	0.124525143
**PRLR**	0.129212985	0.621123072	0.678939913	0.002727136
**TMEM220**	-0.523537527	0.031020703	-0.207452023	0.424322824
**WDR53**	0.002533416	0.992300665	-0.46119065	0.062416265
**GPR158**	0.005347166	0.983750141	-0.256275573	0.320769063
**FZD5**	0.13630219	0.601930005	0.419590835	0.093608608
**CLCN4**	0.017059115	0.948187389	0.266880979	0.300436321
**ZNF212**	-0.22280146	0.390041677	-0.454130124	0.067073715
**LTK**	0.157513955	0.545998498	0.273504919	0.288137227
**RNASE1**	0.396882133	0.114717926	0.547054813	0.023047049
**MAPK8IP1**	-0.00138393	0.995794039	-0.299728632	0.242484906
**ENO2**	0.468920601	0.057596523	0.627355377	0.007022592
**PCDHB4**	0.064390729	0.806052636	-0.405507508	0.106339733
**C6orf1**	0.0823803	0.753274031	-0.529875317	0.028689968
**PHGDH**	0.194782879	0.453761863	0.462976929	0.061276885
**ZNF16**	-0.71119077	0.00136969	-0.446413871	0.072451128
**INVS**	0.310342014	0.225388184	0.399232377	0.112390434
**CCL13**	0.466683532	0.058961891	0.494073936	0.04381698
**GPD1**	0.569304097	0.017064957	0.689973986	0.002175278
**FANK1**	0.132772888	0.611455235	-0.247663522	0.337856992
**LOC100506730**	0.074915583	0.775063088	0.235020729	0.363869161
**ZNF528**	-0.221524473	0.392834569	-0.014810507	0.955009966

**KYAT1**	-0.320007504	0.210506818	-0.527537286	0.029533519
**GPER1**	0.227827042	0.37915585	0.535176405	0.026846126
**LOC100996273**	0.015853936	0.951843668	-0.31220282	0.222472325
**SULF2**	-0.135864046	0.60310925	-0.467977368	0.058169316
**LOC102723704**	-0.222574867	0.390536466	-0.560564884	0.019248517
**TTLL5**	-0.294123585	0.251834395	-0.341862884	0.179251871
**C1orf50**	-0.283255483	0.270595362	-0.5232395	0.031133772
**PPP1R16B**	0.178621063	0.492760423	0.618029567	0.008191269
**LOC100506473**	0.159484067	0.540923685	0.417758912	0.095199439
**ZSWIM6**	-0.062828432	0.810677198	-0.468678397	0.057743203
**SIGLEC16**	0.213618146	0.410366316	0.447526775	0.071656684
**DBR1**	-0.30383944	0.235768807	-0.422698563	0.090953674
**TPK1**	0.394761443	0.11684704	0.362020489	0.153311837
**CATSPER1**	0.011487138	0.96509879	0.389977054	0.121752139
**ADAMTS9-AS1**	0.215874897	0.405320204	-0.188181489	0.46949928
**C1RL-AS1**	0.098956321	0.705535231	0.445127268	0.073377588
**COQ3**	0.489086856	0.046327516	0.152148714	0.559924922
**NUP205**	-0.217171872	0.402435267	0.235821266	0.362189673
**DCP1B**	0.220180726	0.395785157	-0.37665102	0.136169939
**SPRR2A**	-0.104200707	0.690636032	-0.151737409	0.560998863
**MSH3**	0.079746312	0.760943295	0.479063236	0.051699393
**LOC101927151**	-0.439640158	0.0774263	-0.499982753	0.040977036
**GPD1L**	-0.381205514	0.131115883	-0.755733383	0.000449904
**PSMD11**	0.136480375	0.601450696	-0.151263457	0.562237487

**ENAM**	0.288559942	0.261334302	0.598465204	0.011148389
**DNTTIP1**	-0.313152243	0.220993954	-0.323176526	0.205769821
**CAND2**	-0.425444774	0.088653071	-0.422917644	0.090768581
**MYLK4**	0.32688258	0.20031874	0.474067014	0.054544906
**ZNF426**	-0.226114681	0.382845996	-0.654525243	0.004358877
**TNNC2**	-0.375498393	0.137470039	-0.314171922	0.219413226
**LOC646762**	-0.240975619	0.351480691	-0.515359723	0.034239721
**TCFL5**	0.279355581	0.277531006	0.483795928	0.049107878
**APCDD1**	-0.007676564	0.976672714	-0.431643167	0.083615224
**SLC20A1**	-0.055226971	0.833260307	-0.418815718	0.094279368
**POLR2H**	-0.60027199	0.010844178	-0.252539401	0.328119106
**RASA2**	-0.25704566	0.319266178	-0.291051603	0.257052752
**USP35**	-0.054328388	0.835938415	-0.245246274	0.342745556
**PDCD4-AS1**	-0.068480183	0.793976515	0.656699051	0.004187469
**CRYL1**	0.230864971	0.372657581	0.741209853	0.000662418
**POLR1A**	-0.226749879	0.381474847	-0.432885555	0.08263096
**MLH3**	0.013696876	0.958389993	-0.457443236	0.064857408
**PIP5K1B**	-0.410013122	0.102140363	-0.363315413	0.15173818
**ATP6V0E2**	0.540927708	0.024950626	0.521598168	0.031762164
**ESRP1**	-0.475982141	0.053440698	0.482407165	0.049857991
**MED29**	-0.293292093	0.253240263	-0.544987119	0.023676377
**SH3D21**	-0.27358253	0.287994954	-0.329609873	0.196368151
**NNT-AS1**	-0.259303441	0.314883783	-0.515199319	0.034305315
**LRIF1**	-0.215845021	0.40538679	-0.518069458	0.033146052

**SNRPB**	0.200788082	0.43968105	-0.281036081	0.27452921
**PCCA**	0.259818352	0.313889317	0.621768047	0.007705427
**PCDH20**	0.101997557	0.696882423	0.361791844	0.153590848
**TMEM161B**	-0.239812172	0.353882077	-0.374379884	0.138739824
**RBM26-AS1**	-0.208460841	0.422022613	-0.539152907	0.025524139
**NPY6R**	0.358914395	0.157131713	0.676738104	0.002849852
**PTPDC1**	-0.091538162	0.726782871	-0.644073792	0.005264139
**PCDHAC1**	-0.35077386	0.167447849	-0.573060379	0.016188405
**SQLE**	0.029932945	0.909205494	-0.166396306	0.523287542
**DUBR**	-0.140943365	0.589496237	-0.445162324	0.07335223
**MCOLN1**	0.532081159	0.027911241	0.331303002	0.193941486
**SEC24D**	0.042829966	0.870348905	0.388219657	0.123589529
**SLC37A4**	-0.020187713	0.938700559	0.608233377	0.009581315
**SYT11**	0.616096204	0.008451961	0.428898219	0.085819949
**SLC2A5**	0.069881209	0.789849313	0.692536614	0.002061195
**TMEM45A**	0.581869888	0.01427118	0.360344122	0.155365495
**FOLR1**	0.235201584	0.363489354	0.446803564	0.072172216
**TTF2**	-0.127900911	0.62470129	0.028122343	0.914678117
**ANXA13**	0.776204415	0.000248978	0.710875779	0.001379525
**RTN4IP1**	-0.073505439	0.779197622	-0.14132881	0.588468417
**C15orf57**	-0.356690886	0.159905519	-0.690550974	0.002149152
**SRFBP1**	-0.158301513	0.543967293	-0.335601637	0.187869345
**ATP6V1B2**	0.456067363	0.065771134	0.614064064	0.008733076
**FOXM1**	-0.014264033	0.956668503	0.564190902	0.018317503

**TRANK1**	0.011364263	0.965471912	-0.19222184	0.45983537
**ZNF311**	0.059453585	0.820687145	-0.369218804	0.144703682
**CDHR5**	0.278294127	0.279437304	0.807070858	9.01E-05
**TMEM178B**	0.166683004	0.52256181	0.244225968	0.344821077
**SERINC5**	-0.341645049	0.179547181	-0.486697053	0.047568265
**TNFSF10**	0.263084768	0.307623958	0.442231651	0.075494346
**SLC39A5**	0.194116522	0.455338214	0.719988388	0.001117433
**CKS2**	0.223364573	0.388813538	0.27818259	0.279638078
**LMBRD2**	0.126052576	0.629755437	0.553373196	0.021204182
**PAXIP1-AS1**	0.133708335	0.608924735	-0.241959594	0.349456979
**ELOVL6**	-0.249476082	0.334217803	-0.322517239	0.206749546
**IGHD**	-0.081313548	0.75637746	0.279711149	0.276894209
**NMT2**	-0.267221405	0.299796709	-0.444303606	0.073975226
**NAP1L3**	-0.114501953	0.661685164	-0.522432523	0.031441523
**DNAJC22**	0.257790956	0.317815611	0.796392106	0.000130538
**FBXL2**	-0.030875449	0.906358247	-0.555347579	0.020652524
**LRP4**	0.338659768	0.183626849	0.643746328	0.005294777
**PRSS54**	-0.298934798	0.243795577	-0.631418423	0.006557234
**HDAC4**	-0.205038209	0.429853123	-0.480711887	0.050785275
**HS6ST2**	-0.040990646	0.875875361	0.593113263	0.012089431
**MIR17HG**	-0.423002491	0.09069697	-0.698696508	0.001806891
**ZNF606**	-0.406474606	0.105428216	0.063186573	0.809616534
**BMPER**	0.284188654	0.268951725	0.640047993	0.005650896
**MFSD4A**	0.24899195	0.335187602	0.744983439	0.000600517

**SLC22A4**	-0.180248407	0.488762379	0.598601726	0.011125168
**NAIF1**	0.059118118	0.821683614	0.293422893	0.253018785
**ZMAT3**	0.138150159	0.596966579	-0.530677941	0.028404706
**SLC16A4**	0.587539706	0.013135227	0.722100081	0.001062978
**KIF18B**	0.302429871	0.238058289	0.368225183	0.14587172
**PGAP3**	0.11167671	0.66958233	0.414548846	0.098033623
**HSPBAP1**	-0.598467082	0.011148069	-0.568944567	0.017150769
**NNT**	-0.139556182	0.59320139	0.485086633	0.048418355
**TBC1D17**	0.349770451	0.168750189	-0.047481566	0.856398456
**PRKCSH**	0.033401026	0.89873404	-0.409447271	0.10266116
**MCCC2**	0.119945911	0.646563427	0.462952149	0.061292585
**RIN1**	-0.137896641	0.597646513	-0.499781287	0.041071506
**GTF3C5**	0.367888907	0.146268484	0.413822751	0.098682972
**SOWAHC**	-0.250463541	0.332244788	-0.601352218	0.010665474
**SLC22A7**	0.316425726	0.215945179	0.78794596	0.000172445
**SFT2D2**	0.181801957	0.484960303	0.500208911	0.040871183
**IFRD1**	-0.227799857	0.379214281	-0.694660532	0.001970399
**AMN**	0.065892376	0.801613322	0.656778604	0.004181302
**FAM99B**	0.449632134	0.070171277	0.522465677	0.031428834
**FLNA**	0.283579681	0.270023641	-0.392773657	0.118867804
**LOC389332**	0.409838561	0.102300824	0.777578508	0.000238771
**ASTE1**	0.120424823	0.645239283	-0.429146468	0.085618845
**OLAH**	-0.097111023	0.710802162	-0.844055416	2.05E-05
**ZNF541**	0.430600045	0.084448161	0.391400813	0.12027766

**LNX1**	0.166947877	0.521891737	0.229279372	0.376041498
**RPARP-AS1**	-0.070713078	0.787401248	0.453528612	0.067482005
**LOC100128288**	-0.239699417	0.354115297	-0.39538782	0.116215307
**PROSER3**	0.078719827	0.763937817	-0.25422811	0.324784941
**HMG20B**	-0.105390631	0.687270153	0.321967244	0.207569175
**LCAT**	-0.457940423	0.064529539	-0.586258015	0.013385536
**MELTF**	-0.097946635	0.708415571	-0.519657178	0.032517783
**ANKRD19P**	0.146007592	0.576051743	0.280669308	0.275182657
**LBX2-AS1**	0.154129384	0.554765824	0.606634802	0.009824977
**LINC00685**	-0.06065458	0.817121859	-0.486965367	0.047427728
**SNHG9**	0.01636228	0.950301334	-0.426969646	0.087393908
**KIAA1024**	-0.327501173	0.199418165	-0.428758369	0.085933389
**GGH**	0.351896923	0.165998247	0.891925785	1.51E-06
**PRKCD**	-0.08046294	0.758854603	0.031381858	0.904828863
**PCCB**	0.045312853	0.862897811	0.420855058	0.092521954
**GATB**	0.354330844	0.162885712	0.561738648	0.018943185
**MACROD1**	0.627365187	0.007021437	0.366538982	0.147868668
**GIPC2**	0.236316237	0.361153427	0.86792152	6.33E-06
**ZMIZ2**	0.115997486	0.657518372	-0.394455904	0.117156066
**RFC2**	-0.249073801	0.335023525	-0.344999938	0.17503493
**ADAMDEC1**	-0.12923947	0.621050925	0.279268482	0.277687131
**LOC101926963**	0.198068236	0.446030421	0.59834703	0.011168519
**KLHDC9**	-0.145696949	0.576872689	-0.541993479	0.024611058
**ALLC**	0.209131291	0.420497548	0.30194465	0.238849646

**ZNF26**	-0.581122061	0.014426614	-0.604140264	0.01021504
**GABPB1**	-0.045878587	0.86120156	-0.248094723	0.336989184
**ALPL**	0.399587245	0.11204192	0.791635198	0.000152933
**DDHD2**	-0.410593742	0.101607934	-0.473925872	0.054626953
**CDKN2AIP**	-0.406984939	0.104949456	-0.409247734	0.102845259
**DDAH1**	0.298691863	0.244197567	0.826182151	4.37E-05
**HSPA1L**	-0.193729866	0.456254162	0.33774236	0.184892821
**SLC15A1**	-0.229173345	0.376268382	0.159348926	0.541271119
**CTU2**	0.147856407	0.571176073	-0.16460076	0.527843203
**DCP1A**	-0.195088017	0.453040942	-0.457091059	0.065090393
**TCAM1P**	-0.27850618	0.279055837	0.217207139	0.402356974
**NTHL1**	0.035053723	0.893749371	0.374000247	0.139172638
**CRYBB2**	-0.553584947	0.021144474	-0.568835584	0.017176848
**ZKSCAN7**	0.164543564	0.527988616	0.35936724	0.156570823
**GAL3ST4**	0.200347352	0.440706746	0.28515924	0.267248716
**PHTF1**	-0.321216215	0.2086918	-0.419379444	0.093791198
**ACOT4**	0.325153808	0.202849642	0.625470663	0.007247266
**NR2F1-AS1**	0.19424854	0.455025691	-0.435976748	0.080218518
**GPR137B**	0.036885278	0.888229647	0.383531991	0.128585138
**MAST3**	-0.007306135	0.977798094	0.379621934	0.132858152
**TMEM139**	0.20125669	0.438591819	0.377398221	0.135331692
**CA12**	0.0847318	0.746445628	0.639852603	0.005670234
**VDR**	0.193074761	0.457808155	0.824701547	4.64E-05
**GEMIN5**	0.061614836	0.814273667	0.304462217	0.234761725

**PEPD**	0.542234887	0.024534642	0.788680381	0.000168403
**ZNF76**	-0.038662711	0.882877668	-0.329893307	0.19596054
**TAGLN2**	0.176368932	0.49831921	-0.381902755	0.130353825
**HIST1H1E**	-0.155406923	0.551449278	-0.190434475	0.464098119
**NAGK**	0.443633471	0.074464089	0.486167067	0.047846779
**LLPH**	-0.182580812	0.483059592	-0.258567829	0.316307711
**NOP58**	-0.478743071	0.051878332	-0.507752051	0.037457914
**LOC102723335**	-0.287749978	0.262735556	-0.372165747	0.141277219
**GMPPB**	0.287556344	0.263071234	0.388111486	0.123703253
**SNRNP25**	-0.004390469	0.986657242	-0.451360432	0.068968902
**DHTKD1**	0.42224124	0.091340924	0.553042421	0.021297715
**CLDND2**	-0.418573741	0.094489471	-0.475583758	0.053669006
**ZNF69**	0.026584979	0.91932772	0.558109611	0.019899639
**LOC148709**	-0.010394044	0.968418286	0.462044593	0.061869637
**SCN7A**	-0.097865749	0.708646479	-0.113985106	0.663127374
**ZNF19**	-0.617425244	0.008272055	-0.275085142	0.285248766
**SYTL1**	0.051646936	0.843940149	-0.261495397	0.310663228
**SLC25A11**	0.054921779	0.834169698	0.514578867	0.034559942
**HIBCH**	0.416459907	0.096339169	0.69682085	0.001881435
**VASH2**	-0.213134161	0.411452844	-0.229399149	0.375785285
**DNAJC15**	0.121717878	0.641669151	0.181248871	0.486312249
**POLE3**	0.597029361	0.011394941	0.387572315	0.124271193
**ARHGEF10**	0.091708975	0.726291428	-0.14901724	0.568123767
**RPA3**	-0.451163048	0.069105449	-0.240472237	0.352518547

**NUAK2**	0.158898659	0.542429431	0.293698248	0.252552935
**MT1F**	0.335637695	0.187818948	0.575549121	0.015627406
**PLBD2**	-0.095483403	0.715458084	-0.421813443	0.091704244
**CRYZ**	0.33008956	0.195678631	0.682419622	0.002542011
**NOP9**	-0.017321277	0.947392167	-0.342184098	0.178817005
**PROZ**	0.180867465	0.487245607	0.578762426	0.014925824
**SCLT1**	0.069423368	0.791197468	0.380373808	0.132028944
**MUC15**	-0.169511703	0.51542633	0.421489157	0.091980346
**IDH2**	0.533344061	0.027472796	0.761172082	0.000386613
**VWA7**	0.193476754	0.456854261	-0.206663991	0.426124177
**GTSE1-AS1**	-0.180151007	0.489001228	-0.393938809	0.117680371
**MDH1**	0.217127606	0.402533549	0.302290022	0.238286201
**BCHE**	0.601984916	0.010561897	0.382011107	0.130235676
**DYRK2**	-0.247040534	0.339113047	-0.489771537	0.045976576
**ZNF713**	-0.3788397	0.133724666	-0.551928523	0.021615055
**TEF**	-0.178192392	0.493816182	-0.398745393	0.112869943
**RAB11FIP5**	0.494293419	0.043708903	0.585677072	0.013500225
**FANCL**	-0.317966193	0.213595235	-0.52013432	0.032330771
**PVALB**	0.547939594	0.022781758	0.690601486	0.002146877
**MRC2**	0.12572543	0.630651625	-0.16857773	0.517777284
**HRH1**	-0.379867033	0.132587448	-0.590510894	0.012569188
**SFXN2**	0.092481518	0.724070007	0.497715925	0.042049533
**ICA1L**	-0.497926958	0.041948801	-0.529432204	0.028848399
**UBXN7-AS1**	0.037217621	0.887228584	-0.495832652	0.042956609

**USP46-AS1**	-0.235473843	0.362918014	-0.490406788	0.045652765
**ACO1**	0.188501456	0.468730322	0.675563657	0.002917127
**SLC25A5-AS1**	0.299332293	0.243138731	0.700869376	0.001723581
**AK1**	0.238316576	0.356982625	-0.35423497	0.163007565
**PPP3CC**	-0.450183263	0.069786198	-0.546970516	0.023072449
**WDPCP**	-0.467570918	0.05841744	-0.506965721	0.037803229
**RIOK1**	0.423108239	0.090607776	-0.196406722	0.449932036
**AKAP12**	-0.179761739	0.489956376	-0.22694615	0.381051721
**PLIN2**	0.234582312	0.364790783	0.461625076	0.062137735
**C8orf48**	-0.341252724	0.180079856	-0.529650354	0.028770318
**TREH**	0.472596023	0.055404534	0.602089421	0.010544866
**C1orf54**	0.289757587	0.25927085	0.658762187	0.00402988
**LAMP3**	-0.03074314	0.906757881	0.314558882	0.21881526
**TTYH3**	0.333259005	0.191162701	0.389391212	0.122362509
**TSKU**	0.47927741	0.05157995	0.37839391	0.134220234
**FAM209A**	0.043320046	0.86887735	-0.444297671	0.073979546
**ACOT9**	-0.301784198	0.239111696	-0.370043384	0.143739242
**KEL**	0.069269829	0.791649701	-0.275157861	0.285116268
**FAM90A1**	-0.028822772	0.912560596	-0.572518919	0.016312527
**ITGA3**	-0.148823073	0.568633825	-0.49817252	0.041831816
**TOPBP1**	-0.268535979	0.297334456	-0.30458374	0.234565532
**SDR42E1**	-0.281010329	0.274575059	-0.402822386	0.108899829
**ARL13B**	-0.006032265	0.981668459	-0.446687741	0.072255031
**TXNDC9**	-0.377751325	0.134936807	-0.507722291	0.037470939

**IFITM2**	-0.081786149	0.755002116	-0.338437251	0.183933381
**PPP4R1**	-0.158108529	0.544464708	-0.331683875	0.19339833
**HOGA1**	0.256170141	0.320975145	0.70575191	0.001547838
**GPT2**	0.494505352	0.043604735	0.801243217	0.000110603
**DLST**	-0.276543425	0.282598816	-0.186955662	0.472451011
**PHYHIPL**	0.129714831	0.619756578	0.490038782	0.045840141
**ACTRT3**	0.427110406	0.08727833	0.481356828	0.050430995
**ETV7**	0.390570541	0.12113598	0.564373287	0.018271623
**LINC00662**	-0.117211417	0.654143158	-0.498653828	0.04160324
**ERBB4**	-0.262376966	0.308975266	0.08978516	0.731832224
**TMEM101**	0.456620175	0.065402874	0.368380832	0.145688324
**FKBP1A**	0.249660617	0.333848576	0.53640258	0.026432973
**CASC1**	-0.464725471	0.060176574	-0.386073572	0.125859468
**SLC28A1**	0.313215889	0.220895075	0.580727906	0.014509073
**ERVK13-1**	-0.567563248	0.017483613	-0.300248936	0.241628262
**BCS1L**	-0.047400784	0.856640396	-0.345376078	0.174533802
**ACBD6**	-0.208349753	0.422275584	-0.432884934	0.08263145
**SUSD3**	0.167481931	0.520541896	0.598766654	0.011097168
**NOS3**	0.183754994	0.480200997	-0.073329828	0.779712906
**CMTM7**	0.017886618	0.945677467	-0.149484136	0.566898081
**CACHD1**	-0.204272095	0.431616173	0.277939881	0.280075275
**RAB17**	0.384501701	0.12754039	0.701289849	0.00170783
**FUK**	0.092901322	0.722863747	-0.306852018	0.230922576
**FZD2**	-0.259459778	0.314581648	-0.467081314	0.058717369

**C1orf131**	0.072662534	0.781671697	0.307185138	0.23039062
**LMBR1L**	-0.261684436	0.310300811	-0.472197743	0.055639011
**LOC101928047**	0.331638179	0.193463444	0.573224619	0.016150902
**CDH17**	-0.110785265	0.672080967	0.195728023	0.451530743
**ACACA**	-0.038732259	0.882668349	-0.419774408	0.093450252
**METTL21A**	-0.371186647	0.142409381	-0.546122106	0.023329301
**NCEH1**	0.354024245	0.163275609	0.470245944	0.056798792
**WARS**	0.417188183	0.095698992	0.7040218	0.001608342
**MRE11A**	0.031316511	0.905026197	-0.59770537	0.011278329
**TAF4B**	-0.247320828	0.338547592	-0.559279968	0.019587157
**GNPDA1**	0.287858729	0.262547145	0.579313804	0.014807973
**VWCE**	-0.185009402	0.477156244	-0.456590993	0.065422276
**KLHDC1**	-0.217101914	0.402590596	-0.353087156	0.164471196
**SYBU**	-0.154663084	0.553379253	-0.523761739	0.030935845
**ASNS**	-0.620243204	0.007900724	-0.567750043	0.017438309
**CYP51A1**	-0.294997726	0.250361682	-0.140414334	0.590908145
**ACAT1**	0.269328601	0.295855711	0.789694789	0.000162951
**SMPDL3A**	0.435749542	0.08039407	0.565218466	0.018060186
**FOXJ2**	-0.414394685	0.098171233	-0.455413926	0.066208395
**SORCS2**	-0.259603253	0.31430452	-0.584331248	0.013768899
**NAF1**	-0.17794118	0.494435389	-0.357349943	0.15907992
**OSMR**	-0.4052312	0.106601185	-0.640683895	0.005588328
**POLR2J**	-0.103403674	0.692893632	-0.343147067	0.177517531
**RHOC**	0.619021084	0.008060092	0.43511082	0.080889079

**MRPL24**	-0.326527946	0.200836228	-0.160787804	0.537577047
**CHN1**	-0.242454884	0.348440844	-0.436890104	0.079515619
**CALML3**	0.320920295	0.209135215	0.308019494	0.229061674
**ICA1**	0.376573386	0.136257238	0.45085616	0.069318146
**CMC4**	-0.490074877	0.045821737	-0.684861633	0.002418326
**TRIM39**	-0.202080097	0.436681266	-0.348681925	0.170170679
**RABAC1**	0.11218685	0.668153924	-0.46942791	0.057290193
**INTS1**	-0.225438944	0.384307616	-0.480066308	0.051141771
**PMEL**	0.07766174	0.767027843	0.000587527	0.998214415
**FLRT1**	0.253698766	0.32582794	0.394200038	0.117415294
**WDR12**	-0.25091232	0.331350328	0.309101357	0.227345791
**SMIM10**	-0.023213887	0.929531712	-0.019292874	0.941413233
**LOC101929648**	0.08158771	0.755579521	0.134377155	0.607118059
**C5orf56**	0.061395469	0.814924134	-0.500388555	0.040787249
**NRTN**	0.009973538	0.969695407	0.254983794	0.323299351
**SULT1C4**	-0.41275629	0.099642274	-0.169291205	0.515980908
**DNAAF2**	0.361780448	0.153604762	-0.314487802	0.21892502
**KCNN3**	0.486674539	0.047580071	0.473982628	0.05459395
**NOL8**	-0.307008511	0.230672576	-0.642588054	0.005404304
**LOC101929004**	-0.144305767	0.580555257	-0.340656148	0.180891863
**MAK**	-0.539407016	0.025441406	-0.444967335	0.073493356
**SCPEP1**	0.07902754	0.763039802	0.445651912	0.07299876
**RAB8A**	0.234361188	0.365256116	0.267636525	0.299017861
**NUDT14**	0.308808913	0.22780881	0.394746939	0.116861697

**TADA3**	-0.023443865	0.928835241	-0.513487415	0.035011363
**PPIL6**	-0.317257269	0.21467462	-0.252050058	0.329088962
**HSD17B11**	0.316095576	0.216450975	0.3387159	0.183549578
**CCND1**	0.563911446	0.018387977	0.247512427	0.338161381
**GALNT11**	0.539386496	0.02544808	0.792663869	0.000147836
**HIPK1-AS1**	-0.529946463	0.028664593	-0.471871755	0.055831478
**IER5L**	-0.267754561	0.298796622	-0.570446426	0.016794541
**TP53I13**	-0.049193357	0.85127457	-0.293775416	0.252422476
**LINC00955**	-0.046164138	0.860345605	-0.322322934	0.207038868
**RPA2**	0.325635647	0.20214215	0.356864816	0.159687357
**SCD**	0.044162078	0.866349944	0.028069545	0.914837755
**TRAPPC12**	0.047549006	0.856196486	0.14313131	0.583671789
**TIMM17B**	-0.280062752	0.27626539	-0.415754863	0.09696184
**RPL11**	-0.423967101	0.089885694	-0.500715513	0.040634821
**CISD1**	0.123046315	0.638009044	0.261785887	0.310106419
**PPFIBP2**	-0.222616091	0.390446423	-0.390515847	0.121192671
**SDHAF3**	0.028205415	0.914426946	0.393938296	0.117680892
**GEMIN4**	-0.419062607	0.094065345	-0.317482134	0.214331867
**C16orf70**	-0.111574918	0.669867478	-0.462825283	0.06137301
**CCDC125**	0.292609359	0.254398268	-0.031422557	0.904705961
**PARG**	0.346668133	0.172819713	0.41910602	0.094027748
**GLOD5**	0.406353003	0.105542524	0.627890853	0.006959789
**MICU1**	0.326080595	0.201490256	0.741734594	0.000653506
**PCDHB15**	-0.481864368	0.050153497	-0.481154158	0.050542126

**RHPN2**	0.018980282	0.942360987	0.667204767	0.003434272
**LAP3**	0.278539793	0.278995397	0.683531437	0.002485074
**POLR1E**	-0.095432957	0.715602541	-0.118427077	0.650769477
**NPIPB15**	0.410575678	0.101624468	0.378269713	0.134358526
**MYOM3**	0.198382393	0.445294654	0.578945686	0.014886572
**MAP4K2**	0.417941792	0.095039762	0.408318829	0.10370539
**TLCD2**	-0.334813187	0.188973561	-0.531703823	0.028043283
**DISP2**	-0.19207656	0.460181119	-0.344862572	0.175218182
**TAP1**	0.438861163	0.078014021	0.468014228	0.058146853
**PTPRK**	-0.54183151	0.024662431	-0.616457061	0.008402807
**TOB1-AS1**	0.081041651	0.757169037	-0.088411134	0.73579727
**DANCR**	0.046907253	0.858118766	-0.390406892	0.121305662
**NFXL1**	-0.301457279	0.23964618	-0.335993289	0.187322428
**LINC00475**	0.388079336	0.123737068	0.279430606	0.277396567
**C8orf76**	-0.092999685	0.7225812	-0.159129661	0.54183504
**HIST1H3C**	-0.028829045	0.912541634	-0.226474094	0.382069829
**PEMT**	-0.107533336	0.681223205	-0.534989963	0.026909381
**DEFA4**	0.199301007	0.443146789	0.337038764	0.185867652
**C14orf79**	-0.174774032	0.50227378	0.149748065	0.566205723
**IGLV3-25**	-0.369693322	0.144148139	-0.374907659	0.138139665
**KNG1**	0.000732374	0.997774205	0.683596212	0.002481789
**ZNF222**	-0.256745464	0.319851541	-0.500529688	0.0407214
**MYD88**	0.529854105	0.028697537	0.438573135	0.078232149
**MYH16**	0.086237448	0.742082771	0.141399686	0.588279503

**SLC5A2**	0.266511301	0.301131814	0.469893264	0.057010264
**LOC100505622**	0.048116208	0.854498146	0.485077419	0.048423251
**ARID5A**	0.080264351	0.759433257	-0.380340754	0.132065322
**SLC19A3**	0.521008811	0.03199016	0.68596391	0.002364131
**SMCR5**	-0.127542795	0.625679309	-0.40611626	0.105765317
**COL26A1**	0.318064189	0.213446305	0.27202748	0.290853729
**CCM2L**	0.48662446	0.047606341	0.406458953	0.105442925
**MCU**	0.027487139	0.91659892	-0.369214982	0.144708163
**IFI44**	0.106716686	0.683525738	0.30126146	0.239966689
**SPRYD4**	-0.181591858	0.485473647	0.380800749	0.131559691
**DPAGT1**	0.547370646	0.022952076	0.456474731	0.065499615
**TMEM231**	-0.195732112	0.451521103	-0.370558235	0.143139308
**NT5DC2**	0.175261868	0.501062609	-0.357248597	0.159206688
**SNX30**	-0.080174582	0.759694865	0.513637279	0.034949114
**TMEM106A**	-0.086532736	0.741227992	0.460715713	0.062721826
**CSGALNACT1**	-0.378242123	0.13438926	-0.476613824	0.053080176
**TEK**	0.558630057	0.019760205	0.516358777	0.033833333
**ABHD17C**	-0.010661354	0.967606474	-0.230305969	0.373848645
**TEC**	-0.232352023	0.369499394	0.062683096	0.811107712
**PSMD5**	-0.233980605	0.366057791	-0.172539359	0.507839563
**ZFAND2A**	-0.421115216	0.092299467	-0.683209446	0.002501455
**LOC101927308**	-0.151400081	0.561880313	-0.268885109	0.296682562
**LINC00900**	-0.279286771	0.277654344	-0.311600992	0.223412721
**COTL1**	0.566362947	0.017776931	0.47388958	0.054648065

**SERTAD4-AS1**	-0.147845542	0.571204676	0.149742985	0.566219044
**AKAP8**	-0.075678593	0.772828322	-0.339221328	0.182854772
**GPR63**	-0.053907616	0.837193056	-0.593449232	0.012028566
**RSRP1**	-0.409380903	0.102722367	-0.572384994	0.016343342
**COMMD1**	-0.169365411	0.515794242	-0.343757652	0.176696849
**MRPL2**	-0.504046275	0.039106527	-0.634815508	0.006187465
**NAE1**	-0.42928568	0.085506222	-0.398309139	0.113300724
**GAL**	0.520212286	0.032300291	0.416965266	0.095894619
**LRRC49**	-0.19096388	0.462833474	-0.508178392	0.037271694
**CAPN7**	-0.532202135	0.027869009	-0.681153614	0.002608138
**AKAP10**	-0.105378326	0.68730493	-0.46158799	0.062161476
**LRP1**	0.292324405	0.254882559	-0.270000786	0.294605121
**SLC12A6**	0.289424528	0.259843668	0.60876455	0.009501425
**KDM2B**	-0.169640477	0.515102575	-0.331107418	0.194220793
**LOC100505978**	0.120507813	0.645009925	0.439581887	0.077470151
**PIGM**	-0.216632772	0.403633077	-0.567735619	0.017441804
**FUCA1**	0.151955117	0.560430304	0.245004207	0.343237325
**GALM**	0.272653784	0.289700293	0.812935784	7.28E-05
**TBC1D22A**	0.005076542	0.984572465	-0.22955797	0.3754457
**CHML**	-0.306379135	0.231679059	-0.447898495	0.071392761
**DDX50**	-0.423999846	0.089858247	-0.248054296	0.33707049
**LOC100507006**	-0.403985154	0.107785902	-0.574205076	0.015928441
**ZNF644**	-0.386151833	0.125776183	-0.648993477	0.004820807
**GIMAP7**	0.291889103	0.255623484	0.65942138	0.003980555

**SMYD5**	-0.539706611	0.025344131	-0.375858248	0.137063227
**GCLM**	-0.309748273	0.226323687	-0.423898271	0.08994341
**PLCE1**	-0.329573515	0.196420478	-0.560921209	0.019155422
**AMZ2**	0.186860202	0.472681258	0.256706412	0.319927738
**PRCC**	0.011442832	0.965233328	0.528227468	0.029282545
**ACSBG2**	-0.207351531	0.424552315	0.264466037	0.304997004
**SEPT1**	-0.278934314	0.278286621	-0.298441415	0.244612424
**LOC100506022**	-0.382465173	0.129741369	-0.279704086	0.27690685
**MECR**	-0.010639716	0.967672189	-0.429653828	0.085208902
**GPCPD1**	-0.360467605	0.155213587	0.08708533	0.73962915
**TTC38**	0.409917641	0.10222811	0.63507954	0.006159444
**GPRC5D**	-0.35046992	0.167841622	-0.470610959	0.05658054
**DZIP3**	-0.283897106	0.269464582	-0.392027307	0.119632829
**NUP37**	-0.640891706	0.005568002	-0.562513755	0.018743641
**PRELID1**	0.180819993	0.487361838	0.560677549	0.019219043
**PTCHD4**	-0.063478947	0.808750876	-0.524891318	0.030511044
**MTHFD2**	-0.180731525	0.487578483	-0.310068315	0.225819119
**VRK2**	-0.404334212	0.107453088	-0.496569137	0.042600138
**CPNE5**	-0.293752446	0.252461304	-0.353044749	0.164525439
**IFIT1**	0.463432619	0.060988707	0.543398636	0.024168842
**LOC100130987**	0.18303626	0.481949804	0.055472004	0.832530321
**CSK**	0.343583366	0.176930847	0.461315409	0.062336183
**HNMT**	0.589798615	0.01270309	0.504896188	0.03872364
**FAM96A**	0.261138175	0.311348757	0.642821316	0.005382101

**ZSCAN16**	-0.584559143	0.013723109	-0.192589687	0.458960522
**TMX2**	-0.178538884	0.492962734	-0.17314736	0.506322381
**AHCY**	0.033485432	0.898479376	0.549442808	0.022336484
**USP3-AS1**	-0.258780721	0.315895227	-0.541980142	0.024615285
**LOC100505716**	-0.518765659	0.032869428	-0.649749688	0.004755423
**HIST1H2BD**	0.101339163	0.698752707	0.510038898	0.036467288
**LOC101928820**	0.129923084	0.619189863	0.246829284	0.339539573
**FOXD3-AS1**	-0.081397171	0.756134052	0.153061941	0.55754363
**FAHD1**	0.535974281	0.026576724	0.737248563	0.000732999
**AKR1C3**	0.65615408	0.004229919	0.776969607	0.00024325
**ANO4**	-0.305196048	0.233578567	-0.369550397	0.144315314
**BLK**	-0.338584479	0.183730527	-0.628864215	0.006846783
**PHF13**	-0.399019327	0.112600036	-0.512338269	0.035491497
**PDZD3**	0.385472553	0.126500349	0.713554278	0.001297725
**DDX10**	0.18829142	0.469235018	0.475686681	0.053609952
**ALDH2**	0.250024299	0.333121592	0.714879989	0.001258742
**ARHGEF10L**	0.338741111	0.18351488	0.483666327	0.049177518
**FRMD1**	-0.275144134	0.285141277	-0.470357556	0.05673199
**GANC**	-0.445500828	0.073107706	-0.509593248	0.036658752
**VAC14-AS1**	-0.306756	0.23107605	-0.23843648	0.356733486
**ITSN1**	-0.295715766	0.249156	-0.608394828	0.009556976
**SUCLG1**	0.45388899	0.067237171	0.827557266	4.14E-05
**SQRDL**	-0.388940024	0.122834045	-0.403947064	0.107822264
**NSRP1**	-0.495661927	0.043039564	-0.491246086	0.045227585

**ACSL1**	0.336479172	0.18664539	0.512540399	0.035406681
**LOC101928524**	-0.191988562	0.460390606	-0.271541016	0.291751532
**ETNK2**	0.095754441	0.714682095	0.770596637	0.000294504
**MID1**	-0.002071641	0.99370402	-0.536566745	0.026378034
**CYB5RL**	0.27194683	0.291002458	-0.241900582	0.349578161
**VPS26B**	0.048754292	0.852588292	0.273276527	0.288556157
